# Influence of OSMAC-Based Cultivation in Metabolome and Anticancer Activity of Fungi Associated with the Brown Alga *Fucus vesiculosus*

**DOI:** 10.3390/md17010067

**Published:** 2019-01-19

**Authors:** Bicheng Fan, Delphine Parrot, Martina Blümel, Antje Labes, Deniz Tasdemir

**Affiliations:** 1 GEOMAR Centre for Marine Biotechnology (GEOMAR-Biotech), Research Unit Marine Natural Products Chemistry, GEOMAR Helmholtz Centre for Ocean Research Kiel, Am Kiel-Kanal 44, 24106 Kiel, Germany; bfan@geomar.de (B.F.); delphine.parrot@gmail.com (D.P.); mbluemel@geomar.de (M.B.); antje.labes@hs-flensburg.de (A.L.); 2 Faculty of Mathematics and Natural Sciences, Kiel University, Christian-Albrechts-Platz 4, 24118 Kiel, Germany

**Keywords:** Marine fungi, *Fucus vesiculosus*, OSMAC, molecular network, metabolomics, dereplication, bioactivity mapping

## Abstract

The fungi associated with marine algae are prolific sources of metabolites with high chemical diversity and bioactivity. In this study, we investigated culture-dependent fungal communities associated with the Baltic seaweed *Fucus vesiculosus*. Altogether, 55 epiphytic and endophytic fungi were isolated and identified. Twenty-six strains were selected for a small-scale One-Strain-Many-Compounds (OSMAC)-based fermentation in four media under solid and liquid culture regimes. In total, 208 fungal EtOAc extracts were tested for anticancer activity and general cytotoxicity. Ten most active strains (i.e., 80 extracts) were analyzed for their metabolome by molecular networking (MN), *in-silico* MS/MS fragmentation analysis (ISDB–UNPD), and manual dereplication. Thirty-six metabolites belonging to 25 chemical families were putatively annotated. The MN clearly distinguished the impact of culture conditions in chemical inventory and anticancer activity of the fungal extracts that was often associated with general toxicity. The bioactivity data were further mapped into MN to seek metabolites exclusively expressed in the active extracts. This is the first report of cultivable fungi associated with the Baltic *F. vesiculosus* that combined an OSMAC and an integrated MN-based untargeted metabolomics approaches for efficient assessment and visualization of the impact of the culture conditions on chemical space and anticancer potential of the fungi.

## 1. Introduction 

Marine fungi are found in all habitats and make a significant contribution to marine environments, e.g., by decomposition of substrates and animal remains. Marine fungi are regarded as saprotrophs, parasites, or symbionts (epiphytic an endophytic), as a consequence of the evolution of fungal cell biology and feeding strategies [1,2,3]. Recent culture-dependent studies reveal that fungi establish a symbiotic association with marine macroalgae (seaweeds) [1,2,3]. The sedentary lifestyle and the lack of an immune system have led to the evolution of a seaweed holobiont, in which macroalga and its associated microbiota, including fungi, jointly exercise to enhance the tolerance to abiotic and biotic stresses [4]. The algicolous fungi have been shown to produce diverse types of secondary metabolites, presumably contributing to the fitness of its host. Some of these metabolites have demonstrated bioactivities, e.g., antioxidant, antimicrobial, and cytotoxic [5,6,7] that are beneficial to human health. A prominent example is halimide, a diketopiperazine obtained from an *Aspergillus* sp. isolated from the green alga *Halimeda copiosa* [8]. Plinabulin (NPI-2358) is a synthetic tert-butyl analog of halimide and the only marine fungal metabolite-based compound that has entered clinical trials (currently in Phase III) to date [9]. This clearly highlights the potential of seaweed-derived fungi as a valuable source for discovery of anticancer lead compounds. 

Recent bioinformatic approaches in the genomics field points out a significant discrepancy between the number of secondary metabolite biosynthetic gene clusters (BGCs) and the actual number of chemically characterized compounds obtained from microorganisms. This stems from the so-called ‘silent’ or ‘cryptic’ BGCs that are not expressed under standard laboratory conditions due to a lack of essential natural stimuli for activation of secondary metabolite BGCs [10]. Hence, fermentation of microorganisms in artificial laboratory conditions represents a major limitation in microbial drug discovery. Culture-based strategies such as OSMAC (One-Strain-Many-Compounds) or co-cultivation are vital to evoke the expression of silent BGCs to produce new compounds [11]. The OSMAC approach is highly efficient for induction of chemical diversity by variation of cultivation parameters [12]. It has been successfully applied to stimulate the production of several new marine fungal secondary metabolites, exemplified by lajollamide A, a new pentapeptide produced by the marine green alga-derived fungus *Asteromyces cruciatus* [13]. This observation, in addition to numerous other OSMAC-based cultivation studies on microorganisms, suggest that variation of culture conditions is a promising approach for enhancing the chemical space of alga-derived fungi.

MS/MS based Global Natural Products Social Molecular Networking (GNPS) is a platform, which enables rapid and automated mass spectral mining of large number of samples. It groups similar compounds based on similarities of their MS/MS fragmentation patterns and links known and unknown compounds belonging to the same molecular family in networks [14]. This approach not only substantially accelerates dereplication capabilities and enhances annotation rates, but also delivers more in-depth information on chemical inventory of biological organisms [15]. Integration of MN with *in-silico* fragmentation pattern database (Universal Natural Product Database (ISDB–UNPD)) further speeds up the tedious dereplication processes [16]. A few recent studies have used molecular networks (MN) for optimizing the culture conditions and the extraction methods for bacterial natural products [17]. MN also offers the possibility to map additional information over the network. The integration of bioactivity data of crude extracts and fractions obtained therefrom with MN is gaining popularity for prioritization of bioactive extracts and targeted isolation of bioactive metabolites from botanical sources [18]. 

The canopy-forming seaweed *Fucus vesiculosus* L. (bladder wrack) is the most-widespread brown alga in the shallow coastal regions of the Baltic Sea [19]. It serves as a primary producer providing food and shelter for marine animals [20]. Previous studies conducted on this seaweed indicate its rich chemistry and various bioactivities [21]. In a recent study, we investigated seasonal variations in the metabolome of the Baltic Sea *F. vesiculosus* and its impact on antioxidant, anticancer and antimicrobial activity profile of the *F. vesiculosus* extracts [22]. However, the associated microbiota of this seaweed or their biotechnological potential has remained poorly investigated. The fungi derived from the North Atlantic *F. vesiculosus* were tested for antibacterial and antifungal activity [6,7]. An isobenzofuranone derivative with antioxidative activity was reported from an *Epicoccum* sp. isolated from the North Sea *F. vesiculosus* [23]. The present study aimed at identification of cultivable fungal communities associated with the Baltic *F. vesiculosus*. By using an OSMAC approach, the isolated epi- and endophytic fungi were grown in, altogether, eight solid and liquid-based culture media. The generated extracts were tested for their anticancer and cytotoxic properties and the selected ones were dereplicated by an untargeted LC-MS/MS-based molecular network using automated (GNPS and ISDB-UNPD) [14,16] and classical manual dereplication methods. The UPLC-MS/MS based MN revealed different culture conditions to lead to significant differences in chemical inventory and bioactivity of the extracts. The bioactivity data combined with MN allowed the detection of specific metabolites in the anticancer or toxic extracts. This is the first study on fungi associated with the Baltic Sea *F. vesiculosus* applying an untargeted metabolomics study integrated with OSMAC and bioactivity mapping.

## 2. Results

### 2.1. Isolation of Fungi

**Figure 1:**
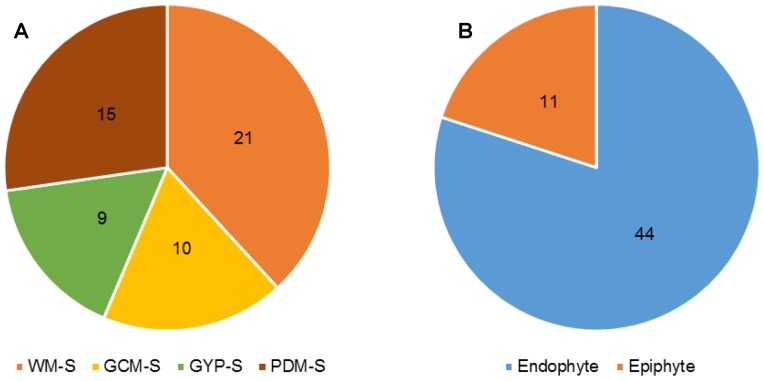
The number of identified fungal isolates from *Fucus vesiculosus*. (**A**) The number of fungal strains isolated from single solid medium (S). WM-S = modified Wickerham medium, PDM-S: Potato Dextrose medium, GCM-S; Glucose Casein medium, GYP-S; and Glucose Yeast Peptone medium. (**B**) The origin of fungi from *F. vesiculosus* microhabitats.

**Figure 2:**
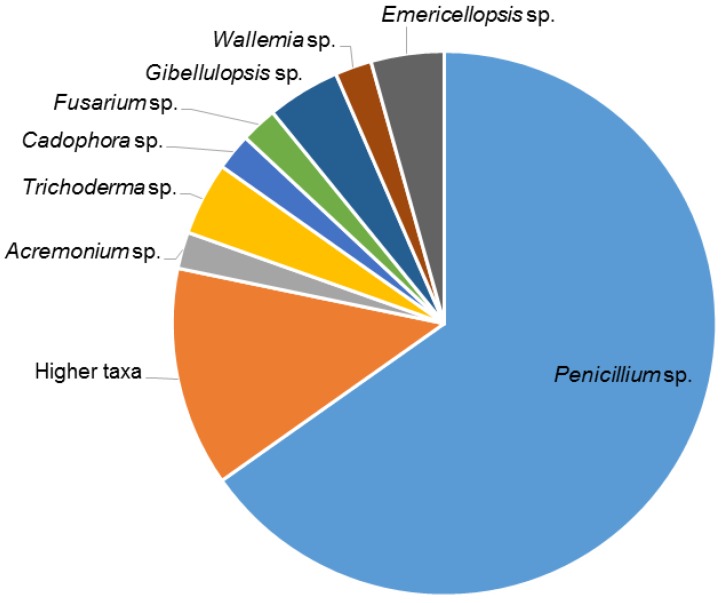
Taxonomical diversity of 55 fungal genera isolated from *F. vesiculosus* (excluding sediment and seawater). Strains that were not identified to genus level were indicated with ‘Higher taxa’.

**Figure 3:**
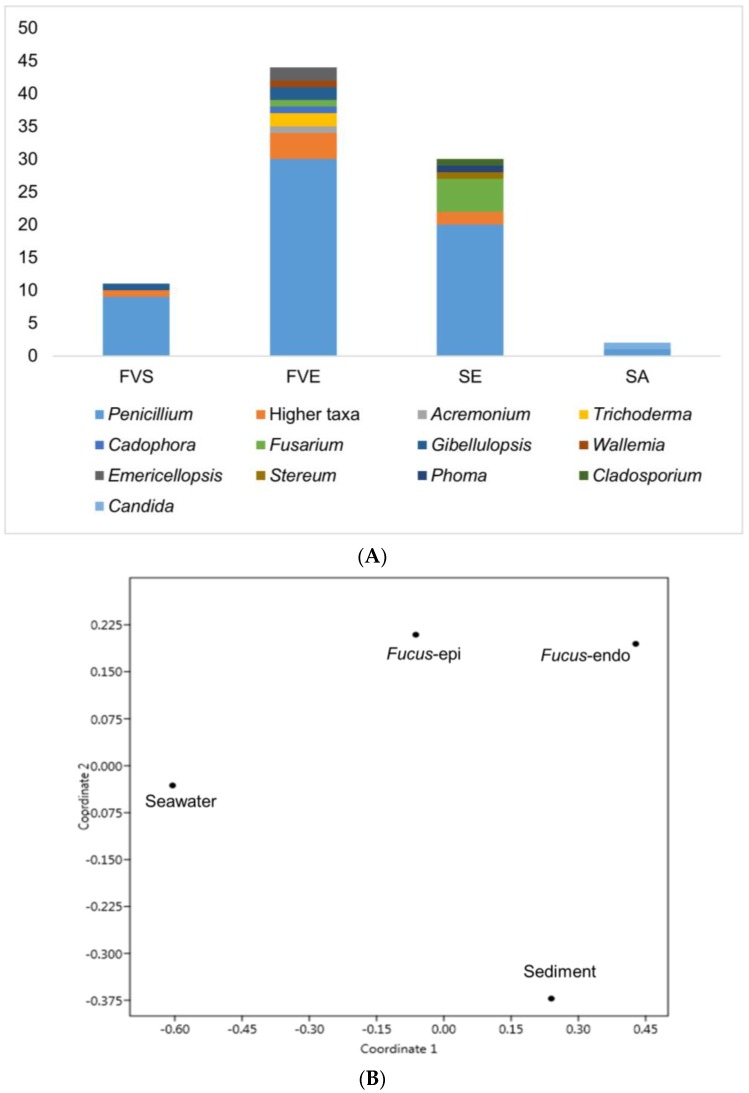
(**A**) Comparison of taxonomical diversity of the fungal isolates isolated from *F. vesiculosus* and its surrounding habitat. FVS = *F. vesiculosus* surface epiphytes, FVE = *F. vesiculosus* endophytes, SE = sediment, SA = seawater. (**B**) 2D MDS plot (Bray Curtis Similarity index) showing differences in the origin of fungi. MDS plot is based on a presence/absence of fungal genera (all 87 identified isolates)).

In total, 87 fungal strains were obtained (Table S1). Of which, 55 strains were isolated and identified from *F. vesiculosus* collected at Kiel Fjord (Baltic Sea, Germany), while 32 fungi originated from its surrounding environment, i.e.,—sediment (30 isolates) and seawater (2 isolates) controls. Four solid media (indicated by an “S” in the medium name), i.e., modified Wickerham medium (WM-S), Potato Dextrose medium (PDM-S), Glucose Yeast Peptone medium (GYP-S), and Glucose Casein medium (GCM-S) were used for their isolation. The highest number of fungal isolates was retrieved from WM-S medium (21 isolates), followed by PDM-S (15 isolates), GCM-S (10 isolates), and GYP-S (9 isolates) (Figure 1A and Figure S1). Overall, 11 epiphytic and 44 endophytic fungi were obtained from algal thalli after surface sterilization (Figure 1B). Sanger sequencing of the PCR-amplified ITS1-5.8S rRNA gene-ITS2 region allowed identification of 39 *Penicillium* sp. that constituted the largest fraction (71%) of the fungi. In general, a considerable fungal diversity was obtained with 10 strains belonging to seven fungal genera (identified at the genus level). Six isolates were identified only to a higher taxonomic (i.e., order) level (Figure 2). *Penicillium* was the predominant genus in both endophytic and epiphytic fungi. *Gibellulopsis* sp. was isolated from both endophytic and epiphytic communities. The endophytic fungi showed a four-fold higher abundance (44 isolates) and represented by five specific genera, i.e., *Acremonium*, *Trichoderma*, *Cadophora*, *Wallemia*, and *Emericellopsis* (Figure 3A). The media used had a great impact on the diversity of the isolated fungi. For example, four fungal genera (*Acremonium*, *Fusarium*, *Trichoderma* and *Wallemia*) were exclusively isolated from WM-S medium (Figure S2A). Other media also produced unique isolates, e.g., *Cadophora* sp. was only retrieved from GYP-S medium (Figure S2B). Both *Emericellopsis* sp. (WM-S and PDM-S, Figure S2A,C) and *Gibellulopsis* sp. were isolated from two different media (WM-S and GCM-S, Figure S2A,D). *Penicillium* species were obtained from all media, with PDM-S and GCM-S yielding the highest numbers (14 and 9 isolates, respectively) (Figure S2C,D).

The sediment control sample yielded 30 fungal strains, while only two isolates were retrieved from the seawater (Figure 3A). The genus *Penicillium* was also dominant among sediment isolates, accounting for 67% of the isolated strains (20 out of 30), followed by *Fusarium* sp. (5 isolates). Three genera, *Phoma*, *Cladosporium*, and *Stereum* were represented by one species each in sediment samples. Two other sediment-derived strains were only identified at order level (Figure 3A, Table S1). The seawater control yielded one *Penicillium* sp. and one *Candida* sp. Figure 3A depicts the comparative diversity of fungal isolates from *F. vesiculosus* and its surrounding habitats.

Several fungal genera, i.e., *Acremonium*, *Gibellulopsis*, *Trichoderma*, *Wallemia*, *Cadophora*, and *Emericellopsis* were exclusive to *F. vesiculosus*. The fungal communities isolated from different origins were compared by a 2D MDS plot to visualize their similarity (Figure 3B). All *Fucus*-derived fungal communities (*Fucus*-epi and *Fucus*-endo in Figure 3B) clustered more closely, showing a clear differentiation to those deriving from seawater and sediment controls.

### 2.2. OSMAC Cultivation, Extraction and Anticancer Assays

Twenty-three endophytic and three epiphytic fungal strains (Table 1) deriving from *F. vesiculosus* thalli were selected for subsequent small-scale fermentation using an OSMAC approach. The strain selection criteria were: (i) genetic diversity at genus or order level and (ii) morphology differences in colony (especially *Penicillium* sp.). Four different culture media, PDM, WM, Sucrose Yeast Medium (SYM), and Czapek (Cza) were selected based on variations they offer with respect to carbon source and salt concentration. Additionally, the solid (“S”, agar plates, static) and liquid (“L”, 300 mL flasks, shaking at 120 rpm) culture regimes were applied in order to enhance the chemical diversity of the selected strains. The temperature was set to 22 °C in all samples. A simple coding system including the strain number, growth medium, and culture regime was used to describe the extracts (e.g., 1PDM-L).

The selected strains were cultured, and overall, under eight different conditions, for two weeks and subsequently extracted with EtOAc to yield 208 crude extracts. These extracts were profiled for their in vitro activity against six cancer cell lines (liver cancer cell line HepG2, colorectal adenocarcinoma cell line HT29, malignant melanoma cell line A375, colon cancer cell line HCT116, lung carcinoma cell line A549, human breast cancer line MDA-MB231) and for toxicity against the noncancerous human keratinocyte cell line (HaCaT). Twenty-six samples that showed >90% growth inhibition at the initial test concentration (200 µg/mL) against at least one cancer line were selected and further tested for determination of their IC_50_ values. A threshold in anticancer activity (IC_50_ value ≤ 100 µg/mL against at least one cancer line) was set as a second filter for further prioritization of the extracts. Hence, 10 endophytic strains complying with this criterium were selected and grown in four liquid and four solid culture conditions to generate 80 extracts. Of these, only 16 extracts, all deriving from endophytic strains (Table 2) inhibited at least one cancer cell line with an IC_50_ value of ≤ 100 µg/mL, whereas the remaining 64 were inactive (IC_50_ > 100 µg/mL). Notably, 14 of the 16 fungal extracts that showed anticancer activity (IC_50_ values 2–100 µg/mL) also inhibited the growth of HaCaT cells with similar IC_50_ values, indicating a general toxicity. Only two crude extracts (1PDM-L and 58Cza-S) were active against cancer cell lines (IC_50_ values 39–98 µg/mL) and possessed no toxicity against HaCaT cell lines at 100 µg/mL concentration (Table 2). All sixteen extracts were selected for further studies.

The composition of the medium, as well as the culture regime, had a profound impact in bioactivity and toxicity of the fungi (Table 2). For example, the crude extracts of strains 1 (order Pleosporales), 35 (*Cadophora malorum*), 37 (order Hypocreales), 78 (*Penicillium* sp.), and 87 (order Pleosporales), inhibited the cancer cell lines only when fermented in liquid PDM medium (PDM-L). Similarly, strain 59 (order Glomerellales), cultivated only in PDM-S, displayed activity against four cancer cell lines, particularly against MDA-MB231 cells (IC_50_ value 14 µg/mL, Table 2). The remaining strains were either active in both liquid and solid cultures of one medium (e.g., *Penicillium* sp.—strain 68 in PDM), or in multiple media and culture conditions, e.g., strain 56 (*Penicillium* sp.) and strain 58 (*Fusarium graminearum*) (Table 2). In the case of strain 50 (*Penicillium* sp.), the extracts deriving from PDM-L and PDM-S media showed higher potency in comparison to that deriving from the Cza-S medium.

### 2.3. Evaluation of the Chemical Diversity Using Molecular Network Approach

**Figure 4:**
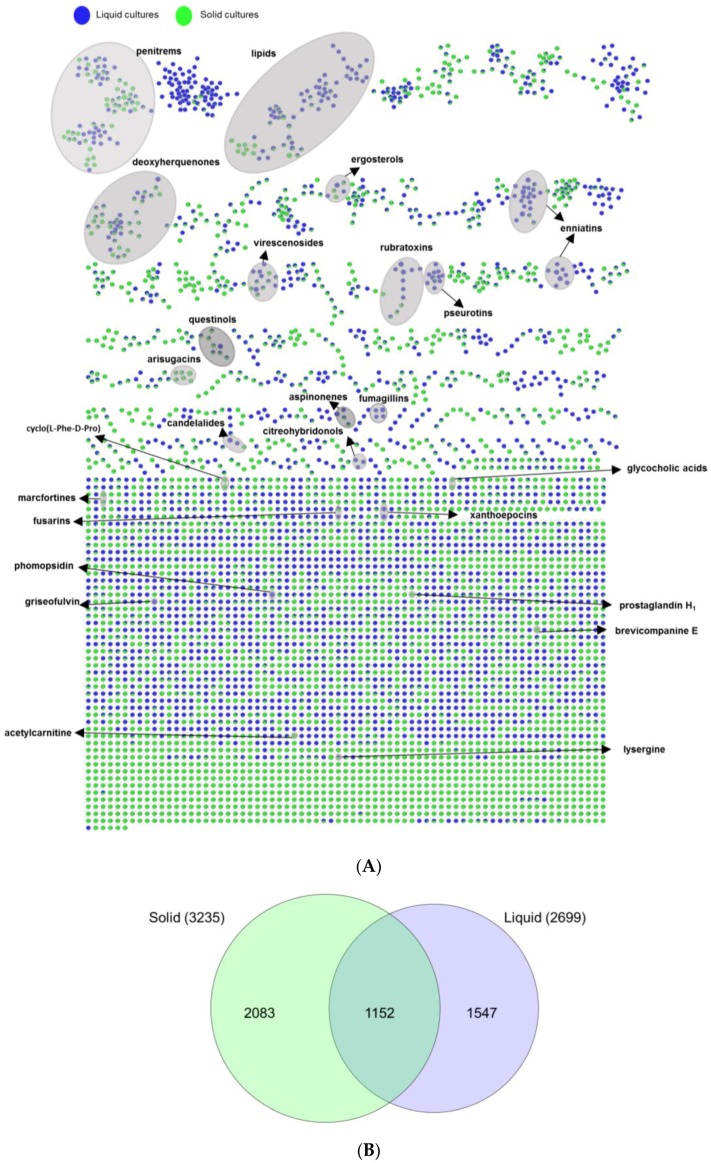
(**A**) Global molecular network of the extracts of 10 fungal strains grown in liquid and solid culture regimes. See Table S2 for annotated molecules. Green nodes: Ions detected in solid culture extracts. Blue nodes: Ions detected in liquid culture extracts. Grey loop: Nodes annotated as putatively known chemical clusters. (**B**) Euler diagram of the specific and shared ions detected in liquid and solid culture extracts.

An UPLC-QToF-MS/MS based comparative untargeted metabolomics study (in positive ionization mode) was carried out on those 10 initially selected strains (Figure S3). To ascertain the impact of media and culture regime on the chemical profile, as well as to detect potentially specific metabolites produced in the bioactive samples, 80 EtOAc extracts (10 strains grown in 8 culture conditions) were subjected to MN-based metabolomics analysis. The MS/MS data of the extracts were analyzed using the publicly available Global Natural Products Social Molecular Networking (GNPS) platform (https://gnps.ucsd.edu) [14] combined with both *in-silico* MS/MS database (ISDB-UNPD) [16] and manual dereplication employing multiple databases (Dictionary of Natural Products (DNP) [24], Scifinder [25], and Chemspider [26]). The global MN revealed 4782 nodes in total, of which 1700 were clustered in 255 chemical families, whereas the remaining single nodes were considered as unique chemistries without link to others. Some nodes represent adducts, thus, not all network nodes correspond to a single molecule. In the MN, 25 different chemical families were annotated (Figure 4, Figure 6, and Figure 7 and Table S2), including various types of alkaloids, polyketides, peptides, lipids, amino acid derivatives, dihydroxyanthraquinones, bile acids, polyenes, and binaphthoquinones. Characteristic information for all annotated peak ions to known metabolites (retention time, MS, MS/MS fragmentation, molecular formula, biological source and structure) is displayed in Table S2. The global MN of fungal extracts based on culture regime and growth media are shown in Figure 4A and Figure 6A, respectively. The global MN-derived Euler and Venn diagrams (Figure 4B and Figure 6B) display node (ion) distribution in different culture conditions, as discussed below. The chemical composition of each individual strain is displayed in a separate MN (Figures S4–S23).

#### 2.3.1. Impact of Culture Regime on Chemical Diversity

The effect of culture regime (liquid versus solid) was ascertained by comparing liquid and solid culture groups from the MN (Figure 4A). The Euler diagram derived from MN (Figure 4B) pointed out to clear differences in the chemical profile of the liquid and solid culture extracts. In total 4782 nodes (ions) were identified. The extracts deriving from the solid cultures (in total 3235 nodes) and the liquid cultures (in total 2699 nodes) had only 1152 nodes in common, indicating <24% similarity of total 4782 nodes (Figure 4B). Approximately, 32% of the total nodes (i.e., 1547 specific nodes) were expressed exclusively in the liquid culture extracts, while 2083 nodes (≈43%) in the solid culture extracts. The comparison of the liquid and solid culture-derived fungal extracts clearly points out a differential chemistry. The observed metabolic differences suggest activation of different BGCs under different culture regimes.

**Figure 5:**
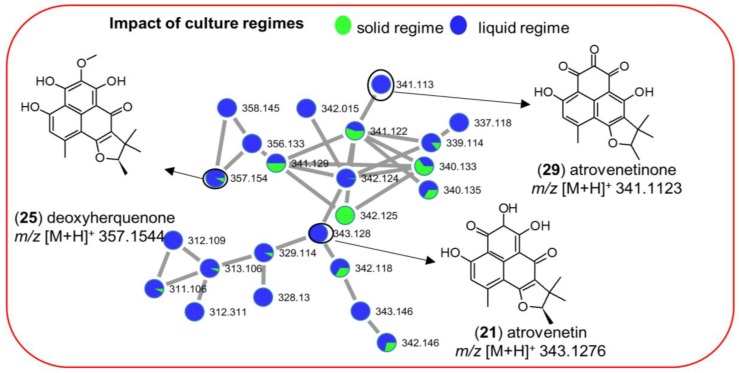
MN of the aromatic polyketide family detected in strain 68 (*Penicillium* sp.) extracts. Green: Ions detected in solid extracts. Blue: Ions detected in liquid extracts.

Annotation efforts of the chemical inventory of the extracts revealed only a few metabolites to be common in both liquid and solid cultures, e.g., the aminolipid family (**7**–**11**) and aromatic polyketide deoxyherquenone (**25**) (Figure 4A, Table S2) in both Cza-L and Cza-S media. Interestingly, several metabolites were mapped to only one culture regime. For example, the cyclic depsipeptide enniatin B_1_ (**34**) (Figure 4A and Figure S6) was detected only in liquid culture conditions. Importantly, their production was dependent on the culture regime, but was not much affected by the growth media. In addition to induction of different chemical families in individual culture regimes, we also observed production of compounds in both culture regimes. A good example is the aromatic polyketide family (**21**, **25**, **29**) that was detected in multiple extracts of strain 68 (*Penicillium* sp., Table S2). Deoxyherquenone (**25**, *m*/*z* [M + H]^+^ 357.1544) was present in all liquid and solid culture extracts, whereas its analogues atrovenetinone (**29**, *m*/*z* [M + H]^+^ 341.1123) and atrovenetin (**21**, *m*/*z* [M + H]^+^ 343.1276) were exclusive to liquid culture regimes. As shown in Figure 5, with 22 nodes observed in liquid culture-based extracts and 13 nodes in solid extracts of the strain 68, liquid culture conditions clearly led to a higher chemical diversity for this molecular family, clearly demonstrating the impact of the culture regime on variable chemical composition of the same strain.

#### 2.3.2. Impact of Culture Media on Chemical Diversity

**Figure 6:**
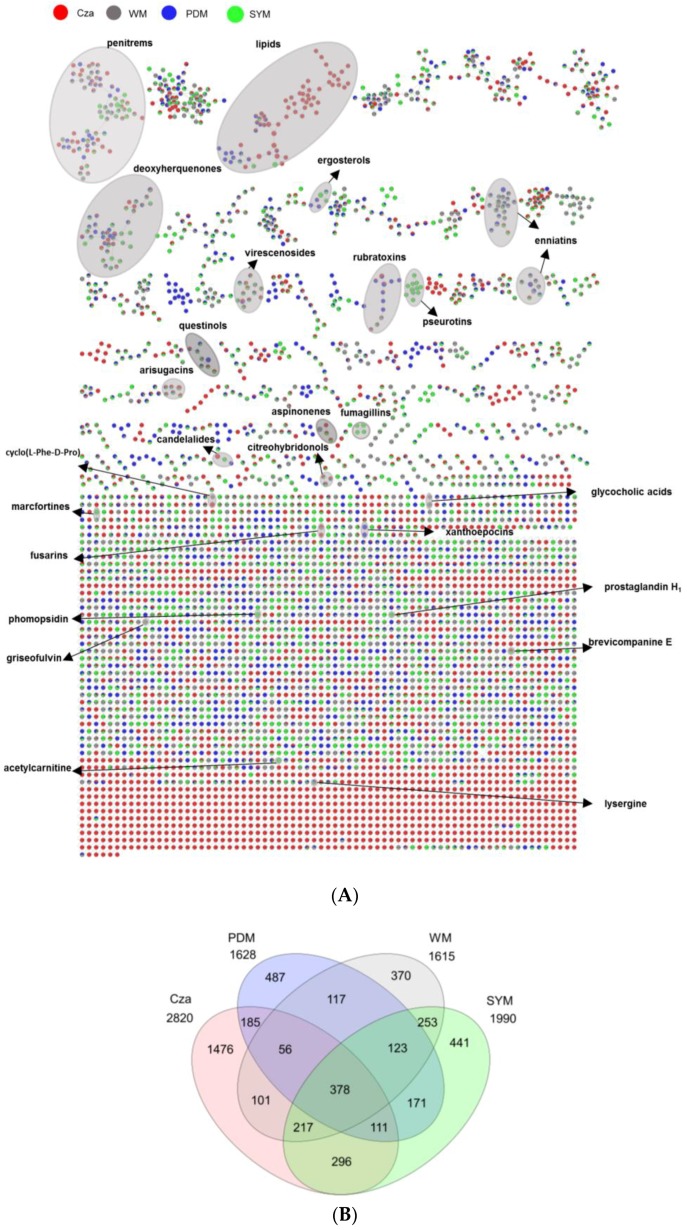
(**A**) Global MN of crude extracts of 10 fungal strains originating from 4 media (Cza, PDM, SYM, WM). See Table S2 for annotated molecules. Red: Nodes detected in Cza extracts. Blue: Nodes detected in PDM extracts. Green: Nodes detected in SYM extracts. Grey nodes: Nodes detected WM medium extracts. Grey loop: Nodes annotated as putatively known chemical clusters. (**B**) Venn diagram comparing the number of ions detected in different media. Cza (pink), PDM (blue), WM (grey), and SYM (green). Numbers of shared ions and specific ions only produced in one media-based extract were shown in differently shaded areas, while the total number of ions detected in each medium extract is shown underneath the medium name.

Next, we investigated the influence of culture media on the extracts of 10 selected strains (Figure 6A). The global MN-derived Venn diagram (Figure 6B) displays the distribution of altogether 4782 detected nodes. Notably, the Cza medium contained the highest number of nodes (2820), whereas the lowest number of nodes (1615) was detected in the WM medium. The Venn diagram also clearly indicated unique expression of significant numbers of nodes in one single culture medium. For example, 1476 of all nodes (ions) were specifically mapped to the Cza medium, corresponding to 31% of in total 4782 nodes. Significantly lower levels of nodes that were found to be exclusive to other media, i.e., 487 (PDM), 441 (SYM), and 370 (WM) (Figure 6B). These results confirmed that the chemical composition and other characteristics (e.g., selected carbon resource) of the growth media had a significant effect on the chemical machinery of the fungi derived from *F. vesiculosus*. The inspection of Table 2 indicated that most of the bioactive extracts that inhibited the growth of the cancer cell lines derived from the liquid PDM medium. Three extracts obtained from the Czapek culture also showed activities with lower potency.

The global MN of the crude extracts deriving from different media (Figure 6A) indicated variations in chemical diversity of the extracts. The annotated natural product clusters included e.g., fumagillin (**26**) type meroterpenoid mycotoxins, which were only expressed by strain 35 (*Cadophora malorum*) in SYM-L (Figure 6A, Figure S6 and Table S2), while the small binaphthoquinone family represented only by xanthoepocin (**19**) and bisdehydroxanthomegnin (**20**) that was exclusively mapped to PDM-L extract of strain 59 (order Glomerellales) (Figure 6A, Figure S16 and Table S2). Another striking example was the aminolipid family (**7**–**11**) produced by the same strain 59. This very large molecular cluster was expressed in both liquid and solid Cza media, with 52 of total 71 analogues with *m*/*z* ranging from 300 to 1300 Da exclusively mapped to the Cza medium (Table S2, Figure S24). The identification of this rare lipid family was based on the database search in GNPS, with a high spectral similarity score (>0.7) to our experimental MS/MS fragmentation pattern. The inspection of Figure 4 and Figure 6, the Supplementary Figures S4–S23 and Table S2 shows that, with the exception of the amide alkaloid pseurotin A (**6**) and the polyketide deoxyherquenone (**25**), all annotated chemical families/compounds are produced by one single strain often in one or more growth media. In most cases, both culture regime and culture media had an effect on chemical production, in which 15 of the total 36 known metabolites were exclusively annotated in one single culture medium under one single culture regime (Table S2). This was exemplified by the amino acid derivative acetylcarnitine (**1**, SYM-S) (Figure S5), the tetrahydrofuranone polyketide griseofulvin (**15**, WM-L) (Figure S18), and the indole diterpenoid penitrem B (**30**, SYM-S) (Figure S19). Despite the use of an integrated and detailed metabolomics approach, we were able to annotate only a few nodes to known compounds. This may suggest potential novelty of the remaining compounds.

#### 2.3.3. Bioactivity Mapping

**Figure 7:**
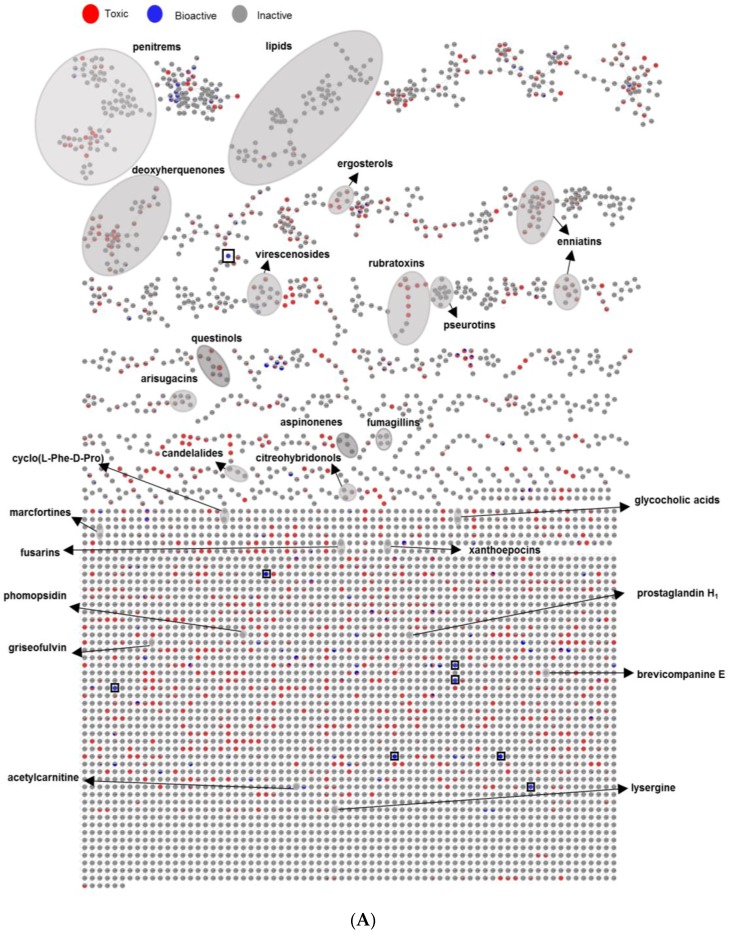

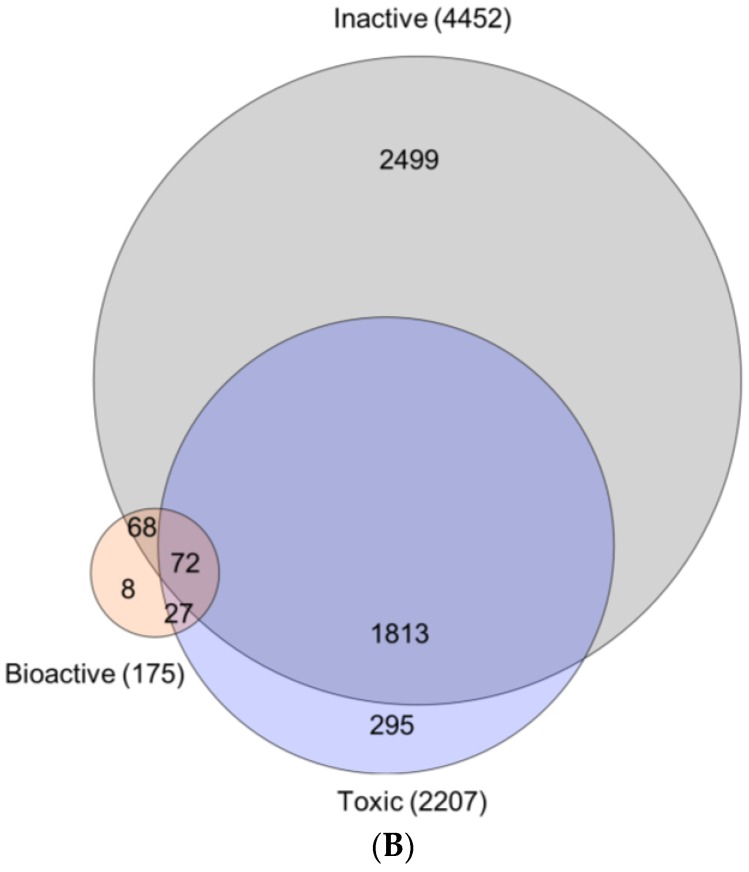
(**A**) Bioactivity (IC_50_ values) mapped to global MN of the extracts of 10 fungal strains (80 extracts). See Table S2 for annotated molecules. Red node: Ions detected in toxic samples (IC_50_ < 100 µg/mL towards both cancerous and non-cancerous HaCaT cell lines). Grey node: Ions detected in inactive samples (IC_50_ > 100 µg/mL towards cancer cell lines). Blue node: Ions detected in bioactive samples (IC_50_ ≤ 100 µg/mL towards cancer cell lines and IC_50_ > 100 µg/mL to HaCaT cell line). Grey loop: Nodes annotated as putatively known chemical clusters. Black squares: Eight nodes exclusively detected in bioactive (fully blue) extracts. (**B**) Euler diagram showing the specific and the shared ions in crude extracts. Red: Nodes detected in toxic samples. Grey: Nodes detected in inactive samples. Blue: Nodes detected in bioactive samples. Numbers of shared ions and specific ions are shown in differently shaded areas. The total number of ions for each group of extract is given in brackets.

As a last step, we mapped the bioactivity data (IC_50_ values) of altogether 80 extracts over the MN and searched for a link between spectral molecular networks and their anticancer activity (or toxicity). To this end, we classified the fungal extracts into three groups based on their anticancer activity (IC_50_ values), assigned them a specific color tag, and applied this color mapping to all nodes to establish a visible MN (Figure 7A). Thus i) 64 extracts that did not kill any cancer cell line in preliminary testing (IC_50_ > 100 µg/mL, see Section 2.2) were regarded as ‘inactive’ (tagged in grey), ii) 14 extracts that exhibited anticancer activity towards at least one cancer cell line and general toxicity to HaCaT cells with IC_50_ values < 100 µg/mL were treated as ‘toxic’ (tagged in red) and iii) 2 crude extracts (1PDM-L and 58Cza-S) that were devoid of toxicity against HaCaT cells (IC_50_ > 100 µg/mL) and killed at least one cancer cell line (IC_50_ < 100 µg/mL) were regarded as ‘bioactive’ (tagged in blue) (Figure 7A). Then, we explored the networks and compared the chemical composition of all three classes with different color mapping to all nodes in the MN. The rationale behind it is that if certain clusters/nodes are uniquely present in an extract, these compounds may possibly be responsible for the observed bioactivity and this extract(s) can be prioritized for further bioactivity-guided isolation studies.

As shown in the Euler diagram (Figure 7B), altogether 4452 nodes were detected in the inactive extracts, 2207 nodes in the toxic extracts, while the two bioactive extracts collectively comprised 175 nodes. Respectively, 2499 and 295 nodes were exclusive to the inactive and the toxic extracts. The bioactive extracts had only eight nodes that were specifically produced in them, but shared 27 nodes with the toxic and 68 with inactive extracts. Seventy-two nodes were common to all extracts.

The combination of massive MN with activity mapping allowed an easy visual inspection of the networks and their inhibitory activity against cancerous and non-cancerous cell lines. As shown in Figure 7A, the MN revealed eight fully exclusive (completely blue) ions for bioactive extracts 1PDM-L and 58Cza-S, five of which belonging to 1PDM-L and three of which belonging to 58Cza-S. Only one of these nodes identified from 1PDM-L (*m*/*z* [M + H]^+^ 428.2081, C_25_H_34_NO_5_) was embedded in a large molecular cluster containing 38 nodes (Figure 7A and Figure S25). The remaining seven were single nodes (single compound-single molecular family, Figure 7A), four of which being exclusive to 1PDM-L (*m*/*z* [M + Na]^+^ 452.2278 (C_25_H_35_NO_5_Na), *m*/*z* [M + H]^+^ 469.2242 (C_27_H_33_O_7_), *m*/*z* [M + H]^+^ 463.2679 (C_26_H_39_O_7_), *m*/*z* [M + H]^+^ 939.6554 (C_55_H_91_N_2_O_10_)) and 3 nodes being exclusive to 58Cza-S (*m*/*z* [M + H]^+^ 232.1525 (C_11_H_22_NO_4_), *m*/*z* [M + H]^+^ 681.3819 (C_31_H_57_N_2_O_14_), and *m*/*z* [M + H]^+^ 685.3978 (C_32_H_61_O_15_)). None of these eight nodes found a match to any known compound in databases, hence represent potentially new compounds that may be responsible or contributing to the bioactivity of these extracts. On the other hand, the blue nodes were also common to a number of networks that were toxic. This may suggest that the observed toxicity and anticancer activity is dependent on their final concentration in the extract and the chemical structure of the individual compounds and/or their molecular clusters. The fully toxic ions (fully red nodes) were represented by small networks and a number of single nodes. However, similar to bioactive samples, the toxicity was also embedded in many inactive (grey) networks. As expected, there were many fully inactive molecular clusters, but a large number of clusters included toxic and/or bioactive nodes, again pointing to general phenomenon of toxicity associated with anticancer activity.

## 3. Discussion

### 3.1. Fungal Isolation

Only a few studies have investigated endophytic fungi associated with the seaweed genus *Fucus* and their metabolites with antioxidant and antibiotic activities [6,7]. In this study, we isolated fungal assemblages present on the surface and in the inner tissues of the Baltic Sea *F. vesiculosus*. Isolation media (mostly favoring the growth of Ascomycetes) used herein were similar to those used for obtaining endophytic fungi from the North Sea *F. serratus* [1,27]. In accordance with previous studies, our isolates were also dominated by *Penicillium* sp. that are common in macroalgae [28]. The abundance of *Penicillium* sp. (39 isolates) in our study was even higher than that (14 isolates) observed in the previous study [1]. Reportedly, the abundance of *Penicillium* sp. in macroalgae is negatively correlated with water temperature [29]. Low water temperatures (8.3 °C) during our sampling campaign may provide a possible explanation for the higher abundance of *Penicillium* sp. *Vice versa*, the abundance of another very common genus in macroalgae, *Aspergillus*, is reportedly positively correlated with increasing temperatures [29], which may underlie the absence of *Aspergillus* sp. in our study. Zuccaro et al. [1] isolated only a few (5 isolates) *Aspergillus* sp. during their study on *F. serratus* for a full calendar year, suggesting an overall low abundance of the genus *Aspergillus* in *Fucus* species.

In addition, we identified four genera *Acremonium*, *Gibellulopsis*, *Trichoderma*, and *Emericellopsis* (Figure 3A) from the Baltic *F. vesiculosus*. Since they are absent in the sediment or seawater samples, they can be regarded as specific to the seaweed. These genera have also been isolated from the North Sea *F. serratus* samples [1]. A *Cadophora* sp. has previously been reported from the green alga *Enteromorpha* sp. [30], but not from any brown algae before. In the present study, we identified an endophytic *Wallemia* sp. This fungal genus is generally considered as xerophilic (adapted to dry conditions or high salinity) [31], and has previously been obtained from air, soil, dried food, and salt. To our knowledge, this is the first report of this genus being associated with a marine alga. The seawater control sample yielded a *Candida* sp., which is commonly found in seawater [32]. Finally, we isolated three fungal strains, *Cladosporium* sp., *Phoma* sp. and *Stereum* sp., that were exclusive to sediment surrounding *F. vesiculosus* (Figure 3A). *Cladosporium* and *Phoma* species are very common molds in sediment samples [33]. *Stereum* species belong to mycobiota of trees and known for wood-destroying and carbon recycling properties of arid forest ecosystems [34], but has never been isolated from a marine sediment before.

Interestingly, two known plant pathogens i.e., *Cadophora malorum* and *Fusarium graminearum* were also isolated from the inner tissues of *F. vesiculosus*. The presence of potential phytopathogens in macroalgae has already been described [1,27]. Those endophytes live in a healthy host resuming a saprophytic growth only during senescence of the hosts [1]. While their ecological role in *F. vesiculosus* remains elusive, these fungal genera that produce bioactive compounds, such as the hydroxylated sclerosporin derivatives reported from *Cadophora malorum* [30], may be involved in the well-being of the marine algae.

It is widely accepted that *Fucus* sp. employs various mechanisms to control their surface biofilm and microbiota. This includes mechanical means, i.e., cuticle shedding [35], production of reactive oxygen species (ROS) [36], and the release of secondary metabolites, such as the carotenoid fucoxanthin, onto the algal surface [37,38]. Such regulatory mechanisms of epiphytic control may underlie the 4-fold lower abundance of epiphytic fungi (11 isolates) compared to endophytic fungi (44 isolates). The diversity of epiphytic fungi was also poor and only three genera, *Gibellulopsis* (1 isolate), *Phoma* (1 isolate), and *Penicillium* (9 isolates) represented the cultivable epiphytic fungal community. Notably, none of the epiphytic fungi showed growth inhibitory activity against cancer cell lines in initial screenings, and hence, were not prioritized for in-depth metabolomics and IC_50_ determinations.

### 3.2. Evaluation of Chemical Diversity Under Different Culture Conditions

OSMAC is regarded as an essential method for activating silent BGCs to enhance the chemical diversity of microorganisms [39]. Here, we applied for the first time an OSMAC-based cultivation approach to *F. vesiculosus*-derived fungal strains, 10 of which inhibited the growth of multiple cancer cell lines in vitro. The UPLC-HRMS/MS-based untargeted metabolomics study by employing MN on 16 fungal extracts deriving from 10 selected fungal isolates revealed an enhanced chemical diversity, as shown in their respective MNs (Figures S4–S23). As discussed below, MNs provided opportunities to identify known compounds/molecular families and putatively new molecular families and to assess differences due to changes in the culture regime and the growth media. Overall, 36 metabolites belonging to 25 clusters were putatively identified, spanning from simple amino acids, fatty acid derivatives (including sphingolipids, aminolipids, aminoglycolipids), various types of alkaloids and polyketides, terpenoids, meroterpenoids, and steroids.

In this study, liquid and solid culture extracts only shared 24% nodes and more than 30% nodes were produced only under one single culture regime (Figure 4B). In-depth MN studies revealed that 16 putatively identified metabolites were exclusive to liquid culture regimes and 11 to solid regimes (Table S2). A similar MN-based metabolomics study conducted on marine bacterial extracts confirmed the strong impact of the culture regime on chemical diversity in which only 7% of the nodes were shared [17]. The differences in chemical production under different culture conditions may stem from changes in physical parameters, e.g., shaking that increases the availability of oxygen. As reviewed by Papagianni et al. [40], the production of metabolites in different morphological and physiological conditions is unique for each fungus. In this study, we applied shaking (120 rpm) to the liquid cultures, while the solid cultures were grown statically. Previous research on fungal antibiotics showed that fungi formed mycelial mat under static conditions and dispersed mycelia under shaking condition. The chemical production was affected by different mycelial growth conditions [41]. Our results confirmed differential biosynthesis of various types of metabolites in liquid (1547 specific nodes) and solid (2083 specific nodes) cultures (Figure 4B). Another factor affecting the fungal growth and metabolome is the availability of oxygen. The lack of oxygen in solid media has been shown to lead to impairment of the fungal growth and decreased production of fungal metabolites [42]. Conversely, shaking (in liquid conditions) facilitates aeration and the uptake of oxygen, thereby promoting fungal growth and potential accumulation of bioactive metabolites. While a lower number of nodes were expressed in the liquid culture extracts (i.e., 1547 specific nodes versus 2083 nodes in the solid extracts, 32 versus 43%, respectively) the liquid culture regime appeared more favorable for the expression of bioactive/cytotoxic metabolites than the solid regime. Many fungal mycotoxins, such as the enniatin type cyclic depsipeptides (**31**, **34**, **36**) (produced by strain 35), fumagillins (**26**), and the diterpene glycoside virescenoside E (**35**) (produced by strain 1), were exclusively expressed in liquid culture extracts. Ten of the total 16 active extracts with activity against cancer cell lines derive from liquid growth media and only six strains showed bioactivity against cancer cell lines when grown under solid static condition (Table 2). Expression of specific fungal metabolites under shaking has previously been observed by Guo and co-workers [43] where the switch of the culture condition of a *Penicillium* sp. from static to shaking led to the biosynthesis of five new nitrogen-containing sorbicillinoids [44]. Analogously, we detected 16 specific metabolites in liquid culture extracts, exemplified by the antifungal polyketide griseofulvin (Figure S18).

The culture media also played a significant role in the chemical diversity of the *F. vesiculosus* associated fungi. The Venn diagram (Figure 6B) showed that Cza medium provided not only chemically the most diverse extracts with the highest numbers of nodes (2820), but also the highest number of nodes (1476) exclusively produced in this medium. MN annotated specific metabolites that were produced in a single medium i.e., Cza (**4**, **8**, **9**, **10**, **11**, **32**), PDM (**5**, **19**, **20**), SYM (**1**, **14**, **26**, **28**, **30**, **33**), and WM (**2**, **12**, **15**) (Table S2). Annotation of metabolites that can be mapped exclusively to a certain medium suggests activation of silent BGCs due to varying culture parameters. This metabolism shift may be due to different nutrients used in this study, e.g., carbon, trace elements and salt. For example, Cza is the only medium containing sucrose (as carbon source) and trace metals (e.g., Mg^2+^) at the same time. The influence of carbon source on fungal metabolism is well known. For example only sucrose has stimulated the production of antibiotic metabolite bikaverin in *Fusarium fujikuroi* [45]. In the current work, the linear aminolipid family (**7**–**11**) produced by strain 59 (Table S2, Figure S24) was exclusively detected in the sucrose-containing medium Cza. Chatzifragkou and co-workers (2010) showed that carbon source affects the lipid production in fungi and the highest oleic acid concentration was obtained when a sucrose-based medium was used [46]. Additional research showed several enzymes that are involved in the regulation of lipid synthesis, e.g., ATP citrate lyase, were dependent of Mg^2+^ for their activities [47,48]. The use of Cza as medium for culturing the strain 59 (order Glomerellales) resulted in the highest number of metabolites (2820 nodes) and highest percentage (31%) of compounds unique to this medium, as a response to the presence of specific nutrients, e.g., trace elements and sucrose.

PDM was the only medium containing a mixed carbon source, i.e., the polymer starch and the monomer dextrose. The anti-inflammatory metabolite dihydroxyanthraquinone questinol (**5**) [49] was previously isolated from the marine derived fungus *Eurotium amstelodami* incubated in a starch-based SWS medium. The strain 50 (*Penicillium* sp.) exclusively produced this compound in the starch containing medium PDM (Figure S10), pointing out the importance of the polymeric starch as carbon resource. The total number of nodes in PDM extracts was 1628, of which 487 were exclusive to this medium. Only a few nodes mapped to PDM were annotated to known metabolites, this suggests that fungal strains are able to produce a rich number of putative new natural products when cultured in a PDM medium. Importantly, most of the extracts with growth inhibitory activity against cancerous (and non-cancerous) cell lines were grown in solid or liquid PDM (Table 2). Thus, all 10 strains showed low IC_50_ values (<100 µg/mL) against cancer cell lines in PDM (six fungi only in PDM-L, one fungus only in PDM-S, and three strains—50, 58, and 68 in both PDM-L and PDM-S) (Table 2).

The third medium, SYM, contained sucrose and yeast extract as nutrient provider. These two components appeared suitable for production of mycotoxins. There were altogether 1990 nodes detected in SYM extracts, with 441 nodes being exclusive. Previous research on *Aspergillus flavus* showed that the absence of yeast extract led to silencing of mycotoxin production in fungi [44]. In the present study, several known fungal mycotoxins families, such as the small cluster of fumagillin family (**26** produced by strain 35, Figure S6) and a very large cluster of penitrem family (**30**, produced by strain 68, Figure S19) were assigned to be exclusive to SYM by analysis of the MN.

The last medium, WM contains high concentrations of salt (NaCl 30 g/L). As shown in Figure 6B, 1615 nodes in total were observed in WM extracts, 370 of these being specific to this medium. One such compound is griseofulvin (**15**), a chlorinated tetrahydrofuranone polyketide produced by the strain 68 (Figure S18). Griseofulvin was only annotated from the 68WM-L extract, potentially as a response to high Cl^+^ concentration. Glucose is an excellent carbon resource for fungal growth, however, it may repress the transcription of biosynthetic genes, such as *pcb*AB, *pcb*C, and *pen*DE in *Penicillium* sp. [50] resulting in a poor metabolite diversity. Finally the presence of salts may impair the growth of some fungi, leading to a poor chemical composition. This could be another factor contributing to the low chemical diversity and lack of bioactivity of the crude extracts derived from salt-containing media, SYM and WM.

It is important to mention that the chemical production of strains was not only affected by the culture regime but also by the culture media. The most striking example is represented by the indole diterpenoid penitrem B (**30**). A previous study showed that penitrems were only produced under static conditions and enhanced production occurs upon addition of sucrose and yeast extract into medium [51]. Accordingly, we detected the penitrem B (**30**), exclusively, in solid SYM culture of strain 68 (*Penicillium* sp.) (Figure S19). This indicates that both culture regime and culture media strongly affect fungal chemical diversity of the fungi. Our research that employed detailed metabolomics by MN and bioactivity testing underlines the complexity of the media effect on fungal metabolism and bioactivity as a result of it. Overall, the OSMAC approach applied here suggests PDM-L as the best culture condition for a high chemical diversity coupled with cancer growth inhibitory activity in *F. vesiculosus*-derived fungi. While the Cza medium provided the highest chemical diversity and exclusiveness in metabolites, only a few extracts deriving from this medium showed generally low anticancer activity associated with toxicity. It is very likely that these extracts/induced compounds have other biological activities, which warrants further screenings in future.

### 3.3. Bioactivity

Based on the proven anticancer potential of algal-derived fungi [9], we focused on the assessment of the anticancer activity and general toxicity of *F. vesiculosus* derived fungi. An IC_50_ value ≤ 100 μg/mL was set to define the bioactivity of the crude extracts. The changes in the fungal metabolism due to composition of the culture medium and/or the culture regime played a very important role in the observed bioactivity of the crude extracts. Hence, out of 80 extracts, only 16 fulfilled the IC_50_ value criterium (≤100 μg/mL). Some extracts showed significant variations in their potency against different cancer cells. For example, 50Cza-S was inactive against HepG2 and HT29 cells, but showed good IC_50_ values against other cancerous cell lines, in particular towards MDA-MB231. However, the majority of the extracts suffered from the lack of selectivity, as their anticancer activity was often comparable to their general toxicity towards non-cancerous HaCaT cells. As exemplified by doxorubicin, the standard drug used as a positive control herein (Table 2), toxicity is a well-known general side effect of anticancer drugs. Only two extracts 1PDM-L and 58Cza-S lacked toxicity at the highest test concentrations (IC_50_ >100 μg/mL), but their anticancer effect was also moderate.

Our dereplication study indicated the presence of some known chemical families, including mycotoxins, in the fungal extracts. Indeed, we annotated 11 individual mycotoxins belonging to eight chemical families that were previously reported as fungal mycotoxins. These include the amide alkaloid pseurotin A (**6**), polyketide griseofulvin (**15**), polyene fusarin C (**16**), bis-anhydrides rubratoxins A (**18**) and B (**17**), meroterpenoid fumagillin (**26**), indole diterpenoid penitrem B (**30**), cyclic depsipeptides enniatins B, B_1_, and A (**31**, **34**, **36**), and the diterpene glycoside virescenoside E (**35**). Several mycotoxins with demonstrated anticancer and/or cytotoxic activities (the existing literature mostly measured their anticancer activity without assessing their toxicity) were detected, and their bioactivity was often dependent on the culture conditions. Rubratoxins are common mycotoxins of *Penicillium* sp. [52,53] with specific protein phosphatase A2 inhibitory and anticancer/antimetastasis activities [54]. Rubratoxins A (**18**, *m*/*z* [M + H]^+^ 521.1890) and B (**17**, *m*/*z* [M + H]^+^ 519.1832) were only annotated in the toxic PDM-L and Cza-L extracts of strain 50 (*Penicillium* sp.) (Table S2, Figure S10). This molecular cluster contained four additional unannotated nodes (*m*/*z* [M + H]^+^ 598.1872, 577.1812, 767.2503, and 768.2531), which were only induced in this strain when incubated in PDM-L medium (Figure S10). These results suggest that culture conditions enhance the expression of fungal BGCs to induce biosynthesis of certain molecular families with new, potentially bioactive congeners. Enniatin type mycotoxins (**31**, **34**, **36**) with known anticancer properties [55] were also abundant in the toxic PDM-L extract of strain 35 (*Cadophora malorum*) (Figure S6). Other mycotoxin families, e.g., fumagillins (**26**) and pseurotins (**6**) with known anticancer/cytotoxic activity were expressed in 35SYM-L and 78WM-S, which lacked anticancer activity. The bioactivity mapping further proved this, as many toxic/anticancer mycotoxin clusters (fumagillins, pseurotins, penitrems, etc) were detected in grey colored networks belonging to inactive extracts. This may stem from their low concentrations in the extract and/or overall chemical composition of these extracts.

MN has found successful applications in drug discovery, in the form of bioactivity mapping and bioactivity-based MN for prioritization of extracts and targeted isolation of bioactive natural products [17,56]. In this study, we applied OSMAC approach with varied growth media and culture regime and mapped its results on MN for assessing both bioactivity and molecular profile of the extracts. The crude extracts were classified into three different groups based on their IC_50_ values (≤100 μg/mL) and toxicity to HaCaT. The layout assigned color tags to each group, which allowed i) rapid and efficient visualization and analysis of molecular clusters, and ii) analysis of whether these specific cluster of nodes were restricted to certain strain, growth medium, or culture regime. By using this visible network, we attempted to detect unique metabolites produced in bioactive samples. Toxicity is the main limitation in anticancer drug discovery. Accordingly, 14 extracts showed significant toxicity whereas the extracts derived from the strain 1 (order Pleosporales) in PDM-L and strain 58 (*Fusarium graminearum*) in Cza-S media showed moderate anticancer potential with no toxicity at 100 μg/mL. MN-based bioactivity mapping identified 8 unannotated nodes to be exclusively present in these two extracts, indicating their potential impact in the observed activity. Then, we attempted to annotate known metabolites in these bioactive extracts. The only secondary metabolite we could annotate in the 1PDM-L extract was the diterpene glycoside virescenoside E (**35**, Figure S4), which is regarded as a fungal mycotoxin [57]. This compound belonged to a small, four-membered molecular cluster, however, none of the compounds in this cluster were unique to 1PDM-L, so their significant contribution to bioactivity is unclear. We were unable to dereplicate any nodes in the other selectively active extract 58Cza-S. However, we found one significant cluster in this crude extract (Figure S26) that was distributed in all three groups of extracts (inactive, toxic and bioactive). Dereplication efforts on three nodes specific to 58Cza-S, i.e., *m*/*z* [M + H]^+^ 401.3221, [M + H]^+^ 403.3355, [M + H]^+^ 419.3300, and their MS spectral fragments did not match any known compounds in the databases used. These three compounds are closely related and may potentially represent new metabolites.

Besides the mycotoxins described above, a few other dereplicated metabolites reportedly exerting anticancer activity were mapped to toxic or inactive extracts. For example, ergosterol (**28**), the major steroidal constituent of fungal membranes with inhibitory activity against several cancer cell lines [58] was detected in the inactive 35SYM-L extract (Figure S6). Phomopsidin (**33**) is a polyketide that interacts with microtubules [59]. This compound that was embedded in a small molecular family was annotated and originated from the inactive sample 87SYM-L (Figure S22). We also observed various chemical families without reported anticancer activity. This was exemplified by the sesterterpenoid citreohybridonol (**13**, *m*/*z* 501.2903, [M + H]^+^) identified from strain 68 (*Penicillium* sp.) grown in several solid-liquid media, however only the 68PDM-S showed activity associated with strong toxicity (Table S2, Figure S18).

In conclusion, we have isolated and identified, for the first time, a diverse cultivable fungal community associated with the Baltic Sea *F. vesiculosus*. The use of an OSMAC approach combined with efficient and modern tools such as MN coupled with bioactivity mapping, enabled the identification of optimal culture conditions and clear visual comparison of the chemical diversity and bioactivity of the extracts. Based on this important information, strain 1 (order Pleosporales) was prioritized for large-scale fermentation to be followed by purification and structure elucidation of its bioactive components.

## 4. Materials and Methods

### 4.1. General Experimental Procedures

EtOAc (used for fungal extraction) was purchased from VWR International GmbH, Hannover, Germany. UPLC grade methanol, acetonitrile and water used for UPLC/MS analysis were purchased from BiosolveChimie, Dieuze, France. Formic acid (UPLC/MS optigrade) was obtained from LGC Standards Promochem©, Wesel, Germany. UPLC-QToF-MS/MS analyses were carried out on an ACQUITY UPLC I-Class System coupled to the Xevo G2-XS QToF Mass Spectrometer (Waters^®^, Milford, MA, USA). Czapek broth, yeast extracts and malt extracts were purchased from BD Bioscience, Sparks, NE, USA. Agar was purchased from Applichem, Darmstadt, Germany. Peptone from soymeal and glucose were purchased from Merck, Darmstadt, Germany. Potato extract was from Sigma-Aldrich, Schnelldorf, Germany. Sucrose was purchased from Handelsmarken, Offenburg, Germany. Casein hydrolysate was purchased from Carl Roth, Karlsruhe, Germany.

### 4.2. Sampling and Isolation of Fungi

All fungi were isolated and identified from *F. vesiculosus* (Class: Phaeophyceae, Order: Fucales, Family: Fucaceae) specimens collected in Falckenstein Beach (54°23′22.6″ N, 10°11′26.4″ E), Kiel Fjord, Baltic Sea, Germany in December 2015 (pH 7, water temperature 8.3 °C). For isolation of fungal strains, four solid media were used: modified Wickerham medium (WM: NaCl 30 g, glucose 10 g, peptone from soymeal 5 g, yeast extract 3 g, malt extract 3 g, agar 15 g for 1 L), Glucose Yeast Peptone medium (GPY: glucose monohydrate 1 g, peptone 0.5 g, yeast extract 0.1 g, sodium chloride 15 g, agar 15 g for 1 L; pH 7.2), Potato Dextrose medium (PDM: Potato extract 4 g, dextrose 20 g, agar 15 g for 1 L; pH 5.6), and Glucose Casein medium (GCM: casein hydrolysate 2.5 g, glucose 40 g, MgSO_4_ 0.1 g, KH_2_PO_4_ 1.8 g, agar 15 g for 1 L pH 6.8) [60,61]. All media were enriched with 100μg/mL streptomycin and 100 μg/mL of penicillin to suppress bacterial growth. Before plating, the macroalgal specimens were washed three times with sterilized saline solution (0.9 g/L NaCl). Epibionts were obtained from the surface of conceptacles, stipe, and holdfast regions by sterile cotton swabs. For isolation of endophytic fungi, the algal surface was disinfected by 40% ethanol and 1% sodium hypochlorite for 1 min [62]. Algal tissues from conceptacles, stipe and holdfast regions were cut into approximately 4 mm × 1 mm fragments. Each fragment was put into a 2 mL innuSPEED lysis tube type S containing 0.4–0.6 mm ceramic beads (Analytik Jena, Jena, Germany) and 600 μL of sterilized 0.9% saline. Samples were homogenized for about 2 min at 15 Hertz (15/s) with the RetschMM200 mixer mill (RetschGmbHH, Haan, Germany) to release the endophytes from tissue to saline. Low speed mixing was pursued in order to prevent destruction of the endophytic fungal cells. After homogenization, two dilutions of mixture (containing endophyte fungi and saline), namely 10^−1^ and 10^−2^ were prepared. An aliquot (100 μL) of the dilutions as well as of the undiluted sample were inoculated on the above-mentioned 4 agar media and spread evenly using drigalski spatula. Surface sterilized tissues of *F. vesiculosus* was swabbed again and incubated on the same 4 solid media to evaluate the effects of surface sterilization. Samples were incubated for 14 days at 22 °C and checked for fungal growth twice per week. Fungal colonies were transferred to new plates and incubated until pure cultures were obtained. For cryopreservation, the pure fungal strains were transferred to cryobank tubes (MAST Diagnostica, Reinfeld, Germany) and stored at −100 °C following the manufacturer’s instructions.

### 4.3. Identification of Fungal Strains

Fungal communities were identified based on the nuclear ribosomal internal transcribed spacer (ITS) region containing the ITS1 and ITS2 regions, which frame the 5.8S rRNA gene. The ITS region is generally accepted as a universal phylogenetic marker for fungi [63]. DNA extraction was performed according to the protocol published previously [64]. PCR amplification was performed using primers ITS1F and ITS4R, in a total reaction volume of 25 µL, consisting of 1 μL of template DNA, 1 μL of each primer (concentration: 10 μM), 12.5 μL Dream Taq Master mix (ThermoFisherScientific, Schwerte, Germany), and 9.5 μL DNA free water (ThermoFisher Scientific, Schwerte, Germany). DNA free water was used as negative control for PCR. The following protocol was used in a Biometra T1 thermocycler for amplification: initial denaturation for 85 s at 94 °C, followed by 30 cycles of denaturation of DNA at 94 °C for 35 s, primer annealing at 55 °C for 55 s, and elongation for 3 min at 72 °C. A final elongation step for 10 min at 72 °C completed the PCR. The correct length of PCR products was checked by gel electrophoresis on a 1% agarose gel run for 20 min at a voltage of 120 V in 1× TBE buffer, taking 100 bp plus DNA ladder (Thermo Scientific™ GeneRuler™, Sunnyvale, CA, USA) length standard. PCR products were submitted for Sanger sequencing to the Institute of Clinical Molecular Biology (IKMB) at Kiel University applying the same primers as used for PCR. Obtained sequences were checked for quality and trimmed using Chromas Pro (Technelysium Pty Ltd., South Brisbane, Australia). Sequences were then compared to the NCBI Genbank (https://www.ncbi.nlm.nih.gov/genbank/) using the Nucleotide BLAST function. Sequences were stored at GenBank and the accession numbers are displayed in Table S1. An MDS plot showing differences between samples (seawater, sediment, *Fucus* epiphytic, *Fucus* endophytic) was generated using the statistical software PAST based on a presence/absence matrix of identified fungal genera in 87 identified isolates [65].

### 4.4. OSMAC-Based Cultivation of Fungi

The fungi were incubated in four different media, which were chosen based on variations in their carbon and nitrogen sources and salt concentration: The modified Wickerham medium (WM), Czapek medium (Cza: sucrose 30 g, KH_2_PO_4_ 1 g, MgSO_4_ 0.5 g, KCl 0.5 g, Fe_2_(SO_4_)_3_ 0.01 g for 1 L; pH 7.3), Potato Dextrose medium (PDM), and Sucrose Yeast medium (SYM: sucrose 20 g, yeast extract 10 g, NaCl 10 g for 1L; pH 5.2) [61]. These media were selected in order to provide different nutrient regimes to activate biosynthetic gene clusters [12]. Each fungal strain was incubated in all four media by applying two different culture regimes (solid and liquid). The solid culturing contained 15 g/L agar as solidifying agent and liquid media (100 mL medium in 300 mL Erlenmeyer flasks). Solid cultures were incubated at 22 °C for 14 days in the dark under static conditions. The liquid cultures were incubated on an orbital shaker (VKS-75 control, Edmund Bühler, Hechingen, Germany) agitating at 120 rpm at 22 °C for 14 days in the dark.

### 4.5. Extraction

For extraction of the liquid cultures, 100 mL of culture broth were mixed with the same volume of EtOAc by means of an ultra-turrax (Miccra, Mülheim, Germany) at 19,000 rpm. The mycelia were filtered by folded filter cellulose membrane type 113P (100% cellulose membrane 210 mm, Carl Roth, Karlsruhe, Germany). The filtrate was collected into a glass separatory funnel and left for phase separation. The lower (water) phase was discarded. 40 mL of Milli-Q water (Arium^®^ Water Purification Systems, Sartorius, Germany) was added to the organic phase to remove salts and water-soluble compounds. After phase separation, the water phase was discarded, and the EtOAc phase was transferred into 40 mL glass tubes. The extracts were evaporated to dryness by a Speed-Vac concentrator (RVC2-33, Martin Christ Gefriertrocknungsanlagen, Osterode am Harz, Germany). The dissolved crude extracts were re-dissolved in 2.5 mL MeOH and transferred into 4 mL pre-weighted HPLC vials (amber glass, VWR, International GmbH, Hannover, Germany). For chemical analysis, 0.1 mg/mL of extract was prepared in an ULC/MS grade MeOH and transferred into 1.5 mL vials (amber glass, VWR, International GmbH, Hannover, Germany). The dissolved extracts were filtered through 0.20 µm PTFE syringe filter (Carl Roth, Karlsruhe, Germany) before injection. Solid fungal cultures were sliced into small pieces (approximately 1 cm^2^) with a flat spatula and transferred into a 250 mL glass flask and homogenized with 100 mL EtOAc by means of an ultra-turrax at 19,000 rpm for 30 s. The extraction process for solid cultures followed the same steps as applied for liquid cultures. The media blanks were prepared by extracting liquid and solid media using the same protocol.

### 4.6. Chemical Analysis

#### 4.6.1. UPLC-QToF-MS/MS Analysis

The extracts were prepared at final concentration of 0.1 mg/mL and injected (2 µL) into an Acquity UPLC HSS T3 column (High Strength Silica C18, 1.8 µm, 2.1 × 100 mm, Waters^®^) operating at 40 °C. A mobile phase system (A: 0.1% formic acid in 99.9% ULC/MS grade water and B: 0.1% formic acid in 99.9% acetonitrile) was pumped at a flow rate of 0.6 mL/min using the following linear gradient: initial, 99% A–1% B; 0–11.5 min, 0% A–100% B; 11.5–12.5 min 0% A–100% B, and finally a column reconditioning phase until 15 min. The total run time was 15 min. MS and MS/MS spectra, in positive mode, were recorded during the UPLC run with the following conditions: capillary voltage: 0.8 kV, sample cone voltage: 40.0 V, source temperature: 150 °C, desolvation temperature: 550 °C, cone gas flow: 50 L/h and desolvation gas flow: 1200 L/h. A scan range was performed from 50 to 1600 Da, MS^2^ fragmentation was achieved with ramp collision energy (CE): Low CE from 6–60 eV and a high CE of 9–80 eV. The solvent (MeOH) and non-inoculated blank media were used as controls. MS and MS^2^ data were acquired and analyzed with MassLynx^®^ software (Waters^®^, V4.1, Waters, Milford, MA, USA).

#### 4.6.2. Molecular Network and Data Analysis

All MS/MS data were converted from files (.raw) to mzXML file format using the ProteoWizard tool MSConvert (version 3.0.10051, Vanderbilt University, Nashville, TE, USA) [66]. The data were uploaded to GNPS server (gnps.ucsd.edu) [14] by using FileZilla (https://filezilla-project.org/) [67]. All mzXML data were analyzed using the molecular networking workflow. A molecular network was created using the online workflow at GNPS. The data was filtered by removing all MS/MS peaks within +/− 17 Da of the precursor *m*/*z*. The data were then clustered with MS-Cluster with a parent mass tolerance of 0.1 Da and a MS/MS fragment ion tolerance of 0.1 Da to create consensus spectra. Further, consensus spectra that contained less than 2 spectra were discarded. A network was then created where the edges were filtered to have a cosine score above 0.65 and more than 6 matched peaks. Further edges between two nodes were kept in the network if and only if each of the nodes appeared in each other’s respective top 10 most similar nodes. The spectra in the network were then searched against GNPS spectral libraries. The library spectra were filtered in the same manner as the input data. All matches kept between network spectra and library spectra were required to have a score above 0.65 and at least 6 matched peaks. The clustered data were downloaded and imported into Cytoscape^®^ (version 3.5.1, Institute for Systems Biology, Seattle, WA, USA) [68] and visualized using import network and table, individual (selfloop) nodes without neighbors were also displayed in molecular network. Nodes derived from the blank media and methanol blank as well as solvent nodes were subtracted. Self-loop nodes (single node without link to others) were also displayed in molecular network. Venn diagram and Euler diagram were drawn by Venn and Euler Diagrams app available as a plugin for Cytoscape^®^. To improve the dereplication, the *in-silico* MS/MS database based on the Universal Natural Products Database (UNDP) was integrated into the MN following the workflow from Allard et al. [16] The parent mass was selected as dereplication prefilter. Setting was as following: Top_K_Result was used to return maximal number of results. Parameters were selected as following: PM tolerance = 0.05, score threshold = 0.2, Top K Result = 3. Only natural products reported isolated from fungal resources were selected as putative hits. The manual annotation was done by using the multiple databases, such as Dictionary of Natural Products (DNP) [24], Scifinder (https://www.cas.org/products/scifinder) [25] and Chemspider [26] based on *m*/*z* value, ppm shift.

### 4.7. Anticancer Activity

The crude extracts were initially tested in vitro against 6 human cancer cell lines; liver cancer cell line HepG2 (DSMZ, Braunschweig, Germany), colorectal adenocarcinoma cell line HT-29 (DSMZ, Braunschweig, Germany), malignant melanoma cell line A-375 (CLS, Eppelheim, Germany), colon cancer cell line HCT-116 (DSMZ, Braunschweig, Germany), lung carcinoma cell line A549 (CLS, Eppelheim, Germany), human breast cancer line MDA-MB-231 (CLS, Eppelheim, Germany), and the non-cancerous human keratinocyte line HaCaT (CLS, Eppelheim, Germany) at a concentration of 200 µg/mL. The antitumoral activity of the crude extracts was evaluated by monitoring the metabolic activity using the CellTiterBlue Cell Viability Assay (Promega, Mannheim, Germany). HepG2, HT-29 and HaCaT cells were cultivated in RPMI medium, A549 and MDA-MB-231 cells in DMEM:Ham’s F12 medium (1:1) supplemented with 15mM HEPES and A-375 and HCT-116 cells in DMEM medium supplemented with 4.5 g/L D-Glucose and 110 mg/L sodium pyruvate. All media were supplemented with l-Glutamine, 10% fetal bovine serum, 100 U/mL penicillin, and 100 mg/mL streptomycin. The cultures were maintained at 37 °C under a humidified atmosphere and 5% CO_2_. The cell lines were transferred every 3 or 4 days. For experimental procedure, the cells were seeded in 96 well plates at a concentration of 10,000 cells per well. A stock solution of 40 mg/mL in DMSO was prepared of each extract. After 24 h incubation, the medium was removed from the cells and 100 µl fresh medium containing the test samples was added. Each sample was prepared in duplicate once. Doxorubicin as a standard therapeutic drug was used as positive control, 0.5% DMSO and growth media were used as controls. Following compound addition, plates were cultured at 37 °C for 24 h. Afterwards, the assay was performed according to the manufacturer’s instructions and measured using the microplate reader Tecan Infinite M200 at excitation 560 nm and emission of 590 nm. For the determination of IC_50_ values, a dilution series of the extracts was prepared and tested, as described before. The IC_50_ values were calculated by Excel as the concentration that shows 50% inhibition of the viability on the basis of a negative control (no compound) and compared with the positive control (doxorubicin).

## Figures and Tables

**Table 1:** The list of 26 *F. vesiculosus*-derived fungal isolates selected for OSMAC-based cultivation. Strain I.D. numbers correspond to those used in strain collection in Table S1. Closest relative identification showing high sequence similarity according to NCBI GenBank by BLAST and the original isolation medium are displayed. * Strain could not be identified to genus level, but to a higher taxa (order) level.

Strain I.D.	Epiphytic/Endophytic Fungus	Closest Taxonomical Relative	Sequence Similarity (%)	Isolation Solid Medium
1 *	Endophytic	Pleosporales (order)	100	WM-S
11	Epiphytic	*Gibellulopsis* sp.	100	GCM-S
12	Endophytic	*Acremonium* sp.	99	WM-S
35	Endophytic	*Cadophora malorum*	100	GYP-S
37 *	Endophytic	Hypocreales (order)	99	GYP-S
38	Endophytic	*Penicillium biourgeianum*	100	WM-S
50	Endophytic	*Penicillium* sp.	99	PDM-S
55	Endophytic	*Penicillium glabrum*	100	PDM-S
56	Endophytic	*Penicillium* sp.	100	WM-S
58	Endophytic	*Fusarium graminearum*	96	WM-S
59 *	Endophytic	Glomerellales (order)	100	WM-S
61	Endophytic	*Penicillium brevicompactum*	100	PDM-S
62	Endophytic	*Penicillium brevicompactum*	100	PDM-S
67	Endophytic	*Gibellulopsis nigrescens*	100	WM-S
68	Endophytic	*Penicillium* sp.	100	WM-S
78	Endophytic	*Penicillium* sp.	100	PDM-S
81	Endophytic	*Wallemia muriae*	100	WM-S
82	Endophytic	*Emericellopsis* sp.	99	WM-S
84 *	Endophytic	Hypocreales (order)	100	WM-S
86	Endophytic	*Emericellopsis terricola*	99	PDM-S
87 *	Endophytic	Pleosporales (order)	99	WM-S
89	Endophytic	*Trichoderma* sp.	100	WM-S
91	Endophytic	*Penicillium virgatum*	100	GCM-S
96	Epiphytic	*Penicillium brevicompactum*	100	GYP-S
104	Epiphytic	*Penicillium chrysogenum*	100	WM-S
122	Endophytic	*Penicillium brevicompactum*	100	GCM-S

**Table 2:** List of 16 extracts deriving from 10 fungi with IC_50_ values ≤ 100 µg/mL against at least one cancer line. Crude extracts were tested against six cancer cell lines (human liver cancer cell line HepG2, human colorectal adenocarcinoma cell line HT29, human malignant melanoma cell line A375, human colon cancer cell line HCT116, human lung carcinoma cell line A549 and human breast cancer line MDA-MB231) and the non-cancerous human keratinocyte cell line HaCaT. Stand.: Standard for positive control (doxorubicin). Strain I.D. corresponds to strain collection I.D. in Table S1.

Strain I.D.	Taxonomic ID	Medium & Regime	IC_50_ Value (μg/mL)						
			HepG2	HT29	A375	HCT116	A549	MDA-MB231	HaCaT
1	Pleosporales	PDM-L	47	39	>100	>100	98	49	>100
35	*Cadophora malorum*	PDM-L	15	50	4	6	16	35	5
37	Hypocreales	PDM-L	11	27	5	7	18	7	8
50	*Penicillium* sp.	PDM-L	11	100	8	8	15	7	9
		PDM-S	6	16	29	8	10	2	2
		Cza-S	>100	>100	53	23	23	6	4
56	*Penicillium* sp.	PDM-L	43	36	66	>100	42	35	69
		Cza-L	100	100	63	>100	40	32	32
58	*Fusarium graminearum*	PDM-L	74	69	72	74	85	78	78
		PDM-S	69	63	59	69	71	62	44
		Cza-S	65	79	52	65	74	74	>100
59	Glomerellales	PDM-S	>100	>100	42	32	83	14	30
68	*Penicillium* sp.	PDM-L	10	32	8	7	10	5	8
		PDM-S	12	18	28	6	9	3	3
78	*Penicillium* sp.	PDM-L	17	21	15	25	19	9	10
87	Pleosporales	PDM-L	43	35	56	91	36	30	60
Stand.			14.9	3	0.13	10.6	31.4	15.2	10

## Supplementary Materials

The following are available online at http://www.mdpi.com/1660-3397/17/1/67/s1, Table S1: Identification of 87 fungal strains from *F. vesiculosus* and its surrounding environment (sediment and seawater). Table S2: Putatively identified compounds from 80 culture extracts. Figures S1–S2: Strains isolated and identified from *F. vesiculosus* and its surroundings. Figure S3: Workflow for the OSMAC study based on the 55 fungal isolates derived from *F. vesiculosus*. Figure S4–S23: Annotated molecular network for crude extracts of different strains under different culture regime and culture media. Figure S24: Molecular network of the aminolipid family of strain 59 (order Glomerellales) extracts. Figure S25. Molecular network of unannotated cluster containing one specific node. Figure S26: Molecular network of unannotated cluster containing 3 specific nodes in 58Cza-S.
