# Supplementary material for: Influence of OSMAC-Based Cultivation in Metabolome and Anticancer Activity of Fungi Associated with the Brown Alga Fucus vesiculosus
Source: Mar Drugs. 2019 Jan 19;17(1):67. doi: 10.3390/md17010067 (PMC6356420; doi:10.3390/md17010067)
Supplement: Supplementary file 1 [file marinedrugs-17-00067-s001.pdf]

marine drugs

Supplementary Materials

Influence of OSMAC-Based Cultivation in Metabolome and Anticancer Activity of Fungi Associated with the Brown Alga *Fucus vesiculosus*

Bicheng Fan [1], Delphine Parrot [1], Martina Blümel [1] Antje Labes [1,†] and Deniz Tasdemir [1,2,*]

1 GEOMAR Centre for Marine Biotechnology, Research Unit Marine Natural Products Chemistry, GEOMAR Helmholtz Centre for Ocean Research Kiel, Am Kiel-Kanal 44, 24106 Kiel, Germany; bfan@geomar.de (B.F.); delphine.parrot@gmail.com (D.P.); mbluemel@geomar.de (M.B.); antje.labes@hs-flensburg.de (A.L.)

2 Faculty of Mathematics and Natural Sciences, Kiel University, Christian-Albrechts-Platz 4, 24118 Kiel, Germany

† Current address: Flensburg University of Applied Sciences, Department Energy and Biotechnology, Kanzleistraße 91-93, 24943 Flensburg, Germany

* Correspondence: dtasdemir@geomar.de; Tel.: +49-431-600-4430

Table S1. Identification of 87 fungal strains isolated from *Fucus vesiculosus* and its surrounding environment (sediment and seawater). Identification was done according to Sanger sequencing comparison of the ITS1-5.8S rRNA gene-ITS2 fragment to NCBI GenBank nucleotide database using BLASTn (incl. respective assigned Genbank accession numbers). The 3 first BLAST hits incl. respective accession numbers are given. WM: Modified Wickerham medium, PDM: Potato Dextrose medium, GCM: Glucose Casein medium, GYP: Glucose Yeast Peptone medium. SE: sediment. SA: seawater. FVE: *F. vesiculosus* endophytic fungi. FVS: *F. vesiculosus* surface epiphytic fungi. *Strain could not be identified to genus level but only to higher taxa (order) level.

**Series no.**	**I.D.**	**Source**	**Isolation Medium**	**Sequence length**	**Closest relative in NCBI GenBank**	**NCBI accession number**	**Order**	**Sequence similarity %**	**Accession number**
**1***	1	FVE	WM	297	*Pyrenochaetopsis* sp. HKU62	LC158605.1	Pleosporales	100	MH881440
					Pyrenochaetopsis sp. CBS:119739	LT623227.1			
					Uncultured *Leptosphaeria* sp.2KK-2015	KP747710.1			
**2**	2	SE	PDM	172	*Fusarium lateritium* 3821	MG066631.1	Hypocreales	100	MH881441
					Fusarium tricinctum IBL277f	MF162319.1			
					Fusarium avenaceum SFC101774	MF186082.1			
**3**	6	SE	GCM	261	*Fusarium culmorum* F165	KU891563.1	Hypocreales	100	MH881442
					Fusarium graminearum PGTU10 18S	MF497389.1			
					Fusarium culmorum Ba4	MH361213.1			
**4**	8	SE	PDM	270	*Penicillium sanguifluum* IG103	MG973279.1	Eurotiales	100	MH881443
					Penicillium sp. S1a1	KY784181.1			
					Penicillium sanguifluum 17_3N1	KY859378.1			
**5***	10	SE	GYP	426	*Pyrenochaetopsis microspora* MUT4941	LT623227.1	Pleosporales	100	MH881444
					Roussoellaceae sp. MUT4859	KR014355.1			
					Phoma sp. MUT 5462	KU314985.1			
**6**	11	FVS	GCM	385	*Gibellulopsis* sp. MYf203	KX079890.1	Hypocreales	100	MH881445
					Gibellulopsis sp. P2	KY465972.1			
					Gibellulopsis nigrescens LG1401	KX359602.1			
**7**	12	FVE	WM	400	*Acremonium furcatum* MUT1194	KR709189.1	Hypocreales	99	MH881446
					Acremonium antarcticum AY913	LT549083.1			
					Acremonium antarcticum CBS 987.87	JX158422.1			
**8**	13	FVE	WM	301	*Trichoderma gamsii* SCAU130	MF061792.1	Hypocreales	100	MH881447
					Trichoderma koningii ACCC32855	MF780862.1			
					Trichoderma koningii ACCC32853	MF780861.1			
**9**	14	SE	WM	306	*Penicillium* sp. L2-3C	MG813426.1	Eurotiales	100	MH881448
					Penicillium sp. UCD161305F	MG686509.1			

					Penicillium thomii MUT<ITA>:2257	MG813165.1			
**10**	15	SE	WM	227	*Penicillium* sp. L2-3C	MG813426.1	Eurotiales	100	MH881449
					Penicillium sp. UCD161305F	MG686509.1			
					Penicillium thomii MUT<ITA>:2257	MG813165.1			
**11**	16	SE	PDM	358	*Penicillium glabrum* KAS5827	KY469043.1	Eurotiales	100	MH881450
					Penicillium glabrum SFC101259	MF185992.1			
					Penicillium spinulosum Su-XII-4	MF475933.1			
**12**	20	SE	PDM	287	*Penicillium* sp. L2-3C	MG813426.1	Eurotiales	100	MH881451
					Penicillium sp. UCD161305F	MG686509.1			
					Penicillium thomii MUT<ITA>:2257	MG813165.1			
**13**	24	SE	GCM	376	*Penicillium murcianum* PM1	MF668969.1	Eurotiales	100	MH881452
					Penicillium nucicola KAS 2101	KT887846.1			
					Penicillium sp. SFC102007	MF186110.1			
**14**	25	SE	GYP	482	*Cladosporium sloanii* DTO:130-D5	MF473253.1	Eurotiales	98	MH881453
					Cladosporium psychrotolerans DTO:307-H2	MF473224.1			
					Cladosporium psychrotolerans DTO:305-G3	MF473223.1			
**15**	26	SE	GCM	232	*Penicillium* sp. EMA-2011d	JF429677.1	Eurotiales	99	MH881454
					Penicillium thomii MUT<ITA>:2257	MG813165.1			
					Penicillium glabrum SFC101259	MF185992.1			
**16**	27	SE	GYP	352	*Penicillium coralligerum* YK247	LC214562.1	Eurotiales	99	MH881455
					Penicillium atrovenetum bqw5	MF599167.1			
					Penicillium sp. 8	KY401130.1			
**17**	29	SE	GYP	430	*Penicillium glabrum* KAS5827	KY469043.1	Eurotiales	100	MH881456
					Penicillium glabrum SFC10125	MF185992.1			
					Penicillium glabrum SYPF 6991	MH279474.1			
**18**	30	SE	GYP	375	*Penicillium glabrum* KAS5827	KY469043.1	Eurotiales	100	MH881457
					Penicillium glabrum SFC10125	MF185992.1			
					Penicillium glabrum SYPF 6991	MH279474.1			
**19**	31	SE	GYP	338	*Penicillium brevicompactum* GIBI220	KX965655.1	Eurotiales	100	MH881458
					Penicillium sp. M_FA_V8	MH137760.1			
					Penicillium brevicompactum	MH047201.1			
**20**	32	FVE	PDM	366	*Penicillium brevicompactum* MERVA5	MF503895.1	Eurotiales	100	MH881459
					Penicillium brevicompactum	MH047201.1			
					Penicillium brevicompactum KAS5854	KY469047.1			
**21**	33	FVE	PDM	318	*Penicillium atrovenetum* bqw5	MF599167.1	Eurotiales	100	MH881460

					Penicillium atrovenetum CBS 243.56	KP016835.1			
					Penicillium sp. MT152	MH109377.1			
**22**	34	FVE	GYP	468	*Penicillium atrovenetum* bqw5	MF599167.1	Eurotiales	100	MH881461
					Penicillium coralligerum YK-247	LC214562.1			
					Penicillium antarcticum SFC101809	MF186085.1			
**23**	35	FVE	GYP	323	*Cadophora malorum* M7	MG813381.1	Incertae sedis	100	MH881462
					Cadophora malorum VKM F-4744	MF494613.1			
					Cadophora malorum VKM F-4747	MF494620.1			
**24***	37	FVE	GYP	304	*Emericellopsis* sp. SNT1-23	KY379579.1	Hypocreales	99	MH881463
					Acremonium zonatum CSR1-21	KY379556.1			
					Sarocladium kiliense 11-84	KX815337.1			
**25**	38	FVE	WM	328	*Penicillium biourgeianum* UWR031	KX426968.1	Eurotiales	100	MH881464
					Penicillium bialowiezensec SFC101475	MF186021.1			
					Penicillium bialowiezensec KAS5860	KY469051.1			
**26**	39	FVE	WM	224	*Penicillium brevicompactum* UWR031	KX426968.1	Eurotiales	100	MH881465
					Penicillium bialowiezense KAS5860	KY469051.1			
					Penicillium brevicompactum 82	KY401133.1			
**27**	40	FVE	WM	347	*Penicillium* sp. 86	KY401137.1	Eurotiales	100	MH881466
					Penicillium atrovenetum BQW5	MF599167.1			
					Penicillium sp. 8	KY401130.1			
**28**	42	FVE	GYP	517	*Penicillium brevicompactum* F27-02	KX664363.1	Eurotiales	100	MH881467
					Penicillium brevicompactum KAS5854	KY469047.1			
					Penicillium brevicompactum KAS5812	KY469041.1			
**29**	43	SE	WM	228	*Penicillium* sp. L2-3C	MG813426.1	Eurotiales	100	MH881468
					Penicillium sp. UCD161305F	MG686509.1			
					Penicillium thomii MUT<ITA>:2257	MG813165.1			
**30**	44	SE	PDM	206	*Fusarium chlamydosporum* CFFSUR-22A	KY860655.1	Hypocreales	100	MH881469
					Fusarium oxysporum f. sp. momordicae GuangX26	MF445489.1			
					Fusarium chlamydosporum Ng30	MH141316.1			
**31**	46	SE	PDM	355	*Penicillium glabrum* KAS5827	KY469043.1	Eurotiales	100	MH881470
					Penicillium glabrum SFC101259	MF185992.1			
					Penicillium glabrum SFC101229	MF185985.1			
**32**	47	SE	PDM	372	*Penicillium glabrum* KAS5767	KY469033.1	Eurotiales	100	MH881471
					Penicillium glabrum SFC101259	MF185992.1			

					Penicillium glabrum PO6	KY463488.1			
**33**	48	SE	PDM	365	*Penicillium jensenii* P6342	MH063657.1	Eurotiales	100	MH881472
					*Penicillium sp.*1217_476	MG917754.1			
					Penicillium canescens 3S.106	KY458474.1			
**34**	49	SE	GYP	307	*Penicillium jensenii* P6342	MH063657.1	Eurotiales	100	MH881473
					*Penicillium sp.*1217_476	MG917754.1			
					Penicillium canescens 3S.106	KY458474.1			
**35**	50	FVE	PDM	507	*Penicillium* sp. 86	KY401137.1	Eurotiales	100	MH881474
					Penicillium sanguifluum IG103	MG973279.1			
					Penicillium sp. 8	KY401131.1			
**36**	52	SE	WM	322	*Penicillium* sp. L2-3C	MG813426.1	Eurotiales	100	MH881475
					Penicillium sp. UCD161305F	MG686509.1			
					Penicillium thomii MUT<ITA>:2257	MG813165.1			
**37***	53	FVS	GYP	314	*Phoma* sp. MUT5465	KU314987.1	Pleosporales	99	MH881476
					Roussoellaceae sp. MUT 4859	KR014355.1			
					Phoma sp. MUT 5462	KU314985.1			
**38**	54	FVS	GCM	337	*Penicillium brevicompactum* F27-02	KX664363.1	Eurotiales	100	MH881477
					Penicillium sp. UCD160901G3	MG686505.1			
					Penicillium brevicompactum KAS5854	KY469047.1			
**39**	55	FVE	PDM	300	*Penicillium glabrum* ND70	MG659664.1	Eurotiales	100	MH881478
					Penicillium thomii MUT<ITA>:2257	MG813165.1			
					Penicillium glabrum SFC101229	MF185985.1			
**40**	56	FVE	WM	523	*Penicillium coralligerum* YK-247	LC214562.1	Eurotiales	100	MH881479
					Penicillium sp. 86	KY401137.1			
					Penicillium sp. 76	KY401126.1			
**41**	57	FVE	GYP	331	*Penicillium atrovenetum* FO.1	KT587349.1	Eurotiales	100	MH881480
					Penicillium antarcticum SFC101809	MF186085.1			
					Penicillium coralligerum YK-247	LC214562.1			
**42**	58	FVE	WM	258	*Fusarium graminearum* WF1	KY985465.1	Hypocreales	96	MH881481
					Fusarium graminearum PGTU11	MF497390.1			
					Fusarium graminearum PGTU10	MF497389.1			
**43***	59	FVE	WM	231	*Gibellulopsis nigrescens* LG1402	KY459202.1	Glomerellales	100	MH881482
					Plectosphaerella sp. sedF4	KX359602.1			
					Uncultured Verticillium JB40C25 (MOTU06)	HG935585.1			
**44**	60	FVE	WM	310	*Penicillium* coralligerum YK-247	LC214562.1	Eurotiales	100	MH881483

					Penicillium atrovenetum bqw5	MF599167.1			
					Penicillium sp. 86	KY401137.1			
**45**	61	FVE	PDM	514	*Penicillium brevicompactum* TCDFvLDB1814	MH71548.1	Eurotiales	100	MH881484
					Penicillium brevicompactum Asa3SNA1	KY558614.1			
					Penicillium brevicompactum KAS5854	KY469047.1			
**46**	62	FVE	PDM	324	*Penicillium brevicompactum* F27-02	KX664363.1	Eurotiales	100	MH881485
					Penicillium brevicompactum KAS5854	KY469047.1			
					Penicillium brevicompactum KAS5812	KY469041.1			
**47**	63	FVE	PDM	334	*Penicillium brevicompactum* F27-02	KX664363.1	Eurotiales	100	MH881486
					Penicillium brevicompactum KAS5854	KY469047.1			
					Penicillium brevicompactum KAS5812	KY469041.1			
**48**	64	FVE	PDM	341	*Penicillium brevicompactum* MERV5	MF503895.1	Eurotiales	100	MH881487
					Penicillium brevicompactum SFC102216	MF186137.1			
					Penicillium brevicompactum 2-Z-30	MH310820.1			
**49**	65	SA	PDM	247	*Candida* sp. KJS-2016	LC155354.1	Saccharomycetales	99	MH881488
					Candida pseudolambica yHRM77	KM384061.1			
					Candida pseudolambica yHRM67	KM384060.1			
**50**	66	SE	PDM	182	*Fusarium lateritium* 3821	MG066631.1	Hypocreales	100	MH881489
					Fusarium tricinctum SFC101814	MF186086.1			
					Fusarium avenaceum SFC101774	MF186082.1			
**51**	67	FVE	WM	250	*Gibellulopsis nigrescens* STAF302	KU214559.1	Glomerellales	100	MH881490
					Gibellulopsis nigrescens LG1401 GL11A	KX359602.1			
					Gibellulopsis nigrescens STAF302	KU314961.1			
**52**	68	FVE	WM	316	*Penicillium atrovenetum* bqw5	MF599167.1	Eurotiales	100	MH881491
					Penicillium coralligerum YK-247	LC214562.1			
					Penicillium antarcticum SFC101809	MF186085.1			
**53**	69	FVE	PDM	329	*Penicillium brevicompactum* MERV5	MF503895.1	Eurotiales	100	MH881492
					Penicillium brevicompactum SFC102216	MF186137.1			
					Penicillium brevicompactum 2-Z-30	MH310820.1			
**54**	72	FVE	WM	204	*Penicillium* coralligerum YK-247	LC214562.1	Eurotiales	100	MH881493
					Penicillium atrovenetum bqw5	MF599167.1			
					Penicillium antarcticum SFC101809	MF186085.1			
**55**	73	FVE	GYP	321	*Penicillium brevicompactum* MERV5	MF503895.1	Eurotiales	98	MH881494
					Penicillium brevicompactum SFC102216	MF186137.1			
					Penicillium brevicompactum 2-Z-30	MH310820.1			

**56**	77	SE	PDM	249	*Penicillium* sp. L2-3C	MG813426.1	Eurotiales	100	MH881495
					Penicillium sp. UCD161305F	MG686509.1			
					Penicillium thomii MUT:2257	MG813165.1			
**57**	78	FVE	PDM	509	*Penicillium glabrum* DTO313-D4	MF803957.1	Eurotiales	100	MH881496
					Penicillium glabrum DTO313-C1	MF803952.1			
					Penicillium camemberti ISSFR-016	KT832784.1			
**58**	81	FVE	WM	227	*Wallemia muriae* KK18.3	KY322648.1	Wallemiales	100	MH881497
					Wallemia muriae KAS 6011	KX911859.1			
					Wallemia muriae KAS 5869	KX911850.1			
**59**	82	FVE	WM	328	*Emericellopsis terricola* CCF3815	J430737.1	Hypocreales	99	MH881498
					Emericellopsis minima OUCMBII111121	KT290876.1			
					Uncultured *Emericellopsis* 10J50C67 (MOTU75)	HG936806.1			
**60***	84	FVE	WM	323	*Stilbella fimetaria voucher* AF3-097G	KX446764.1	Hypocreales	100	MH881499
					Emericellopsis salmosynnemata CBS382.62	AY632666.1			
					Stilbella fimetaria D99026	AY952467.1			
**61**	85	SE	PDM	260	*Stereum hirsutum* P2A	KX838369.2	Russulales	100	MH881500
					Stereum hirsutum gap67	KX578081.1			
					Stereum hirsutum Wi-XI-1.1	MF476012.1			
**62**	86	FVE	PDM	323	*Emericellopsis terricola* CCF3815	FJ430737.1	Hypocreales	99	MH881501
					Emericellopsis terricola	U57676.1			
					Emericellopsis minima A11	KY775297.1			
**63***	87	FVE	WM	279	*Phoma* sp. MUT 5465	KU314987.1	Pleosporales	99	MH881502
					Pleosporales sp. AT60	KX953409.1			
					Pyrenochaetopsis microspora CBS 119739	LT623227.1			
**64**	89	FVE	WM	395	*Trichoderma paraviridescens*	MG646337.1	Hypocreales	100	MH881503
					Trichoderma viride IMF51186	LC317804.1			
					Trichoderma koningiopsis ACCC32904	MF871558.1			
**65**	91	FVE	GCM	473	*Penicillium spathulatum* AS3.15328	KC427190.1	Eurotiales	100	MH881504
					Penicillium spathulatum CBS 116977	JX313162.1			
					Penicillium sp. DF-2	KT121500.1			
**66**	92	FVS	PDM	330	*Penicillium coralligerum* YK-247	LC214562.1	Eurotiales	100	MH881505
					Penicillium sp. MT152	MH109377.1			
					Penicillium sp. MT57	MH109376.1			
**67**	95	FVE	PDM	350	*Penicillium coralligerum* YK-247	LC214562.1	Eurotiales	100	MH881506

					Penicillium sp. MT152	MH109377.1			
					Penicillium sp. MT57	MH109376.1			
**68**	96	FVS	GYP	502	*Penicillium brevicompactum* BEOFB1102m	MH630035.1	Eurotiales	100	MH881507
					Penicillium brevicompactum TCDFvLDB1814	MH714548.1			
					Penicillium brevicompactum 1	MH047201.1			
**69**	97	SE	GYP	428	*Fusarium culmorum* S68	MH681156.1	Eurotiales	100	MH88150
					Fusarium culmorum F150	MH681154.1			8
					Fusarium asiaticum G84	MH681153.1			
**70**	99	FVS	GCM	315	*Penicillium brevicompactum* KAS5854	KY469047.1	Eurotiales	100	MH881509
					Penicillium brevicompactum KAS5812	KY469041.1			
					Penicillium brevicompactum KAS5776	KY469037.1			
**71**	101	FVE	GCM	326	*Penicillium brevicompactum* KG_6i	MG686505.1	Eurotiales	100	MH8815010
					Penicillium brevicompactum Asa3SNA1	KY558614.1			
					Penicillium brevicompactum Cch1SNA3	KY558611.1			
**72**	102	FVE	W	349	*Penicillium* sp. 86	MF599167.1	Eurotiales	100	MH881511
					Penicillium atrovenetum BQW5	KY401137.1			
					Penicillium sp. 8	MF599167.1			
**73**	104	FVS	WM-S	318	*Penicillium chrysogenum* DCMAF01BCI	KY401130.1	Eurotiales	100	MH881512
					Penicillium brevicompactum SFC102216	MF186137.1			
					Penicillium brevicompactum 2-Z-30	MH310820.1			
**74**	105	FVS	GCM	377	*Penicillium* sp. 86	KY401137.1	Eurotiales	100	MH881513
					Penicillium atrovenetum BQW5	MF599167.1			
					Penicillium sp. 8	KY401130.1			
**75**	107	FVE	PDM	415	*Penicillium* sp. 86	KY401137.1	Eurotiales	100	MH881514
					Penicillium atrovenetum BQW5	MF599167.1			
					Penicillium sp. 8	KY401130.1			
**76**	108	FVS	WM	305	*Penicillium* sp. 86	KY401137.1	Eurotiales	100	MH881515
					Penicillium atrovenetum BQW5	MF599167.1			
					Penicillium sp. 8	KY401130.1			
**77**	109	FVS	GCM	291	*Penicillium* sp. 86	KY401137.1	Eurotiales	100	MH881516
					Penicillium atrovenetum BQW5	MF599167.1			
					Penicillium sp. 8	KY401130.1			
**78**	111	FVE	GYP	312	*Penicillium* sp. 86	KY401137.1	Eurotiales	100	MH881517
					Penicillium atrovenetum BQW5	MF599167.1			
					Penicillium sp. 8	KY401130.1			

**79**	112	FVE	GCM	367	*Penicillium glabrum* R2EK05	KR091814.1	Eurotiales	100	MH881518
					Penicillium glabrum SFC101259	MF185992.1			
					Penicillium glabrum SFC101229	MF185985.1			
**80***	113	SE	GCM	296	*Nectria inventa* MUT1135	KR709185.1	Hypocreales	100	MH881519
					Acrostalagmus luteoalbus P6424	MH063783.1			
					Acrostalagmus luteoalbus H35	KX375795.1			
**81**	114	FVS	PDM	300	*Penicillium brevicompactum* KAS5871	KY469055.1	Eurotiales	100	MH881520
					Penicillium brevicompactum SFC102216	MF186137.1			
					Penicillium brevicompactum 2-Z-30	MH310820.1			
**82**	115	SA	PDM	124	*Penicillium coralligerum* YK247	LC214562.1	Eurotiales	100	MH881521
					Penicillium atrovenetum bqw5	MF599167.1			
					Penicillium sp. 8	KY401130.1			
**83**	117	SE	PDM	434	*Phoma* sp. MUT 5465	KU314987.1	Pleosporales	100	MH881522
					Phoma sp. MUT 5462	KU314985.1			
					Phoma sp. MUT 5460	KU314983.1			
**84**	121	FVE	GCM	287	*Penicillium canescens* 3S.106	KY458474.1/	Eurotiales	100	MH881523
					Penicillium murcianum KK19.1	KY322566.1			
					Penicillium jensenii P6342	MH063657.1			
**85**	122	FVE	GCM	376	*Penicillium brevicompactum* KAS5871	KY469055.1	Eurotiales	100	MH881524
					Penicillium brevicompactum SFC102216	MF186137.1			
					Penicillium brevicompactum 2-Z-30	MH310820.1			
**86**	123	SE	GCM	102	*Penicillium* denovo13735	KR266650.1	Eurotiales	100	MH881525
					Penicillium sp. SF1	KX011018.1			
					Neosartorya sp. BAB-4715	KU571518.1			
**87**	124	SE	GCM	309	*Penicillium coralligerum* YK247	LC214562.1	Eurotiales	100	MH881526
					Penicillium atrovenetum bqw5	MF599167.1			
					Penicillium sp. 8	KY401130.1			

Table S2. Putatively identified compounds from 40 liquid culture extracts and 40 solid culture extracts. Dereplication of the compounds was based on GNPS, UNPD-ISDB, and manual dereplication by using several databases such as DNP, Scifinder and Chemspider.

Comp. No.	t_R (min)	Culture regime (medium)	Precursor *m/z*	Putative ID (chemical family)	Chemical structure	Molecular formula (change in Δ ppm)	MS/MS fragmentation	Strain number
**1**	0.70	Solid (SYM)	204.1113 $[M+H]^+$	Acetylcarnitine (amino acid derivative)	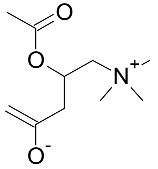	$C_9H_{17}NO_4$ (-59.3)*	145.1231; 85.9665	1
**2**	1.66	Liquid (WM)	189.1130 $[M+H]^+$	Aspinonene (pentaketide)	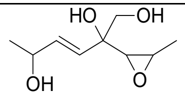	$C_9H_{16}O_4$ (0)	-	68
**3**	2.24	Solid (PDM, SYM, Cza, WM)	239.1592 $[M+H]^+$	Lysergine (indole alkaloid)	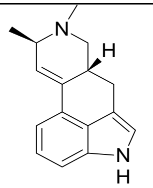	$C_{16}H_{18}N_2$ (-7.5)	197.1551; 141.9877	37
**4**	3.04	Solid (Cza)	245.1508 $[M+H]^+$	Cyclo(L-Phe-D-Pro) (diketopiperazine)	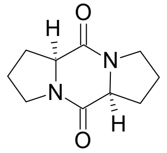	$C_{14}H_{16}N_2O_2$ (1.9)	217.1613; 154.1008; 120.1105	35
**5**	4.15	Liquid (PDM)	301.0852 $[M+H]^+$	Questinol (dihydroxyanthraquinone)	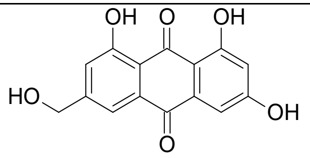	$C_{16}H_{12}O_6$ (-0.5)	286.0602; 257.0612	50

**6**	4.18	Solid (WM)	454.1482 $[M+Na]^+$	Pseurotin A (amide alkaloid)	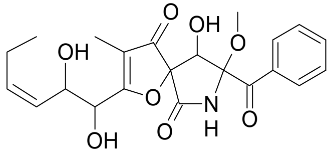	$C_{22}H_{25}NO_8$ (-0.9)	422.1124; 370.0889; 261.0882; 216.0832	78
		Liquid (SYM)						35
**7**	5.19	Liquid (Cza) Solid (Cza)	536.4123 $[M+H]^+$	2-(21-amino-3, 20-dihydroxydocosan-2-yl) oxy-6-(hydroxymethyl) oxane-3, 4, 5-triol (aminoglycolipid)	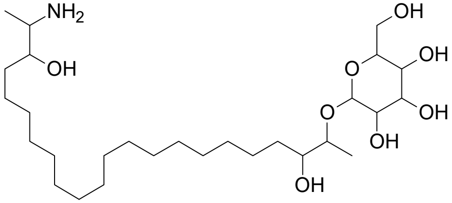	$C_{28}H_{57}NO_8$ (-1.9)	518.4053; 500.3958; 482.3873; 374.3875; 338.3654; 320.3527	59
**8**	5.19	Liquid (Cza) Solid (Cza)	356.3711 $[M+H]^+$	21-aminodocos-21-ene-2,3-diol (aminolipid)	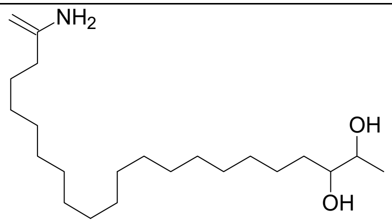	$C_{22}H_{45}NO_2$ (0.5)	338.3616; 320.3527	59
**9**	5.20	Liquid (Cza) Solid (Cza)	374.3796 $[M+H]^+$	21-aminodocosane-2,3,20-triol (aminolipid)	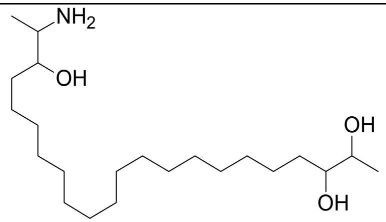	$C_{22}H_{47}NO_3$ (42.2) *	356.3704; 338.3615; 320.3528	59

**10**	5.21	Liquid (Cza)<br>Solid (Cza)	500.3968 $[M+H]^+$	(*E*)-2-((21-aminodocosa-3,21-dien-2-yl)oxy)-6-(hydroxymethyl)tetrahydro-2*H*-pyran-3, 4, 5-triol<br>(aminolipid)	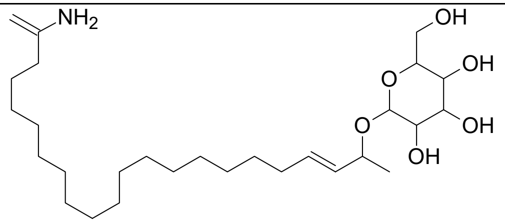	$C_{28}H_{53}NO_6$<br>(3.2)	482.3876;<br>374.3875;<br>356.3695;<br>338.3606;<br>320.3527	59
**11**	5.23	Liquid (Cza)<br>Solid (Cza)	518.4051 $[M+H]^+$	2-(21-amino-3-hydroxydocos-21-en-2-yl) oxy)-6-(hydroxymethyl) tetrahydro-2*H*-pyran-3, 4, 5-triol<br>(aminoglycolipid)	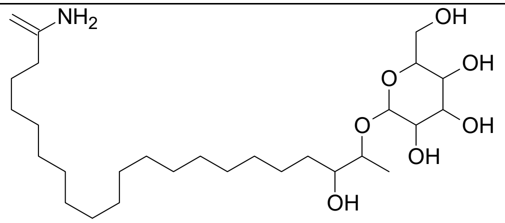	$C_{28}H_{55}NO_7$<br>(-1.7)	500.3958;<br>482.3873;<br>356.3700<br>338.3612;<br>320.3526	59
**12**	5.47	Solid (WM)	466.3170 $[M+H]^+$<br>488.2957 $[M+Na]^+$	Glycocholic acid<br>(bile acid)	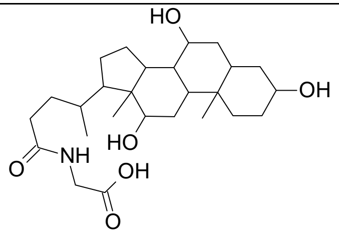	$C_{26}H_{44}NO_6$<br>(0.2)	412.2906;<br>337.2691;<br>319.2593;<br>227.1693<br>470.2871;<br>412.2914	59
**13**	6.32	Liquid (Cza, SYM)<br>Solid (SYM, WM, PDM)	501.2903 $[M+H]^+$	Citreohybridonol<br>(sesterterpenoid)	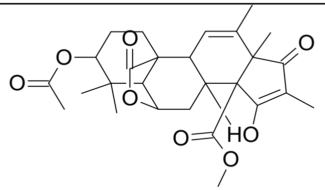	$C_{28}H_{36}O_8$<br>(-0.3)	486.2682;<br>409.2138;<br>381.2216;<br>363.2131;<br>335.2205;<br>217.1520;<br>179.1021	68

**14**	6.45	Solid (SYM)	318.3213 [M+H]+	Phytosphingosine (sphingolipid)	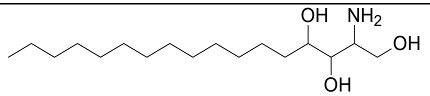	$C_{18}H_{39}NO_3$ (6.5)	300.3127; 282.3036; 270.3043; 264.2945; 240.2946;	56
**15**	6.52	Liquid (WM)	353.0795 [M+H]+	Griseofulvin (tetrahydrofuranone polyketide)	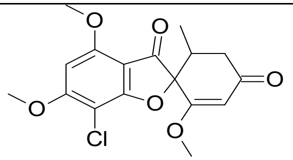	$C_{17}H_{17}ClO_6$ (0.8)	285.0648; 215.0305; 165.0792	68
**16**	6.70	Liquid (Cza) Solid (WM)	454.1801 [M+Na]+	Fusarin C (polyene)	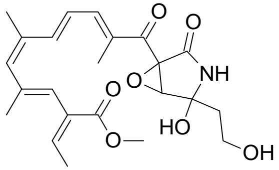	$C_{23}H_{29}NO_7$ (-10.4)	413.2605; 335.1281; 290.1098	58
**17**	6.78	Liquid (Cza, PDM)	519.1832 [M+H]+	Rubratoxin B (bis-anhydride)	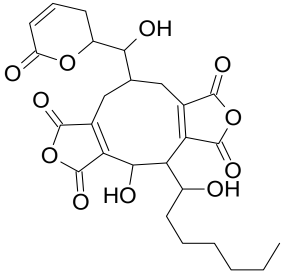	$C_{26}H_{30}O_{11}$ (-4.0)	501.1757; 451.1306; 271.1122	50

**18**	6.99	Liquid (Cza, PDM)	521.1890 [M+H]+	Rubratoxin A (bis-anhydride)	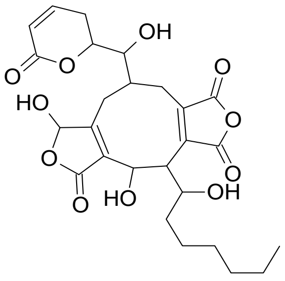	$C_{26}H_{32}O_{11}$ (-5.0)	503.1911; 435.1422; 283.1126; 271.1121	50
**19**	7.14	Liquid (PDM)	589.0890 [M+H-H2O]+	Xanthoepocin (binaphthoquinone)	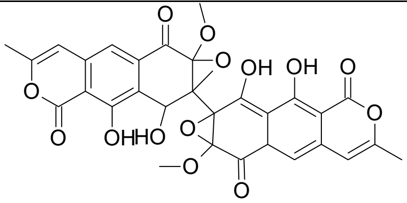	$C_{30}H_{22}O_{14}$ (-10.6)	571.0801; 483.0744; 470.0672; 441.0686; 413.0764; 313.0562	59
**20**	7.16	Liquid (PDM)	571.0803 [M+H]+	Bisdehydroxanthomegn in (binaphthoquinone)	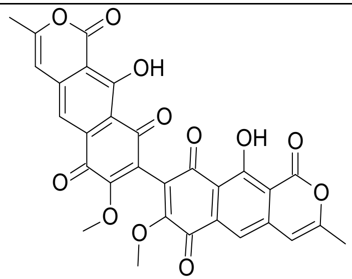	$C_{30}H_{18}O_{12}$ (-12.6)	511.0682; 483.0735; 469.0599; 286.0680; 257.0700	59
**21**	7.46	Liquid (SYM, PDM, Cza)	343.1276 [M+H]+	Atrovenetin (aromatic polyketide)	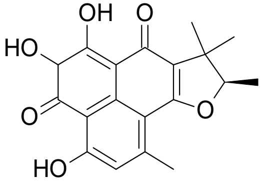	$C_{19}H_{18}O_6$ (24.30)	287.0714; 255.0495	68

**22**	7.55	Solid (SYM, Cza)	337.2900 $[M+H-H_2O]^+$	Prostaglandin H_1 (fatty acid derivative)	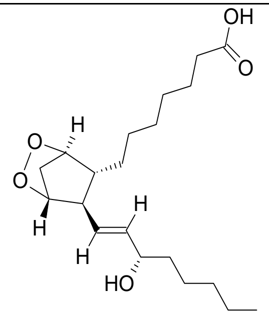	$C_{20}H_{34}O_5$ (8.6)	263.2607; 123.1482; 109.1327	78
**23**	7.58	Solid (WM, Cza)	513.2470 $[M+H]^+$	Arisugacin D (dihydroxanthone derivative)	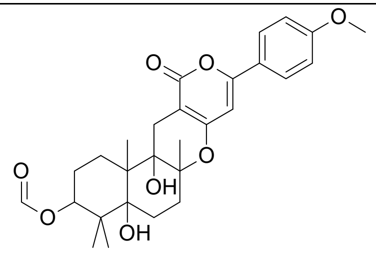	$C_{29}H_{36}O_8$ (-3.9)	495.2372; 395.1965; 209.1114	50
**24**	7.67	Solid (SYM, Cza)	446.2421 $[M+Na]^+$	Brevicompanine E (diketopiperazine alkaloid)	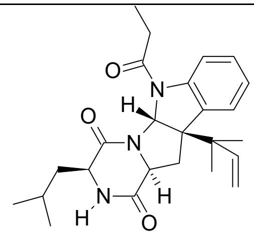	$C_{25}H_{33}N_3O_3$ (-0.4)	386.2237; 328.1892; 130.0972; 358.1982	68
**25**	8.05	Solid (WM, SYM, PDM, Cza)	357.1544 $[M+H]^+$	Deoxyherquenone (aromatic polyketide)	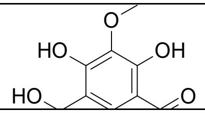	$C_{20}H_{20}O_6$ (5.4)	342.1268; 327.1049; 301.0910	50

		Liquid (SYM, PDM, Cza, WM)						68
**26**	8.12	Liquid (SYM)	481.2016 [M+Na]+	Fumagillin (meroterpenoid)	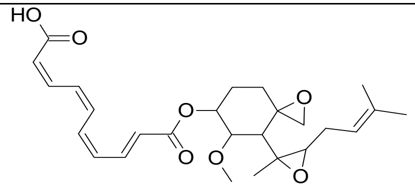	$C_{26}H_{34}O_7$ (-3.8)	329.2926	35
**27**	8.88	Solid (SYM)	448.2588 [M+H]+	Marcfortine C (indole alkaloid)	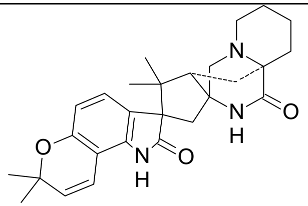	$C_{27}H_{33}N_3O_3$ (-2.7)	406.2837; 330.2077; 182.1254	68
**28**	9.16	Liquid (SYM)	379.3 329 [M+H-H2O]+	Ergosterol (steroid)	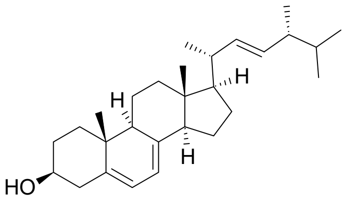	$C_{28}H_{44}O$ (-9.3)	309.2665; 295.2521; 253.2106; 213.1840; 199.1696; 159.1414	35
**29**	9.35	Liquid (WM, Cza, PDM)	341.1123 [M+H]+	Atrovenetinone (aromatic polyketide)	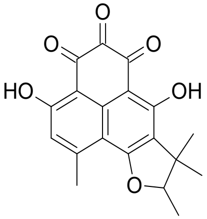	$C_{19}H_{16}O_6$ (27.9)	313.7212; 287.0724	68

**30**	9.78	Solid (SYM)	584.3291 $[M+H]^+$	Penitrem B (indole diterpenoid)	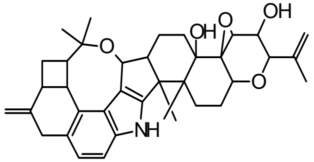	$C_{37}H_{46}NO_5$ (-14.2)	566.3217; 514.2602; 497.2591; 480.2563; 478.2471 304.1931; 260.1345	68
**31**	10.16	Liquid (PDM, Cza)	640.3732 $[M+H]^+$	Enniatin B (cyclic depsipeptide)	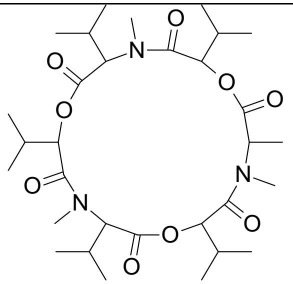	$C_{33}H_{57}N_3O_9$ (-53.4) *	527.3075; 427.2713; 314.2042; 214.1643; 195.1562; 186.1716	35
		Liquid (PDM, Cza)	662.3557 $[M+Na]^+$				549.2864; 336.1829	
**32**	10.30	Solid (Cza)	481.2922 $[M+Na]^+$	Candelalide C (diterpenoid pyrone)	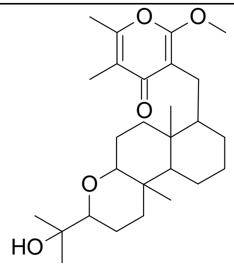	$C_{28}H_{42}O_5$ (-1.7)	355.2387; 283.2012; 240.2581; 133.1116	50
**33**	10.61	Liquid (SYM)	331.2302 $[M+H]^+$	Phomopsidin (trimethylated nonaketide)	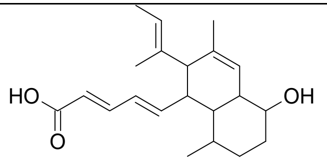	$C_{21}H_{30}O_3$ (6.5)	293.0333; 273.8689; 250.8805′ 192.8811; 165.0659	87

**34**	10.66	Liquid (WM, PDM)	654.3968 [M+H]$^+$	Enniatin B1 (cyclic depsipeptide)	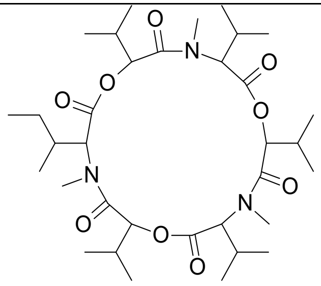	$C_{34}H_{59}N_3O_9$ (-55.3) *	541.3203; 441.2848; 328.2178; 314.2042; 210.1696; 196.1553	35
		Liquid (WM, PDM, Cza, SYM)	676.3968 [M+Na]$^+$				563.2997	
**35**	10.95	Liquid (PDM, Cza, WM)	463.2682 [M+H]$^+$	Virescenoside E (diterpene glycoside)	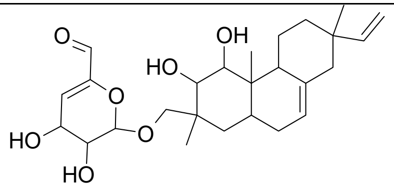	$C_{26}H_{38}O_7$ (-5.2)	355.2312	1
**36**	11.67	Liquid (PDM, WM)	704.3914 [M+Na]$^+$	Enniatin A (cyclic depsipeptide)	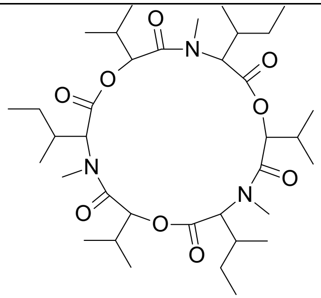	$C_{36}H_{63}N_3O_9$ (48.5) *	577.3101	35

*Peak ions of low intensity (molecular formula prediction with high Δ ppm.

Supplementary Figures

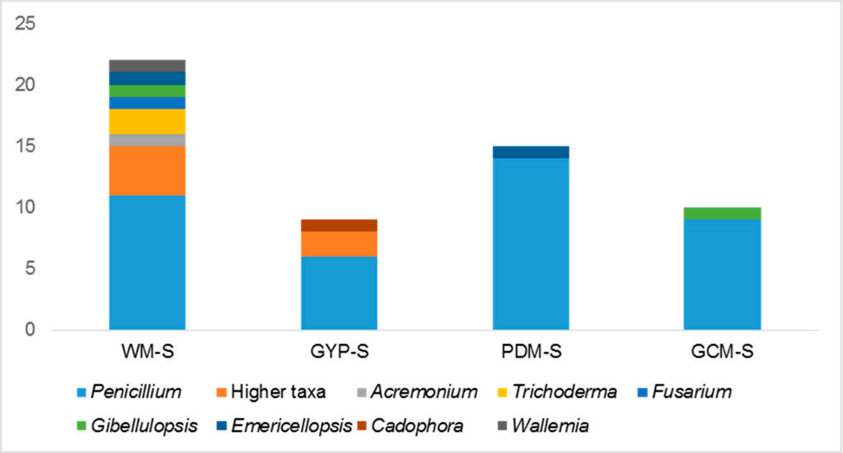

Figure S1. Comparison of 55 *F. vesiculosus* – derived fungi in diversity and distribution according to solid (S) isolation media. WM-S = modified Wickerham medium, PDM-S = Potato Dextrose medium, GCM-S = Glucose Casein medium, GYP-S = Glucose Yeast Peptone medium. Height of the column represents the total number of isolates derived from each media – WM-S: 21 isolates; PDM-S: 15 isolates, GCM-S: 10 isolates. GYP-S: 9 isolates.

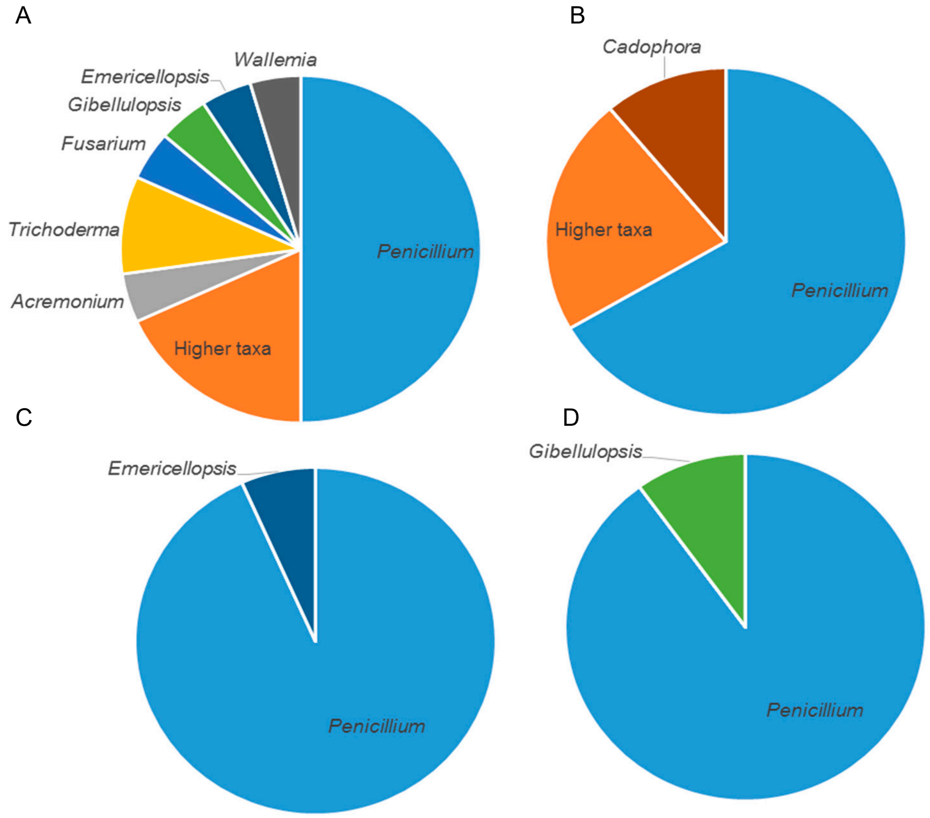

Figure S2. Pie chart showing the diversity of 55 *Fucus vesiculosus* – derived fungal community obtained by different isolation media. Strains that could not be identified to genus level are indicated as 'Higher taxa'. **(A).** Fungal isolates obtained from the WM-S medium that were identified as 10 *Penicillium* sp., 1 *Emericellopsis* sp., 1 *Gibellulopsis* sp., 1 *Fusarium* sp., 2 *Trichoderma* sp., 1 *Acremonium* sp., 1 *Wallemia* sp. and 4 'Higher taxa'. **(B).** Fungal isolates obtained from the GYP-S medium that were identified as 6 *Penicillium* sp., 2 'Higher taxa', and 1 *Cadophora* sp. **(C).** Fungal

isolates obtained from the PDM-S medium that were identified as 14 *Penicillium* sp. and 1 *Emericelosis* sp. **(D).** Fungal isolates obtained from the GCM-S medium that were identified as 9 *Penicillium* sp. and 1 *Gibellulopsis* sp.

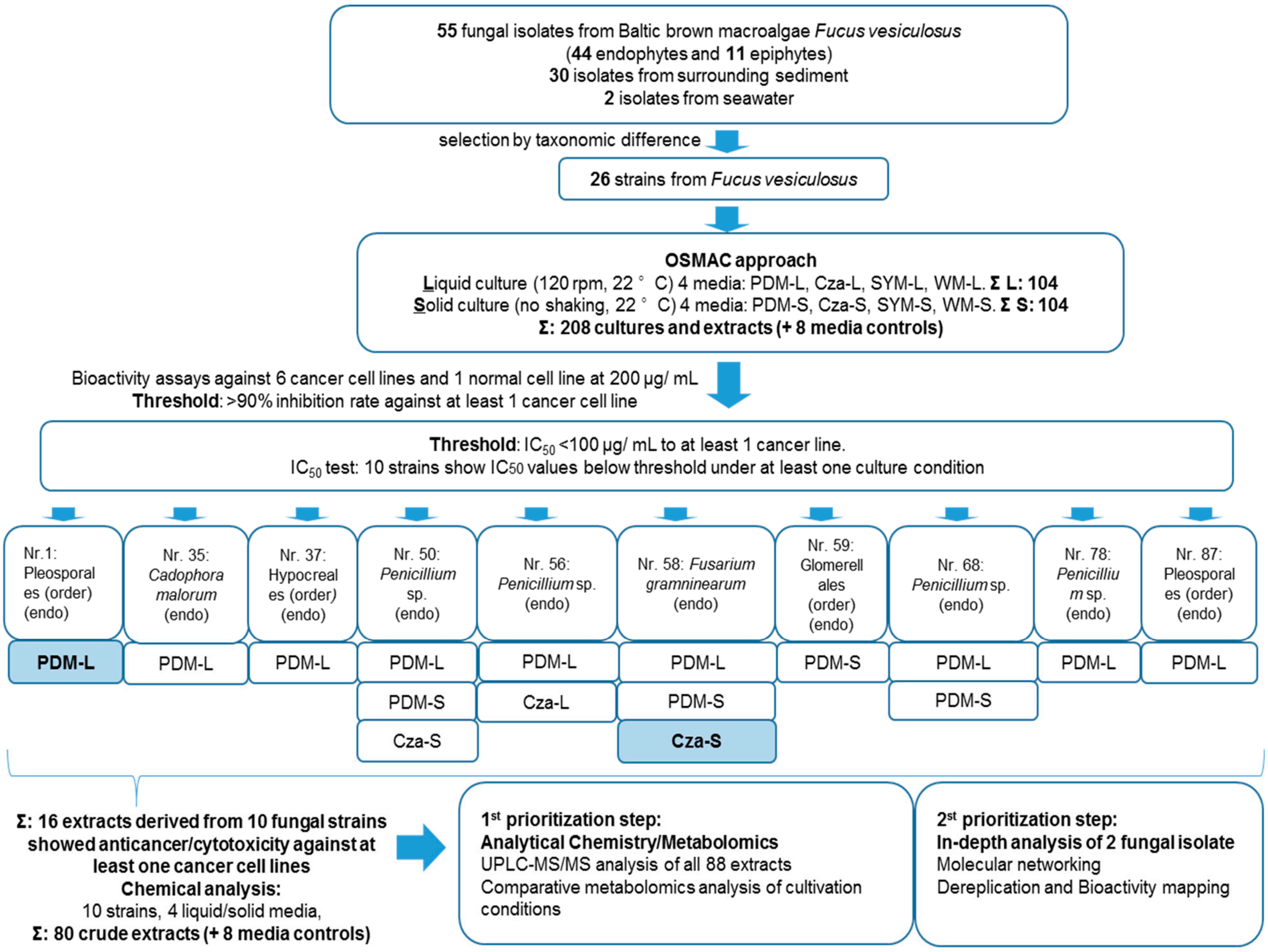

Figure S3. Workflow for the OSMAC study based on the 55 fungal isolates derived from *F. vesiculosus*. 26 isolates were selected for small-scale fermentation. The OSMAC approach was designed using 4 different media and 2 culture regimes. In total 208 culture extracts were tested for anticancer activity and the bioactive samples were further tested for IC_{50} determination. 10 strains showed IC_{50} values below 100 µg/mL against at least one cancer cell line. In the end, all 80 culture extracts derived from 10 strains were analysed by an integrated metabolomics approach.

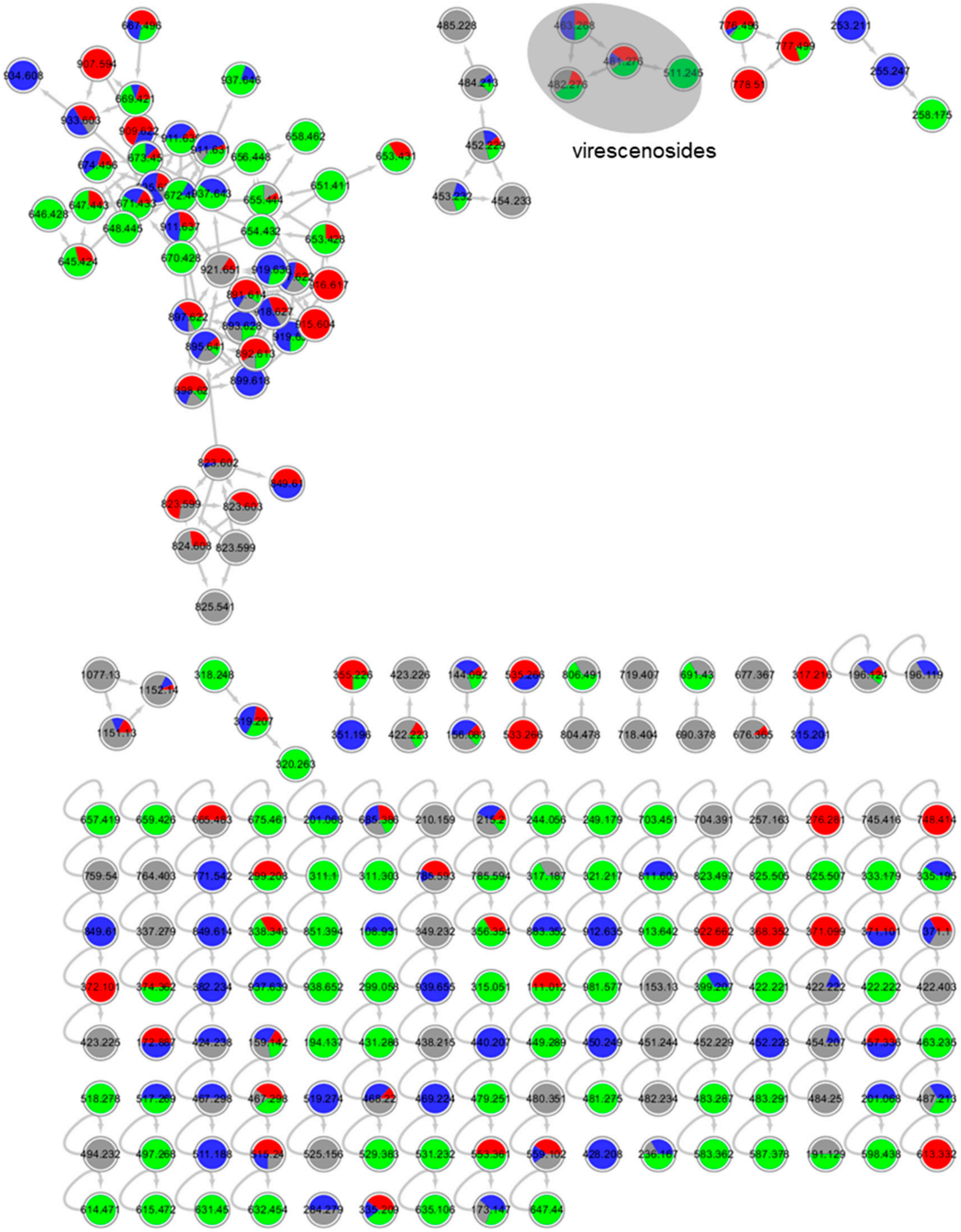

Figure S4. Molecular network for strain 1 (order Pleosporales) liquid culture extracts in PDM-L (**blue**), Cza-L (**red**), SYM-L (**green**), WM-L (**grey**). Annotated peak ions of putatively known compounds are highlighted by a grey loop.

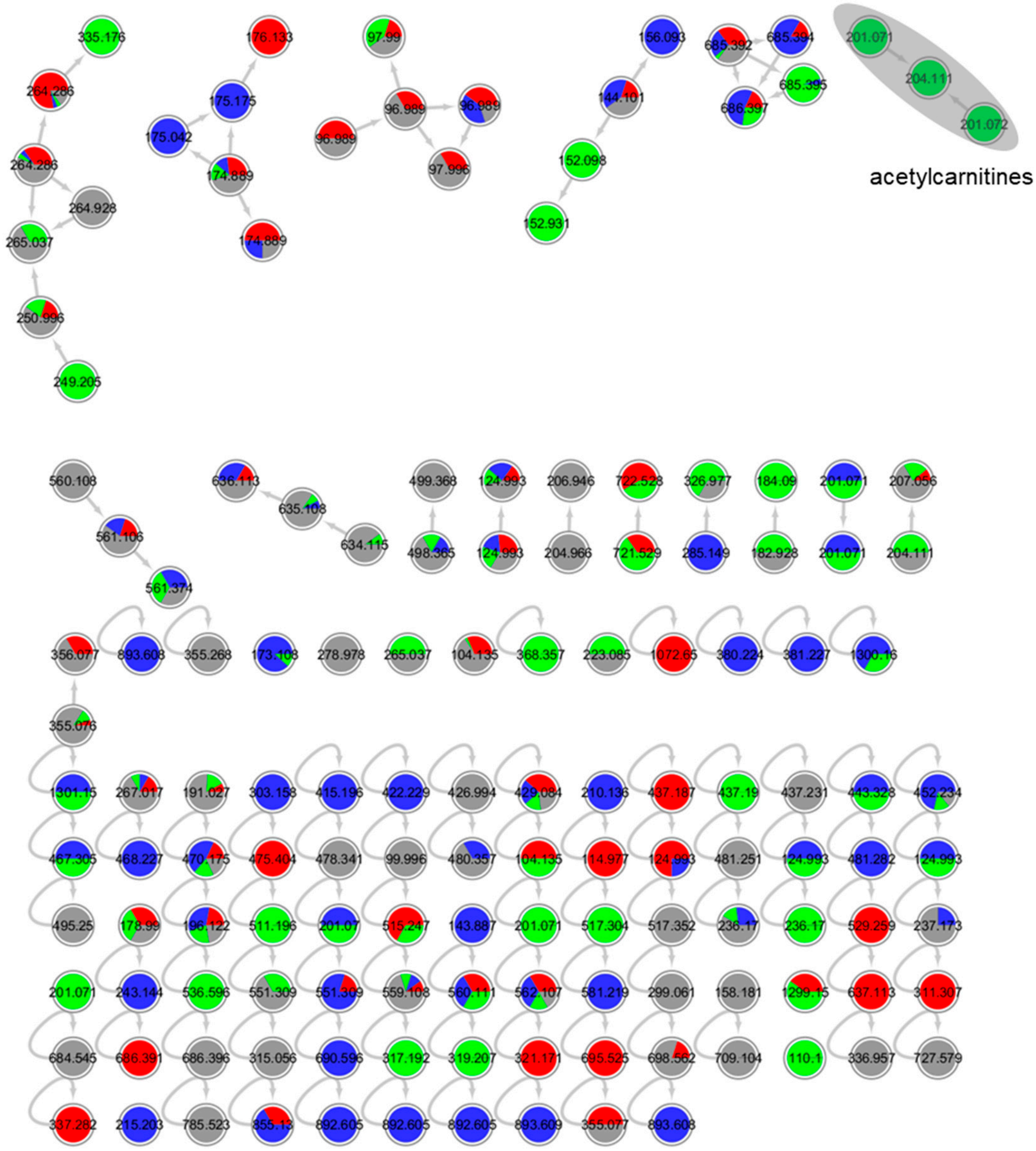

Figure S5. Annotated molecular network for strain 1 (order Pleosporales) solid culture extracts in PDM-S (**blue**), Cza-S (**red**), SYM-S (**green**), WM-S (**grey**). Annotated peak ions of putatively known compounds are highlighted by a grey loop.

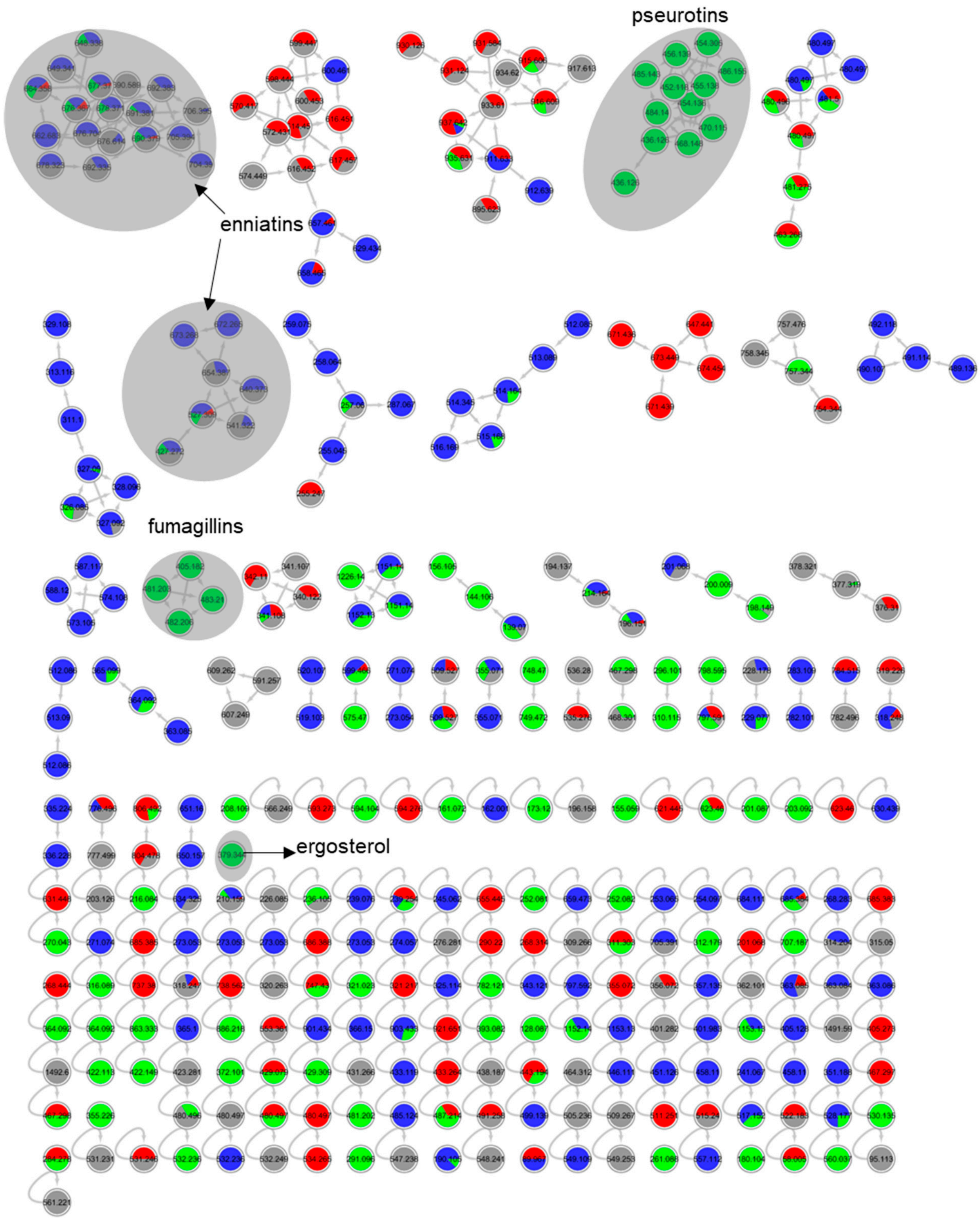

Figure S6. Annotated molecular network for strain 35 (*Cadophora malorum*) liquid culture extracts in PDM-L (**blue**), Cza-L (**red**), SYM-L (**green**), WM-L (**grey**). Annotated peak ions of putatively known compounds are highlighted by a grey loop.

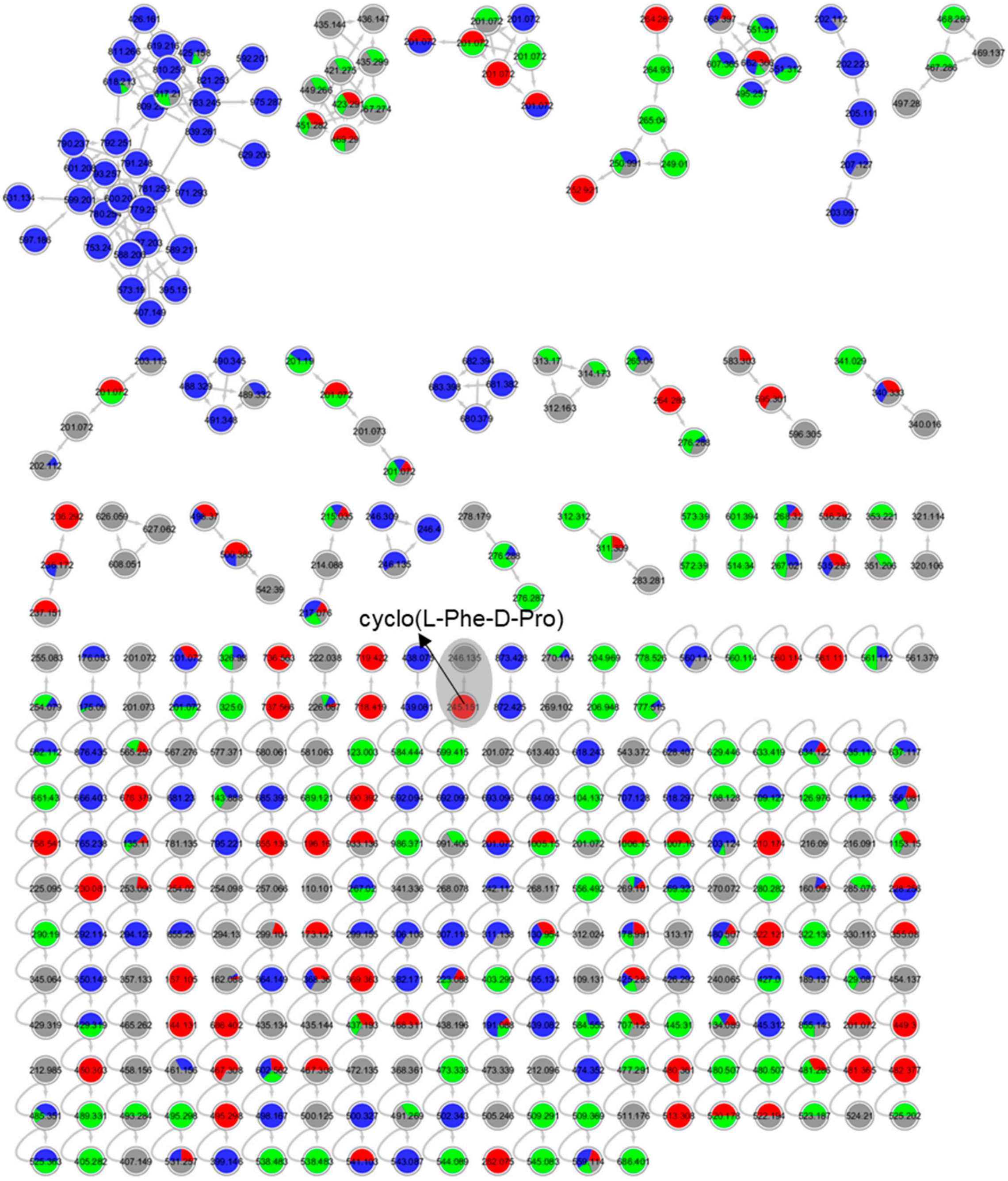

Figure S7. Annotated molecular network for strain 35 (*Cadophora malorum*) solid culture extracts in PDM-S (**blue**), Cza-S (**red**), SYM-S (**green**), WM-S (**grey**). Annotated peak ions of putatively known compounds are highlighted by a grey loop.

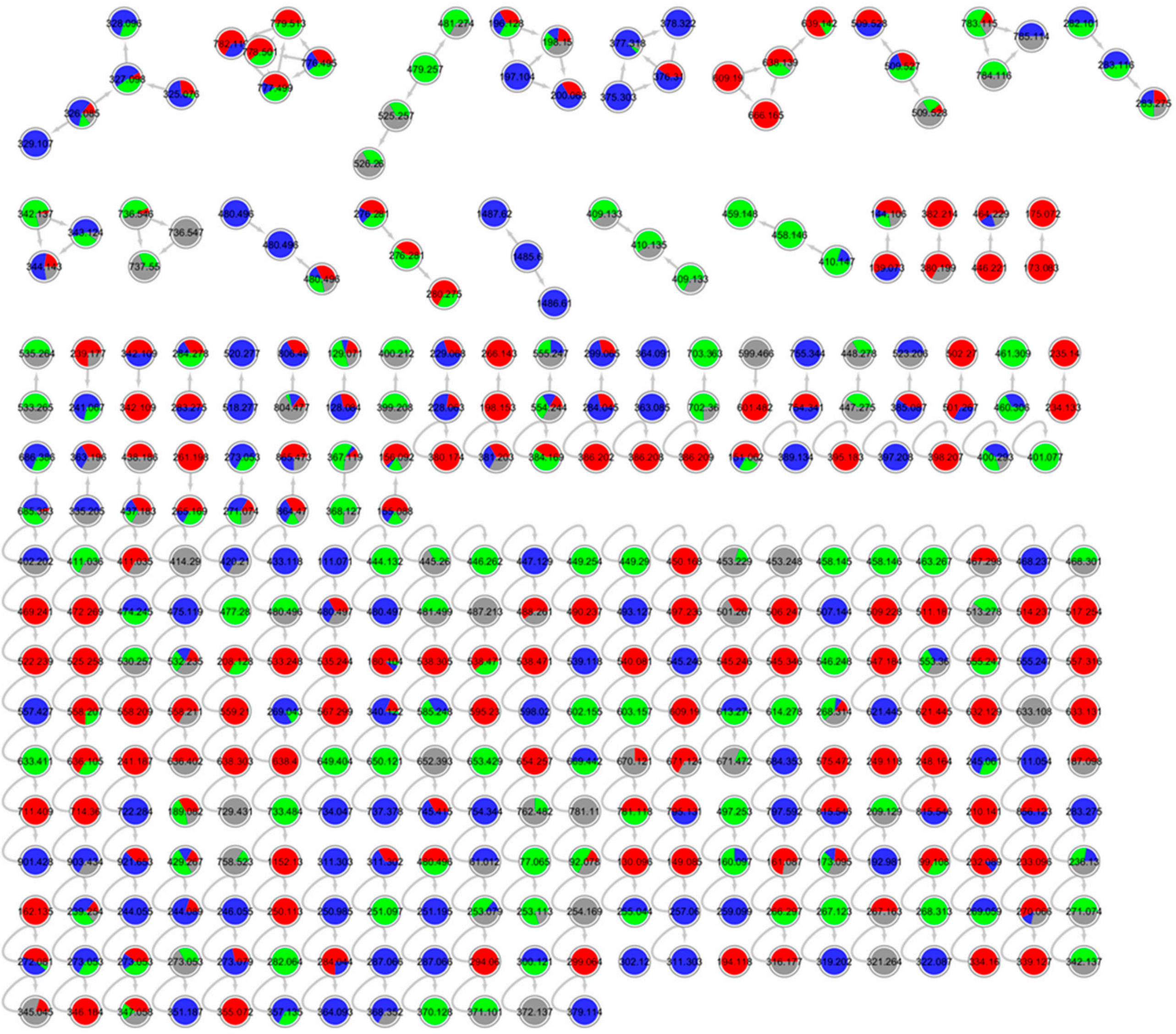

Figure S8. Annotated molecular network for strain 37 (order Hypocreales) liquid culture extracts in PDM-L (**blue**), Cza-L (**red**), SYM-L (**green**), WM-L (**grey**).

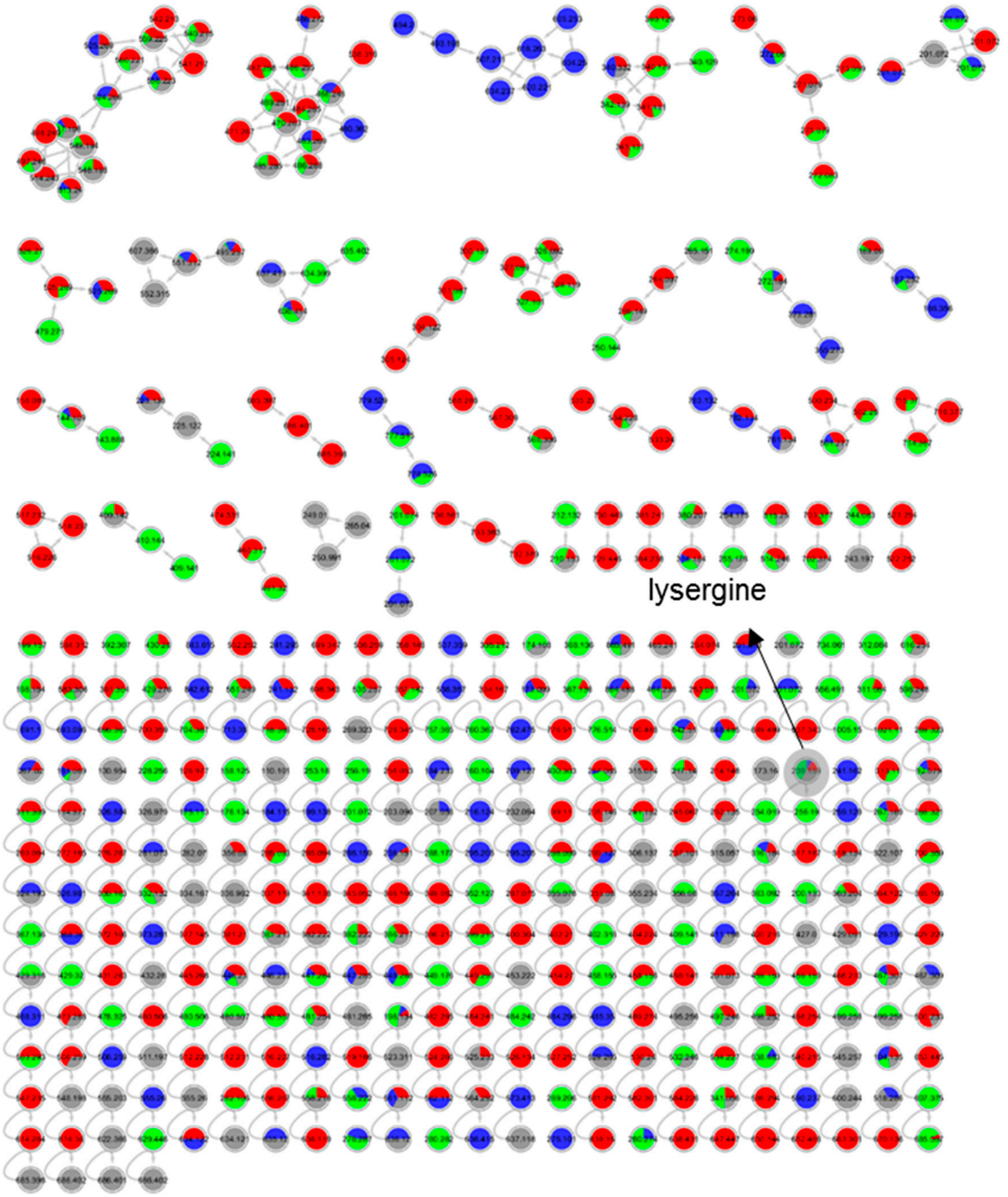

Figure S9. Annotated molecular network for strain 37 (order Hypocreales) solid culture extracts in PDM-S (**blue**), Cza-S (**red**), SYM-S (**green**), WM-S (**grey**). Annotated peak ions of putatively known compounds are highlighted by a grey loop.

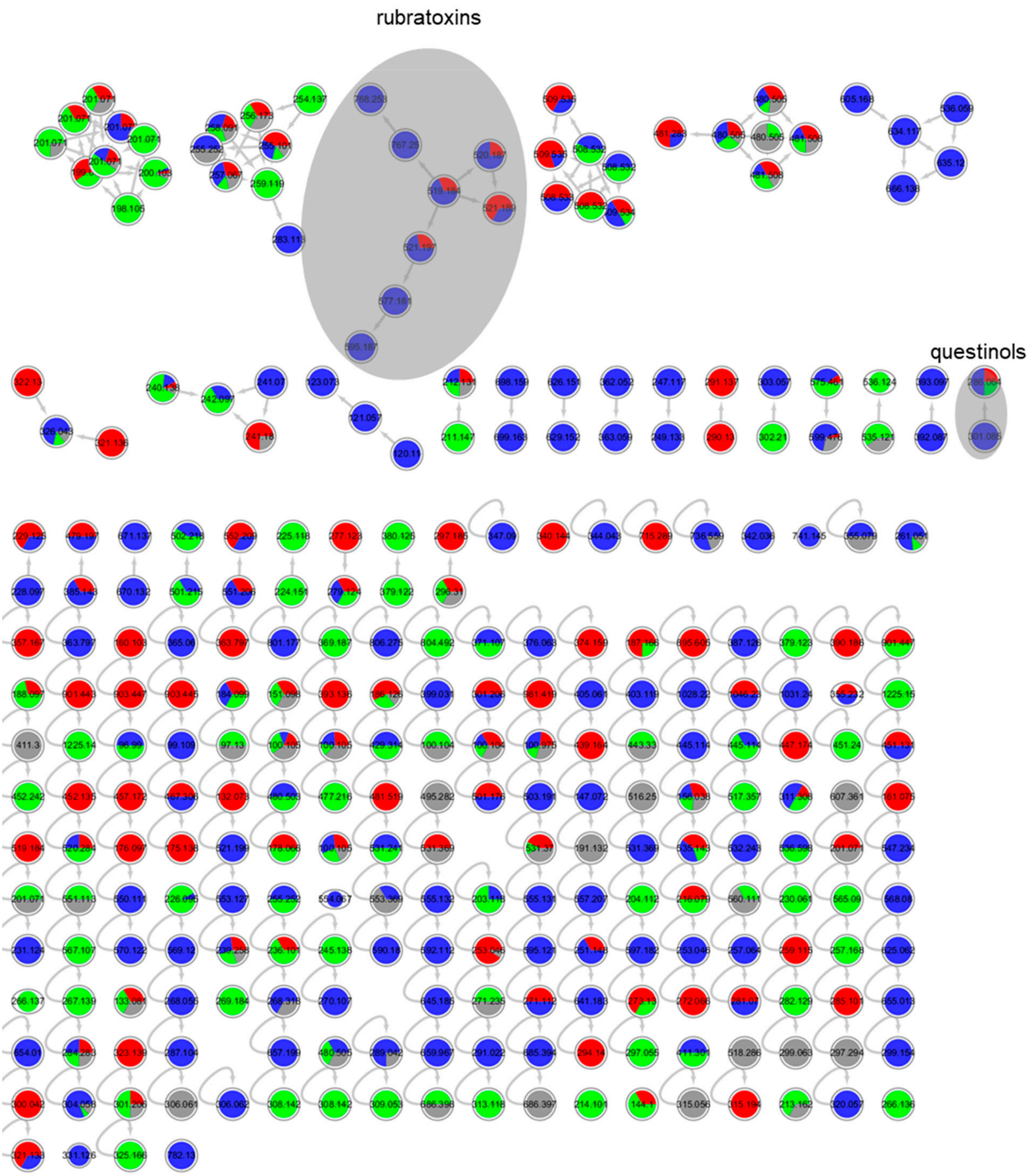

Figure S10. Annotated molecular network for strain 50 (*Penicillium* sp.) liquid culture extracts in PDM-L (**blue**), Cza-L (**red**), SYM-L (**green**), WM-L (**grey**). Annotated peak ions of putatively known compounds are highlighted by a grey loop.

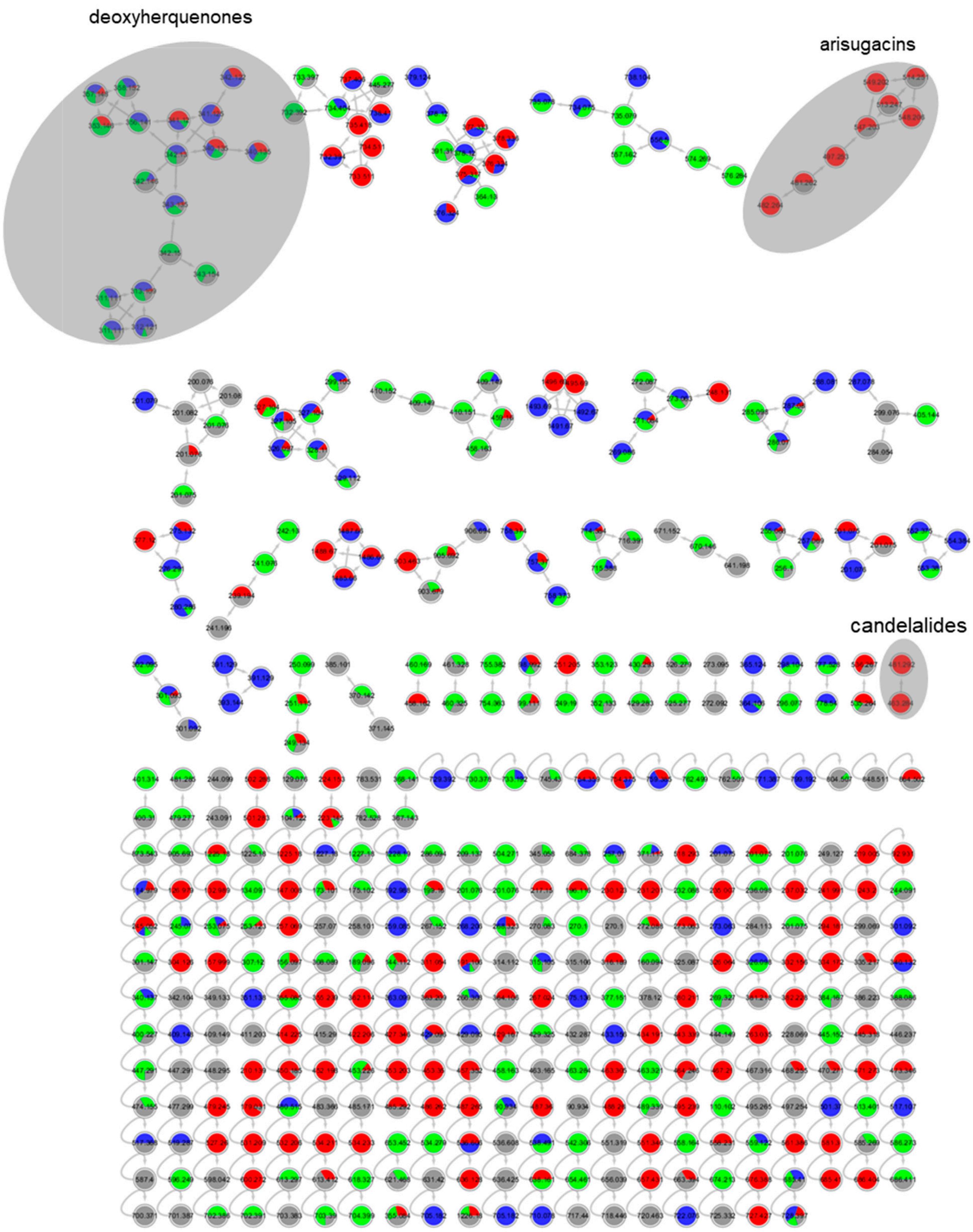

Figure S11. Annotated molecular network for strain 50 (*Penicillium* sp.) solid culture extracts in PDM-S (**blue**), Cza-S (**red**), SYM-S (**green**), WM-S (**grey**). Annotated peak ions of putatively known compounds are highlighted by a grey loop.

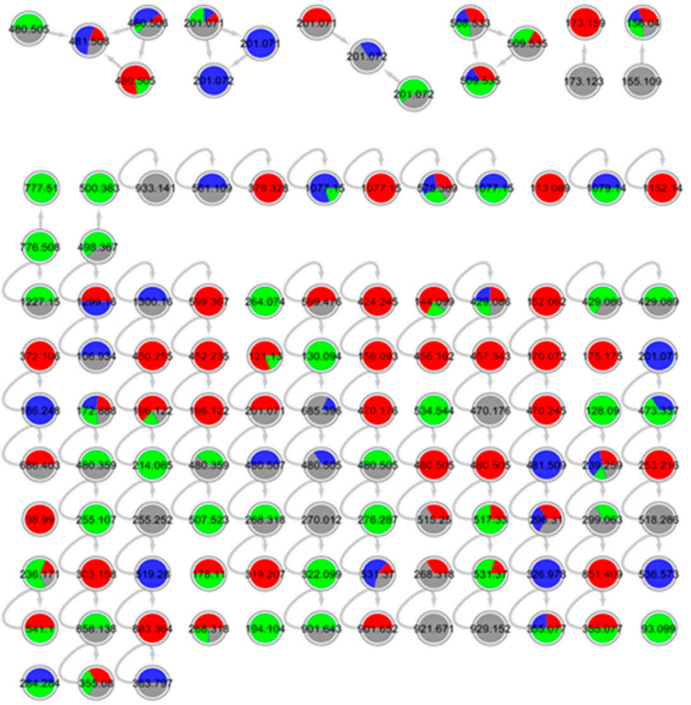

Figure S12. Annotated molecular network for strain 56 (*Penicillium* sp.) liquid culture extracts in PDM-L (**blue**), Cza-L (**red**), SYM-L (**green**), WM-L (**grey**).

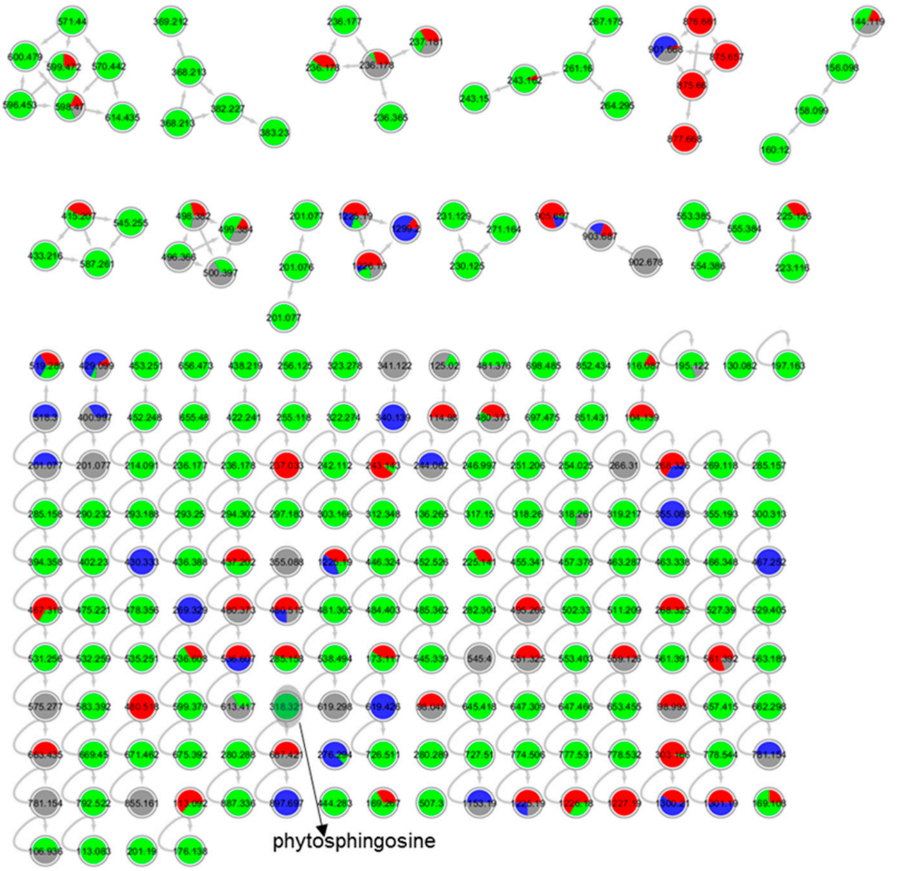

Figure S13. Annotated molecular network for strain 56 (*Penicillium* sp.) solid culture extracts in PDM-S (**blue**), Cza-S (**red**), SYM-S (**green**), WM-S (**grey**). Annotated peak ions of putatively known compounds are highlighted by a grey loop.

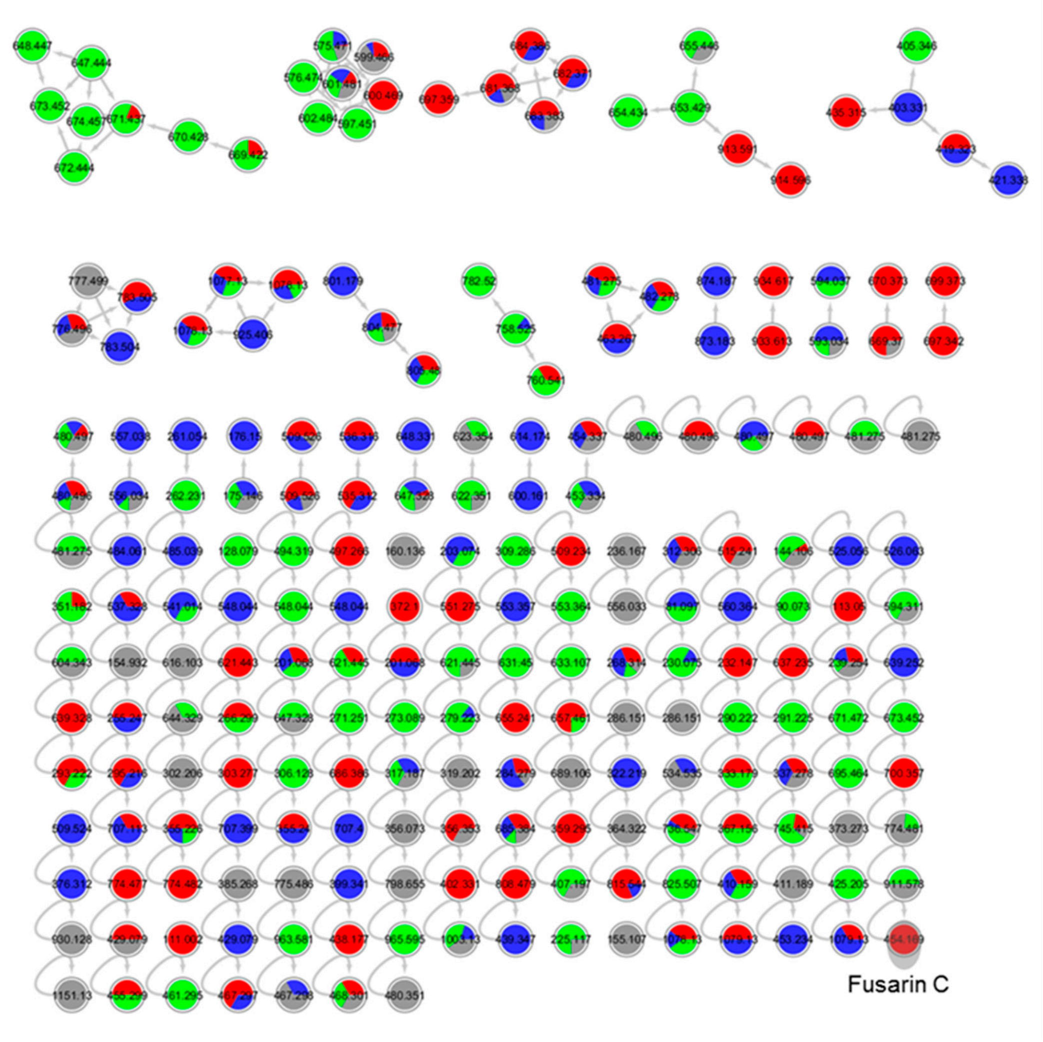

Figure S14. Annotated molecular network for strain 58 (*Fusarium graminearum*) liquid culture extracts in PDM-L (**blue**), Cza-L (**red**), SYM-L (**green**), WM-L (**grey**). Annotated peak ions of putatively known compounds are highlighted by a grey loop.

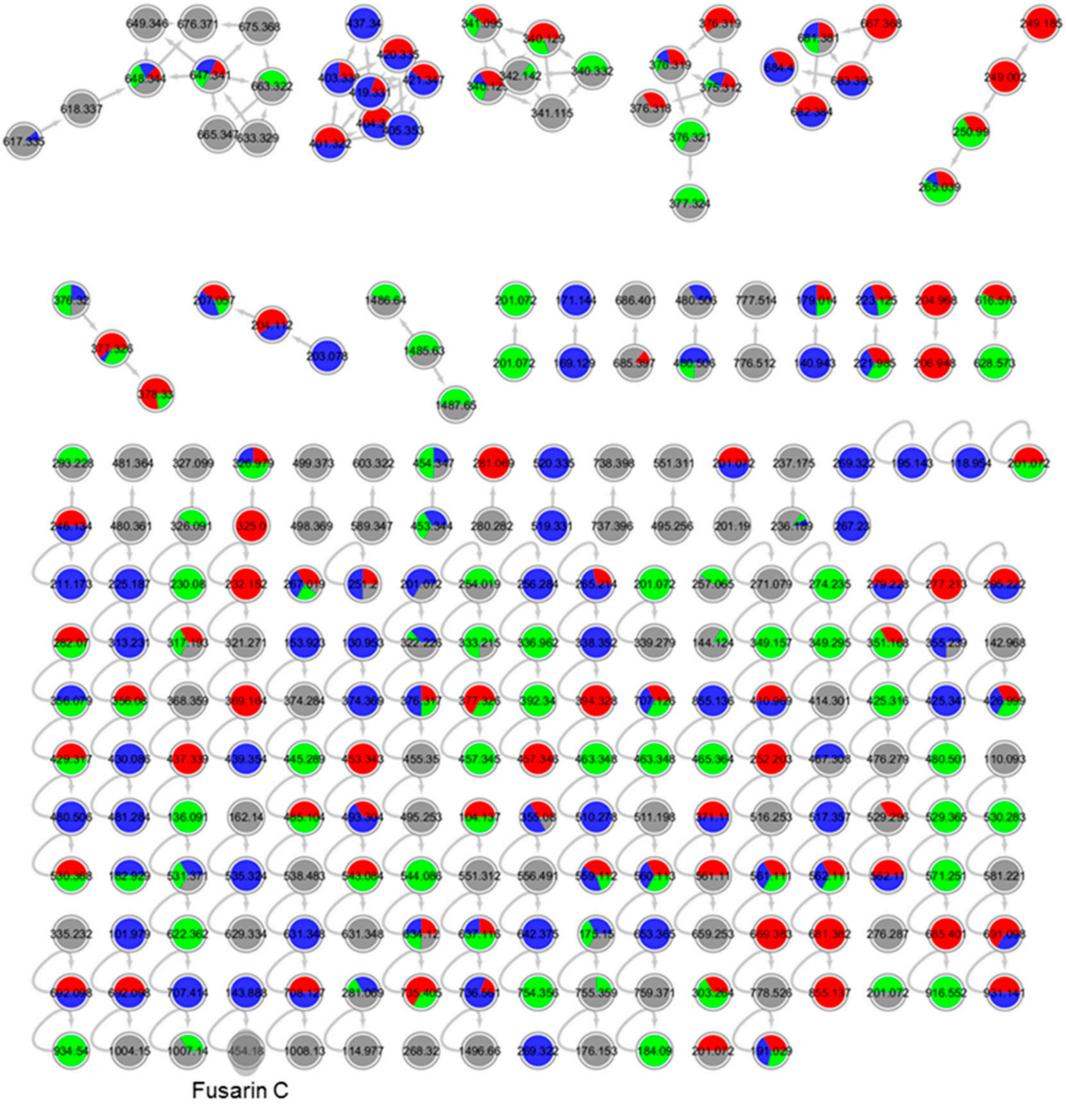

Figure S15. Annotated molecular network for strain 58 (*Fusarium graminearum*) solid culture extracts in PDM-S (**blue**), Cza-S (**red**), SYM-S (**green**), WM-S (**grey**). Annotated peak ions of putatively known compounds are highlighted by a grey loop.

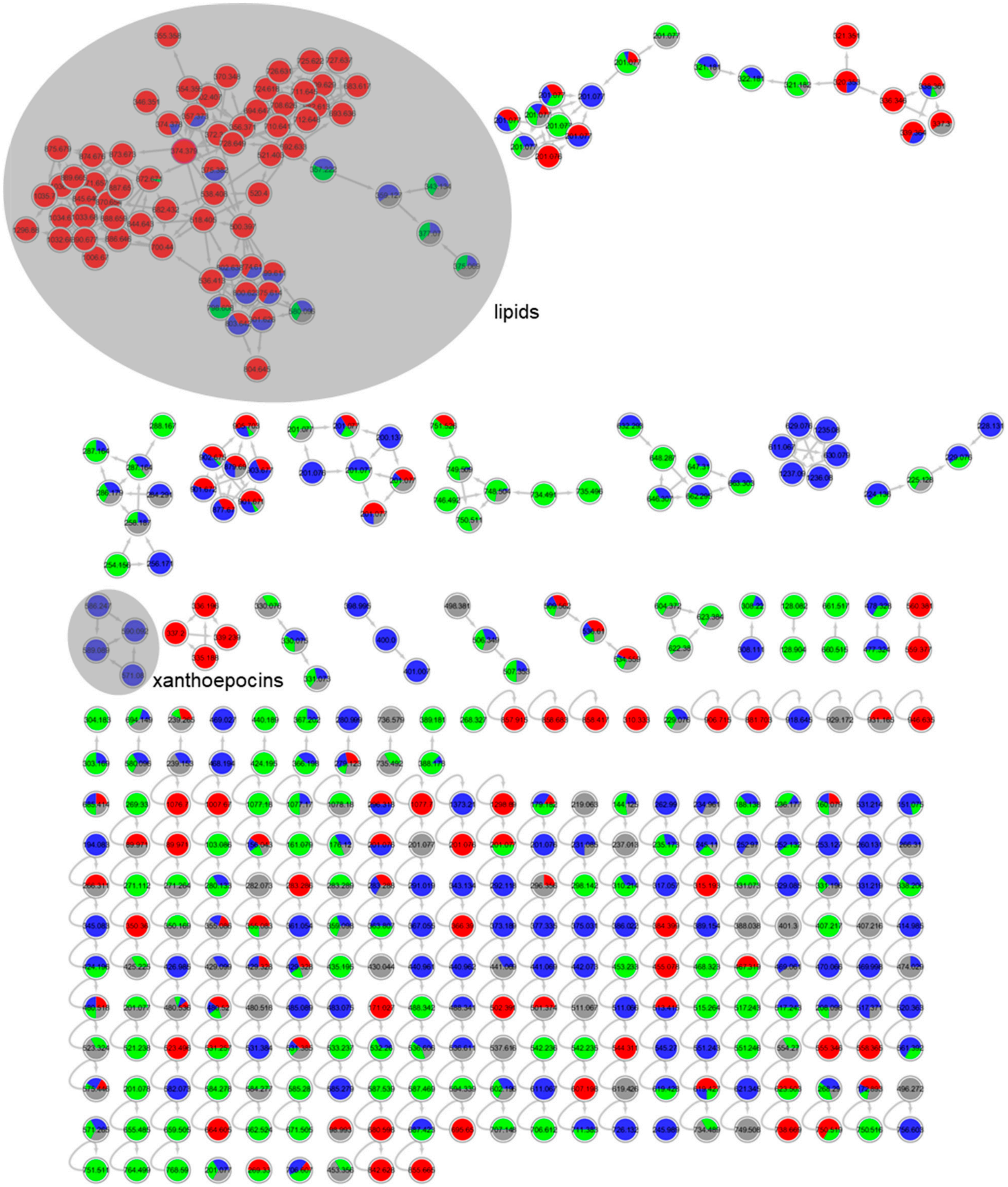

Figure S16. Annotated molecular network for strain 59 (order Glomerellales) liquid culture extracts in PDM-L (**blue**), Cza-L (**red**), SYM-L (**green**), WM-L (**grey**). Annotated peak ions of putatively known compounds are highlighted by a grey loop.

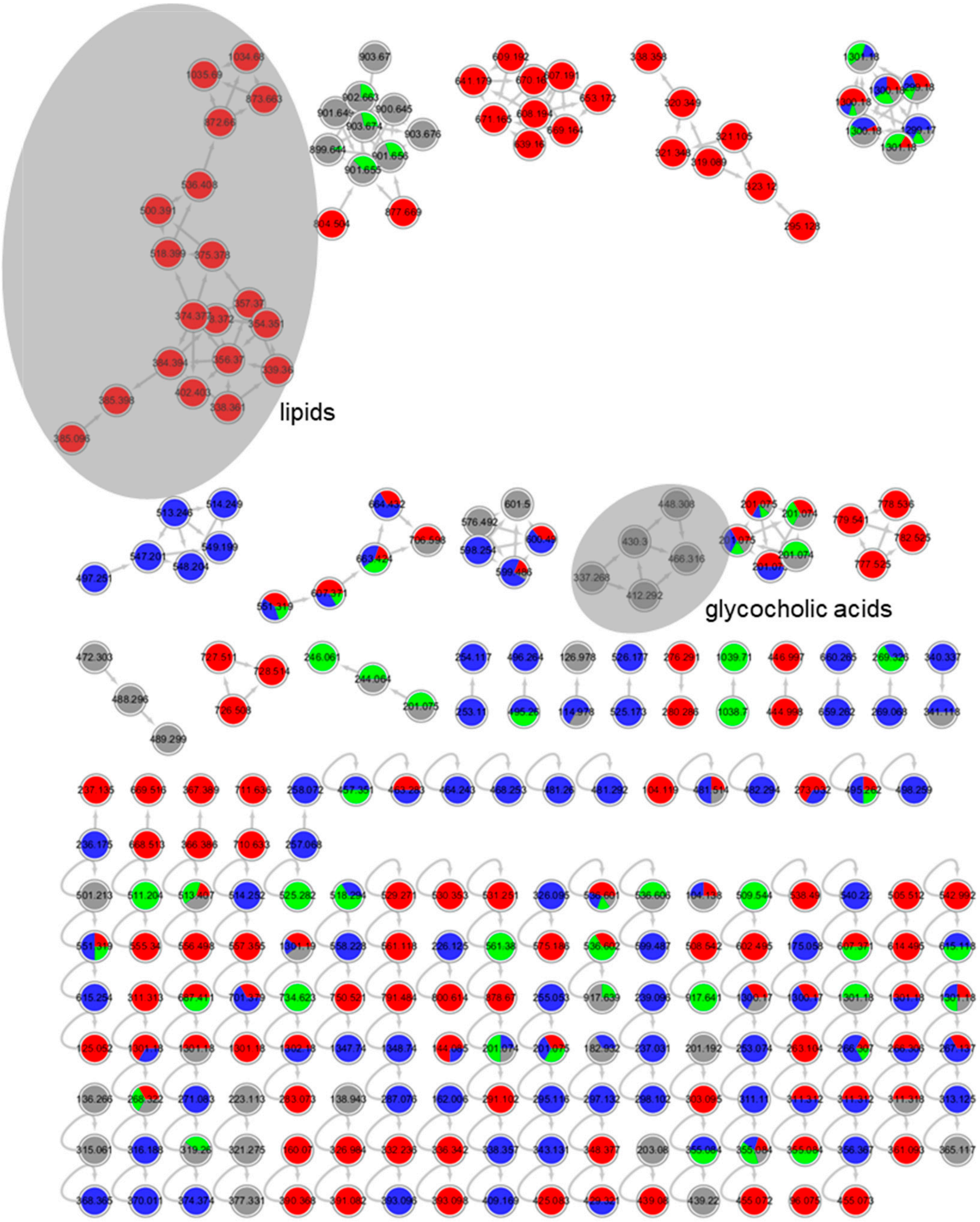

Figure S17. Annotated molecular network for strain 59 (order Glomerellales) solid culture extracts in PDM-S (**blue**), Cza-S (**red**), SYM-S (**green**), WM-S (**grey**). Annotated peak ions of putatively known compounds are highlighted by a grey loop.

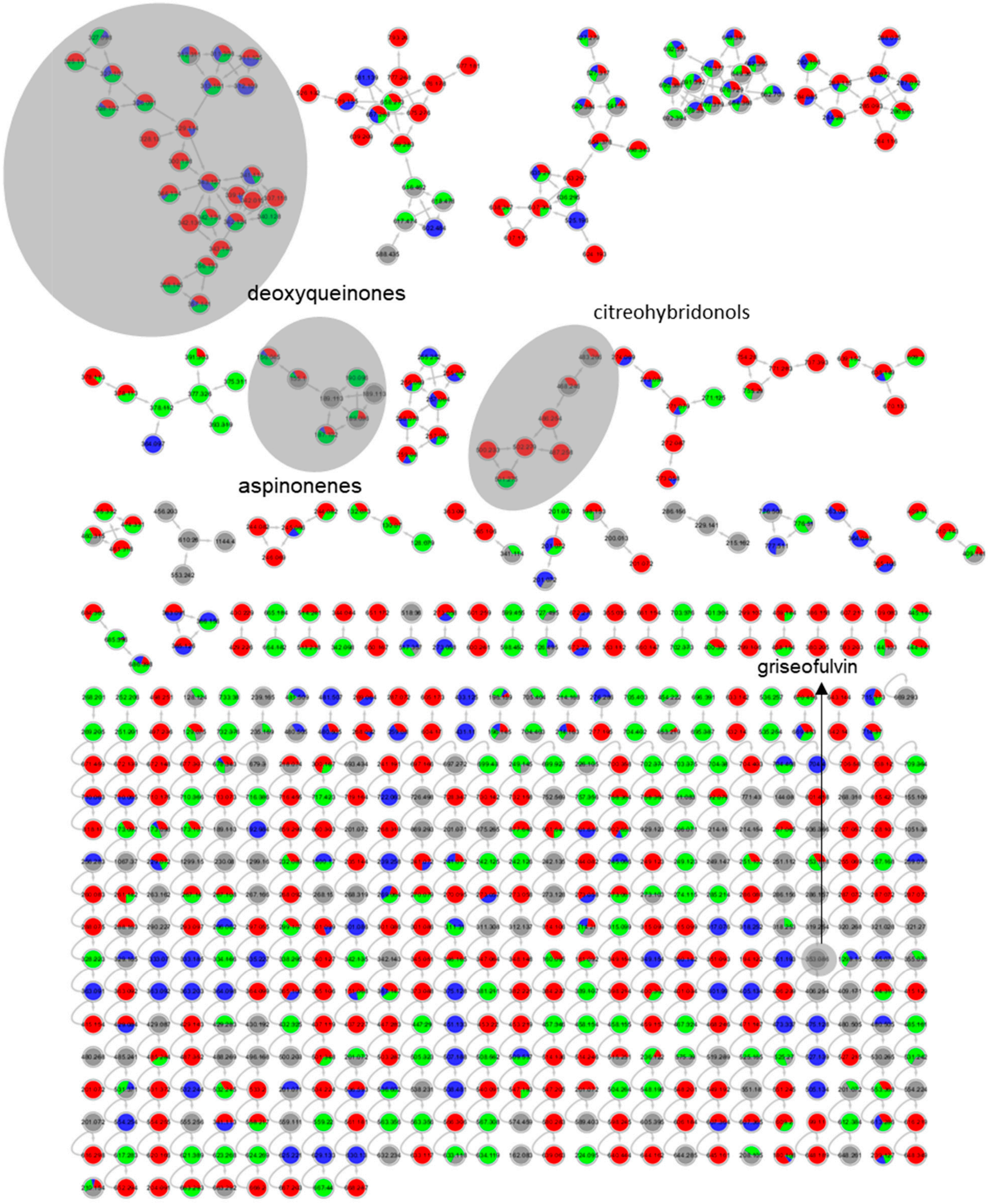

Figure S18. Annotated molecular network for strain 68 (*Penicillium* sp.) liquid culture extracts in PDM-L (**blue**), Cza-L (**red**), SYM-L (**green**), WM-L (**grey**). Annotated peak ions of putatively known compounds are highlighted by a grey loop.

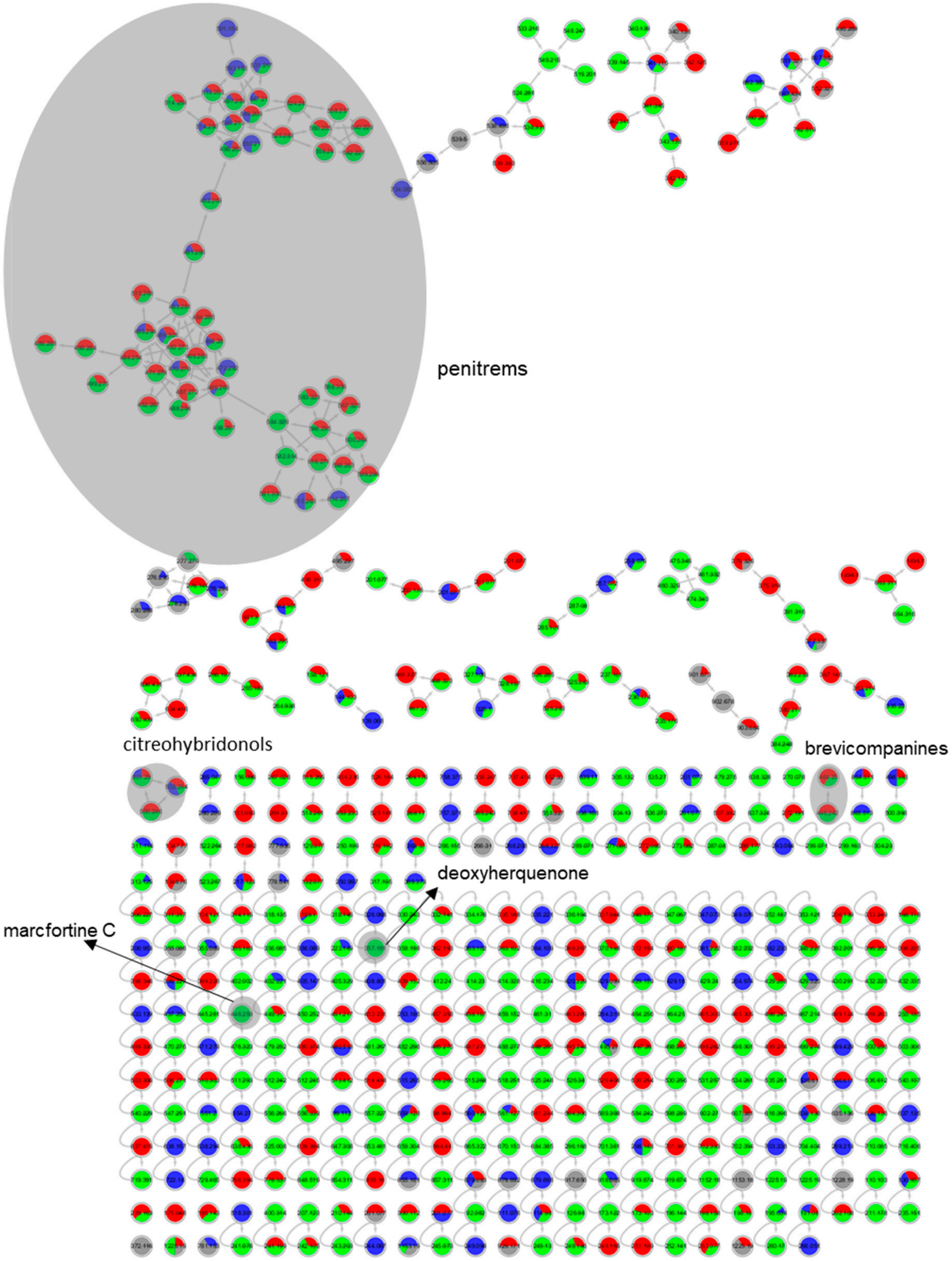

Figure S19. Annotated molecular network for strain 68 (*Penicillium* sp.) solid culture extracts in PDM-S (**blue**), Cza-S (**red**), SYM-S (**green**), WM-S (**grey**). Annotated peak ions of putatively known compounds are highlighted by a grey loop.

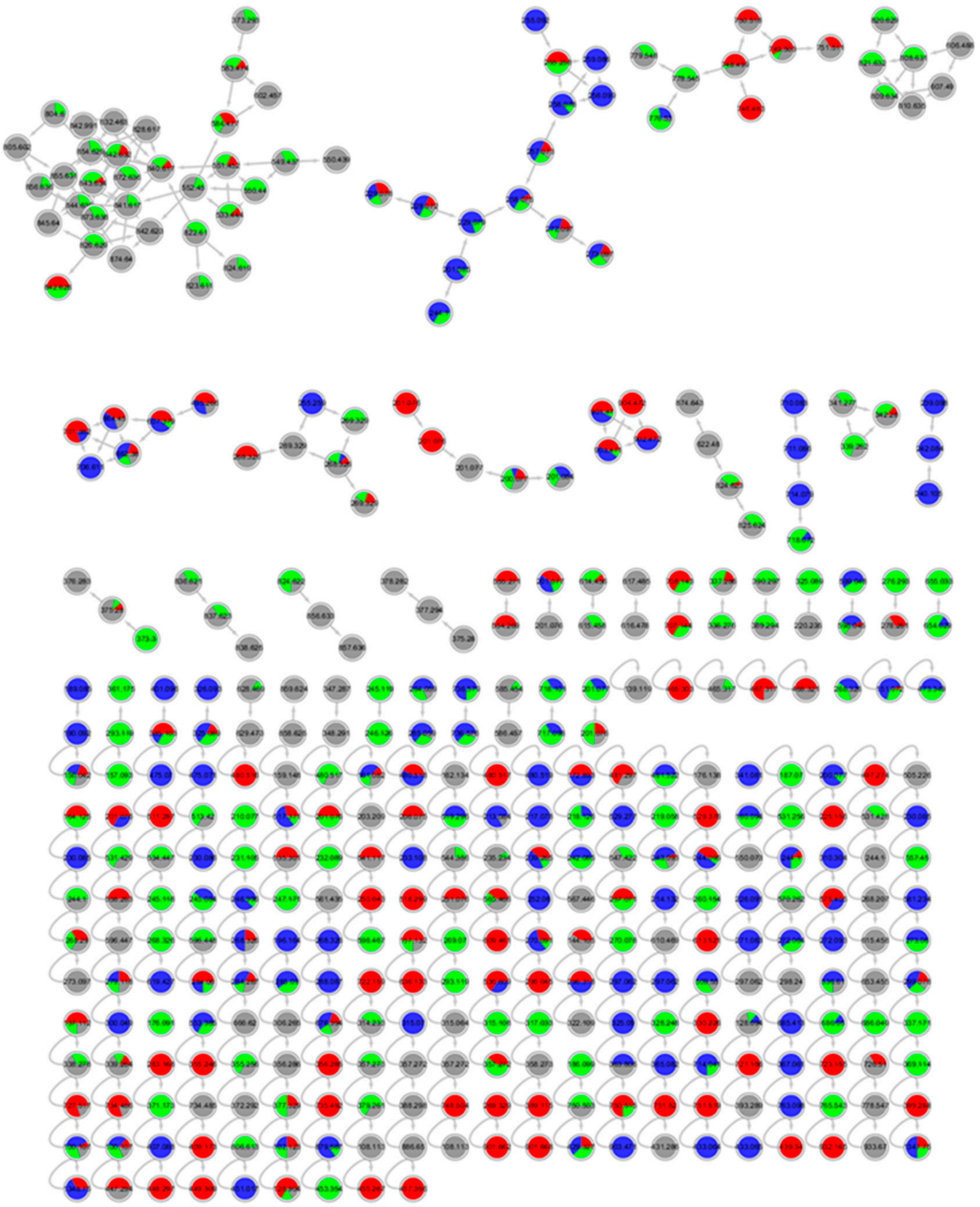

Figure S20. Annotated molecular network for strain 78 (*Penicillium* sp.) liquid culture extracts in PDM-L (**blue**), Cza-L (**red**), SYM-L (**green**), WM-L (**grey**).

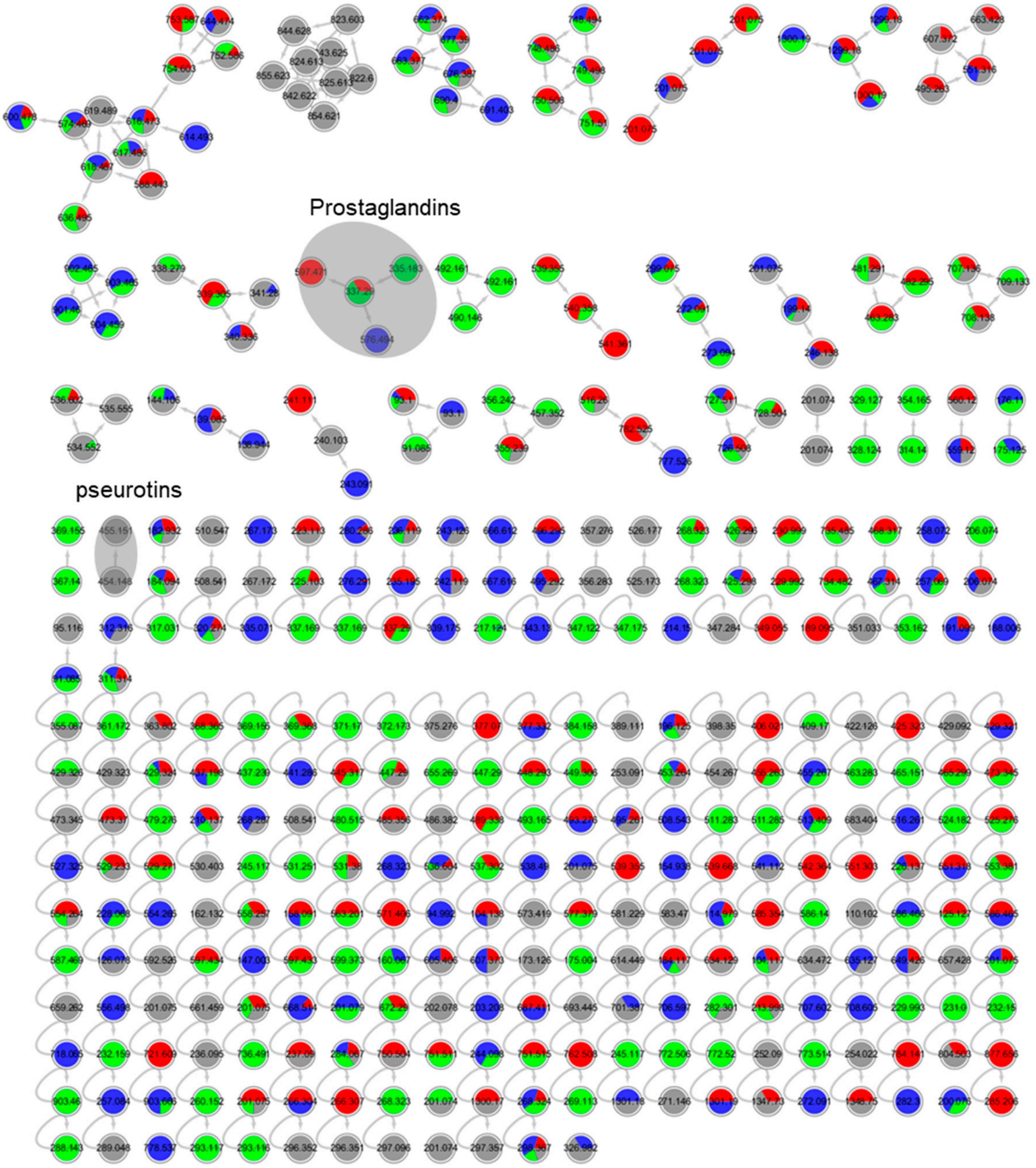

Figure S21. Annotated molecular network for strain 78 (*Penicillium* sp.) solid culture extracts in PDM-S (**blue**), Cza-S (**red**), SYM-S (**green**), WM-S (**grey**). Annotated peak ions of putatively known compounds are highlighted by a grey loop.

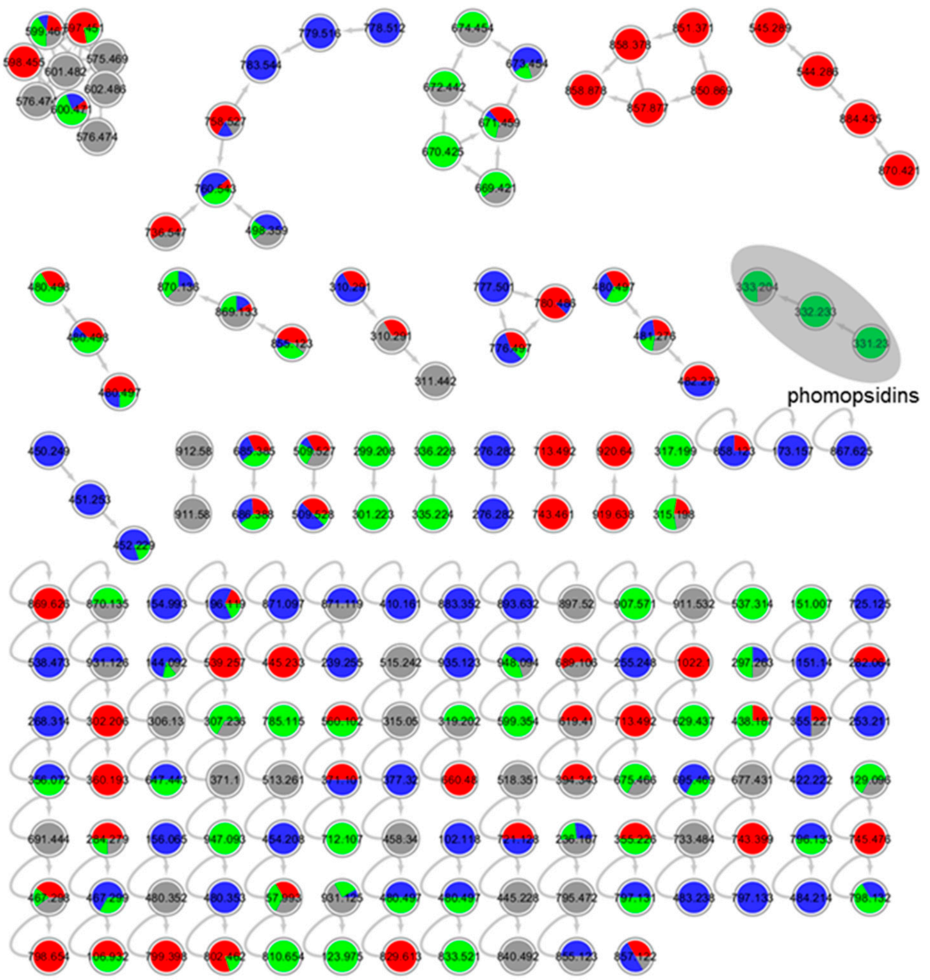

Figure S22. Annotated molecular network for strain 87 (order Pleosporales) liquid culture extracts in PDM-L (**blue**), Cza-L (**red**), SYM-L (**green**), WM-L (**grey**). Annotated peak ions of putatively known compounds are highlighted by a grey loop.

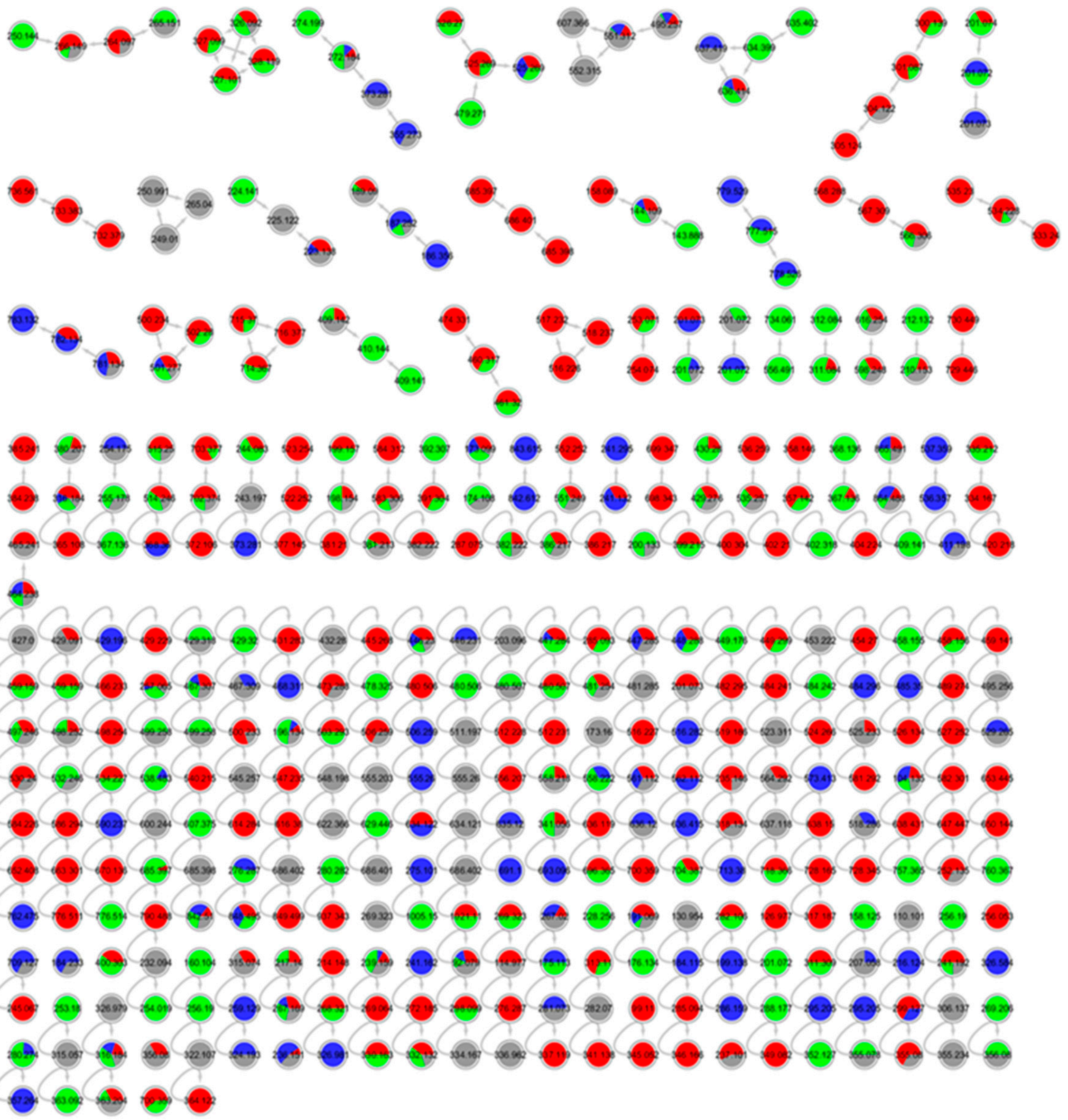

Figure S23. Annotated molecular network for strain 87 (order Pleosporales) solid culture extracts in PDM-S (**blue**), Cza-S (**red**), SYM-S (**green**), WM-S (**grey**).

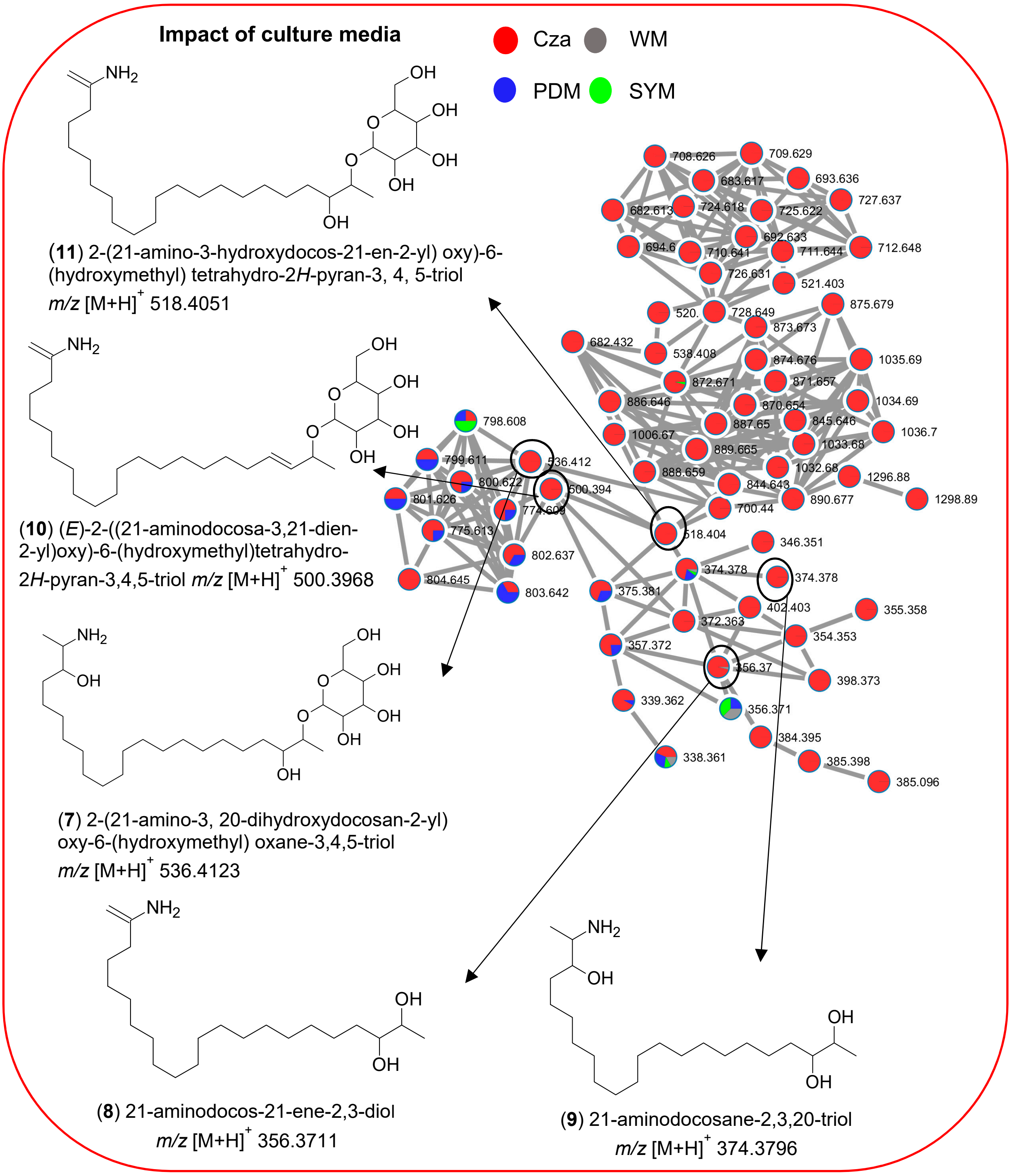

Figure S24. Molecular network of the aminolipid family detected in strain 59 (order Glomerellales) extracts. **Red nodes:** Ions detected in Cza extracts. **Blue nodes:** Ions detected in PDM extracts. **Green nodes:** Ions detected in SYM extracts. **Grey nodes**: Ions detected in WM extracts.

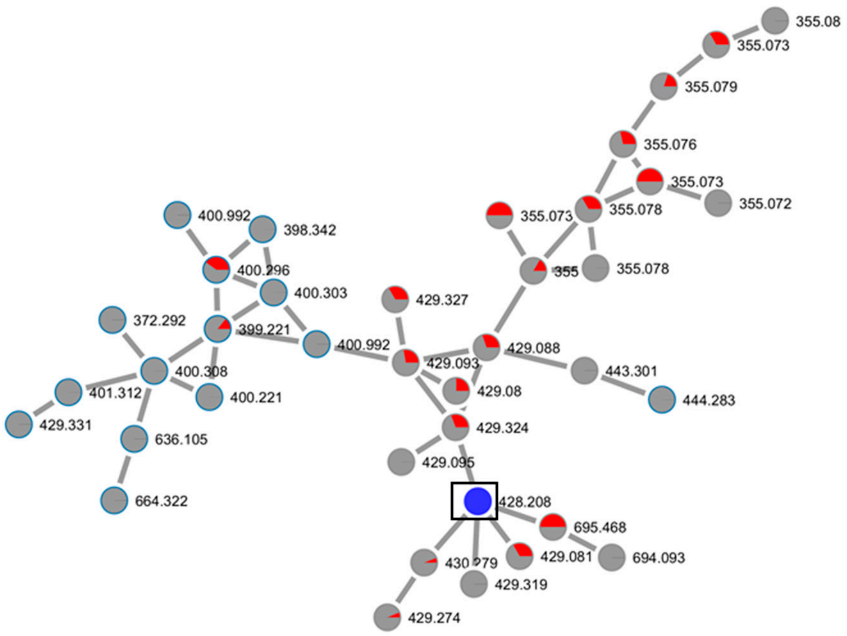

Figure S25. Molecular network of an unannotated cluster containing specific node (*m*/*z* [M+H]+ 428.2081) generated from the global MN (Figure 7A). **Black square**: node exclusive to the bioactive 1PDM-L extract. **Red nodes:** Ions detected in toxic samples. **Grey nodes**: Ions detected in inactive samples. **Blue nodes:** Ions detected in bioactive samples.

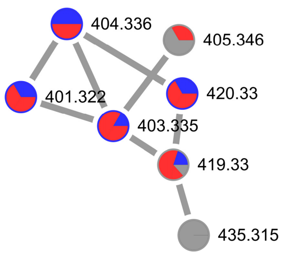

Figure S26. Molecular network of an unannotated cluster containing 3 specific nodes (*m*/*z* $[M+H]^+$ 401.3221, $[M+H]^+$ 403.3355, $[M+H]^+$ 419.3300) generated from the global MN (Figure 7A). **Red nodes:** Ions detected in toxic samples. **Grey nodes**: Ions detected in inactive samples. **Blue nodes:** Ions detected in bioactive samples.
